# Advances in Xanthine Oxidase Inhibition: A Review of Potential Bioactive Synthetic Compounds

**DOI:** 10.1002/ardp.70079

**Published:** 2025-08-21

**Authors:** Giorgio Antoniolli, Gabriel Rodrigues de Moraes, Rafael Porreca Neves da Costa, Giovani Augusto Rosendo de Campos, Fernando Coelho

**Affiliations:** ^1^ Institute of Chemistry University of Campinas Campinas São Paulo Brazil; ^2^ Faculty of Pharmaceutical Sciences University of Campinas Campinas São Paulo Brazil

**Keywords:** hyperuricemia, inhibitor, molecular docking, synthetic, xanthine oxidase

## Abstract

Hyperuricemia and gout are conditions that continue to present significant therapeutic challenges, mainly due to limitations of current treatments which may cause adverse effects such as hypersensitivity, hepatotoxicity, and cardiovascular risks. Additionally, the development of new drugs faces obstacles including toxicity, low bioavailability, and potential drug interactions. In this context, the search for new synthetic xanthine oxidase inhibitors is essential to improve treatment efficacy, selectivity, and safety. New molecules can not only prolong the duration of therapeutic effects but also minimize adverse reactions. Moreover, several compounds under investigation exhibit additional pharmacological activities, such as anti‐inflammatory, antioxidant, anticholinesterase, and anticancer properties. Recent research trends also include the use of artificial intelligence, computational modeling, and the exploration of natural products—such as flavonoids and alkaloids—as sources of inspiration for new synthetic inhibitors. Furthermore, strategies like combination therapies are being explored to enhance uric acid excretion. This review conducted a systematic search for new xanthine oxidase inhibitors (2025–2020), focusing on high‐impact publications and organizing the compounds according to their molecular scaffolds (number of rings). This review highlights recent advances in the development of synthetic xanthine oxidase inhibitors, emphasizing their structural diversity and the continuous relevance of this study area. The discovery of new drug candidates has the potential to provide more effective and safer therapeutic options for patients in the near future.

## Graphical abstract

This review article looks at new synthetic xanthine oxidase inhibitors discovered in the last 5 years (2025–2020). It covers an analysis of the advances, structural diversity, biological potential of the molecules investigated and future prospects in drug development.

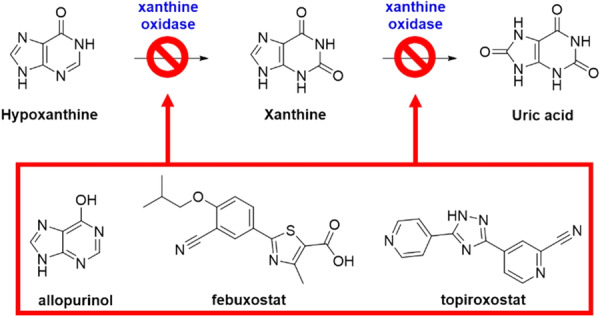

## 1 Introduction

**Figure 1:**
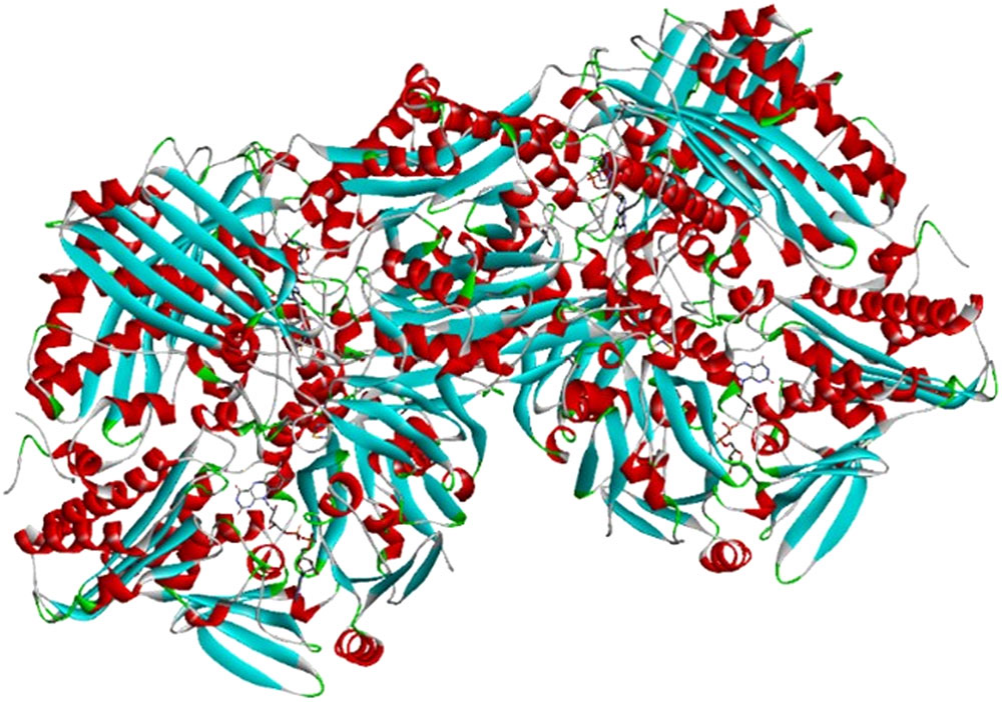
Representation of the homodimer of the xanthine oxidase enzyme (PDB: 3BDJ).

**Figure 2:**
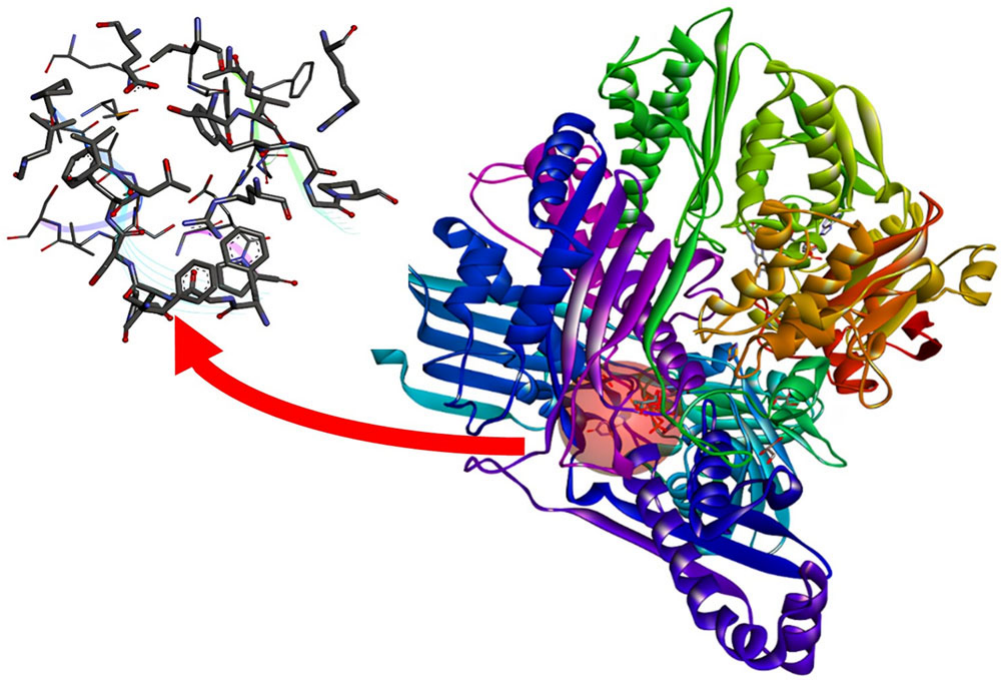
Representation of the “B” part of the homodimer of the xanthine oxidase enzyme (PDB: 3BDJ) with the active site highlighted.

Xanthine oxidase (XO) is a homodimer with a molecular mass of 290 kDa. The enzyme belongs to the molybdenum‐containing protein family, containing a molybdenum, one of the flavin adenine dinucleotides (FAD) and two ferredoxin‐type iron–sulfur centers (2Fe–2S) in each of its independent subunits (Figure 1). In addition, the enzyme has two separate substrate binding sites [1, 2, 3, 4, 5] (Figure 2). XO is the only reactive oxygen species (ROS) creating enzyme in the living system, which oxidizes hypoxanthine to xanthine and then to uric acid during purine metabolism [6].

**Figure 3:**
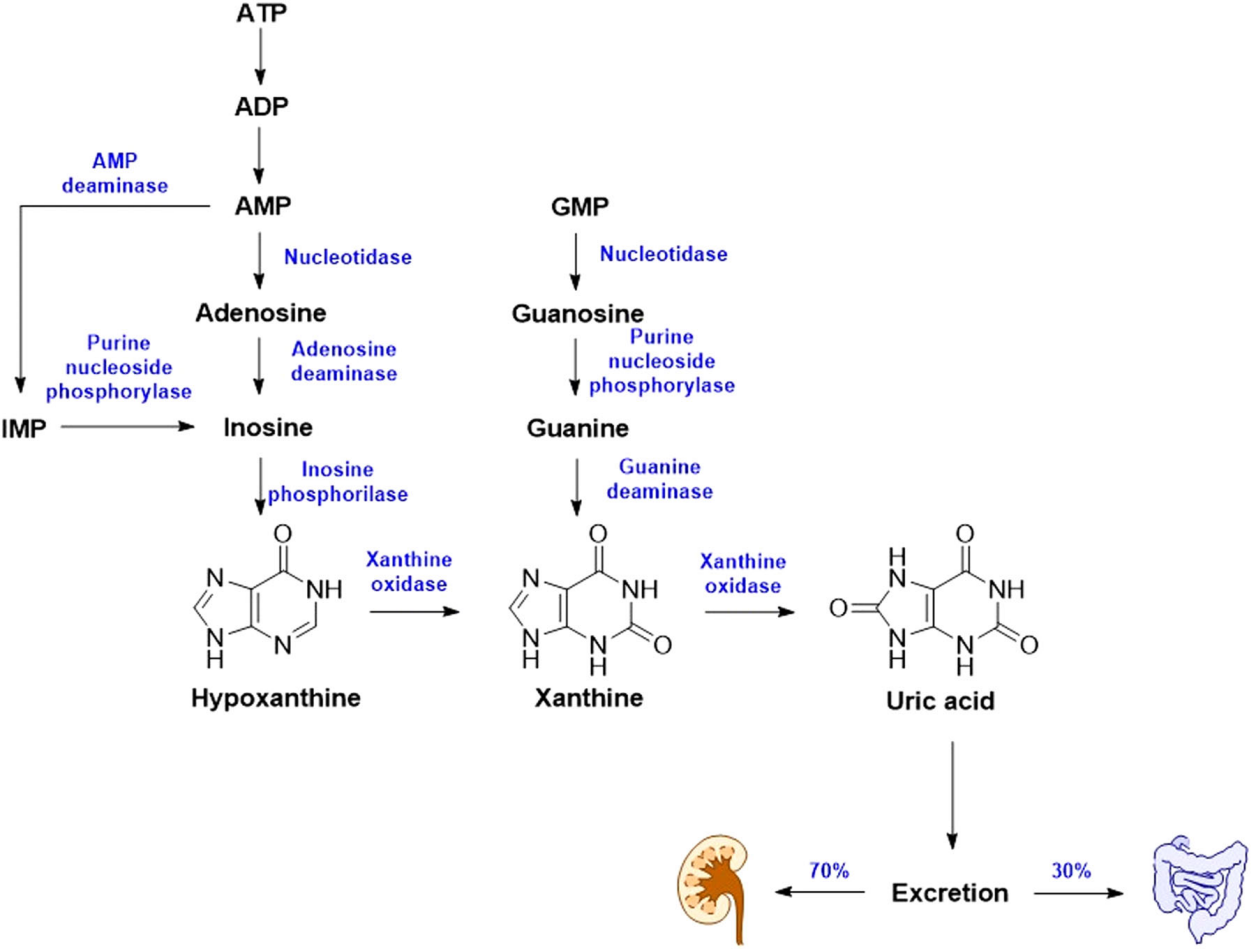
Scheme of the purine degradation pathway leading to the formation of uric acid.

ATP (adenosine triphosphate), as well as other purines, can undergo a sequential cascade of degradation, which leads to the formation of hypoxanthine and then its conversion into xanthine. Finally, xanthine is irreversibly transformed into uric acid by the action of XO [7] (Figure 3). XO oxidizes xanthine with the help of the cofactor molybdenum, transferring the electron via two Fe–S clusters to the FAD coenzyme part to reduce NAD^+^ (nicotinamide adenine dinucleotide) to NADH (nicotinamide adenine dinucleotide, reduced). The electron flow affinity of NAD^+^ at the FAD site decreases and the affinity for oxygen increases during the hypoxic condition. This will cause univalent and divalent electron transfer generating superoxide or hydrogen peroxide [8, 9]. For this reason, XO is considered one of the sources of peroxide in cells and is associated with hypoxic injury [10, 11].

Uric acid is the end product of purine metabolism in the human body. Concerted effects by hepatic production, intestinal secretion, renal reabsorption and secretion maintain a homeostasis of serum uric acid. A sustained high level of serum uric acid leads to the chronic metabolic disorder hyperuricemia, which is not only the cause of gout, but is also associated with other diseases, including kidney disorder, metabolic syndrome and cardiovascular disease. Hyperuricemia is a symptom of high uric acid levels, accompanied by abnormal purine metabolism in the body. Prolonged supersaturation of uric acid leads to the formation and deposition of monosodium uric acid crystals, which eventually develop into obstructive nephropathy, kidney stones, and gout [12].

XO is a key enzyme in the pathogenesis of hyperuricemia. The first‐line treatment of hyperuricemia involves the use of drugs that inhibit the activity of XO. This enzyme is responsible for the production of uric acid in the body. Currently approved drugs suffer from serious side effects. The search for new XO inhibitors covered a broad spectrum of heterocyclic scaffolds and identified several promising compounds [13, 14]. It should be noted that inhibition of this enzyme reduces vascular oxidative stress and also circulating levels of uric acid.

Allopurinol is a prototypical purine‐like XO inhibitor and has been prescribed in the treatment of hyperuricemia and gout for several decades, approved by the FDA in 1966 [15]. However, its nonselective inhibition, low values and the variability of its potency (IC_50_: 0.2–50 μM, half maximal inhibitory concentration) [16] and require extremely high dosages (800–900 mg/day) [17]. Allopurinol, which is an 8‐aza‐7‐deaza isoster of hypoxanthine, remains the drug of choice in the treatment of hyperuricemia and gout [18]. The drugs topiroxostat and febuxostat are non‐purine XO inhibitors [19, 20]. Most of the research published over the last two decades has focused on identifying non‐purine inhibitors, resulting in the approval of two drugs, febuxostat and topiroxostat [21].

Therefore, the prevalence of hyperuricemia has become a global health problem and effective therapeutic agents are urgently needed. However, the clinical application of these drugs is hampered by undesirable side effects. For example, adverse drug hypersensitivity reactions occur in the clinical use of allopurinol [22] and hepatotoxicity, as well as cardiovascular risks, were observed during the application of febuxostat [23].

However, it is always important to highlight the prevalence of research involving new possible inhibitors, which in this case are for XO inhibitors. It is important to note that between 2015 and 2019 there was extensive research into possible new candidates for XO inhibitors [24, 25, 26, 27, 28, 29, 30, 31, 32, 33, 34, 35, 36, 37, 38, 39, 40, 41, 42, 43]. Therefore, the main objective of this review is to highlight the latest discoveries of bioactive synthetic substances on XO inhibition and increase understanding of the potential uses of new molecules to treat hyperuricemia over the last 5 years. Furthermore, this review aims to assist academic and nonacademic circles by demonstrating the constant search for new XO inhibitors. We have divided the section on the investigation of new inhibitors according to the number of cycles (the general structure or general skeleton of the molecule) covered by each study.

## 2 Methods

### 2.1 Concerning the Literature Review

In this review, our objective was to systematically search for, investigate, and report on the discovery of new molecules with activity against XO. To achieve this, we conducted an online search in the Scopus database using keywords such as “xanthine oxidase,” “synthesis,” “biological activity,” and “inhibition.” We carried out the search retrospectively, starting from the year 2025 back to 2020, selecting articles with strong results published in high‐impact journals [44].

The results obtained during the online search were organized by categorizing the studies based on the number of rings (i.e., the general structure or molecular scaffold).

### 2.2 Study of the Predicted Physicochemical, Pharmacokinetic, and Toxicological Properties

SwissADME software was used to predict the properties of the compounds [45]. A list of properties was evaluated, including: molecular weight, number of rotatable bonds, hydrogen bond acceptors, hydrogen bond donors, molar refractivity, topological polar surface area, logarithm of solubility (ESOL), average of the logarithm of the partition coefficient (Consensus), inhibition of the main cytochrome P450 (CYP450) isoforms (1A2, 2C9, 2C19, 2D6, and 3A4), gastrointestinal absorption, blood‐brain barrier permeability and inhibitor or substrate for P‐glycoprotein. ProTox 3.0 software was used to predict the toxicity of the compounds (1–93) and the three drugs (allopurinol, febuxostat, and topiroxostat) [46]. See the Supporting Information: File S1, which contains this information.

## 3 Investigation of New Molecules as XO Inhibitors

### 3.1 Two Cycles

**Figure 4:**
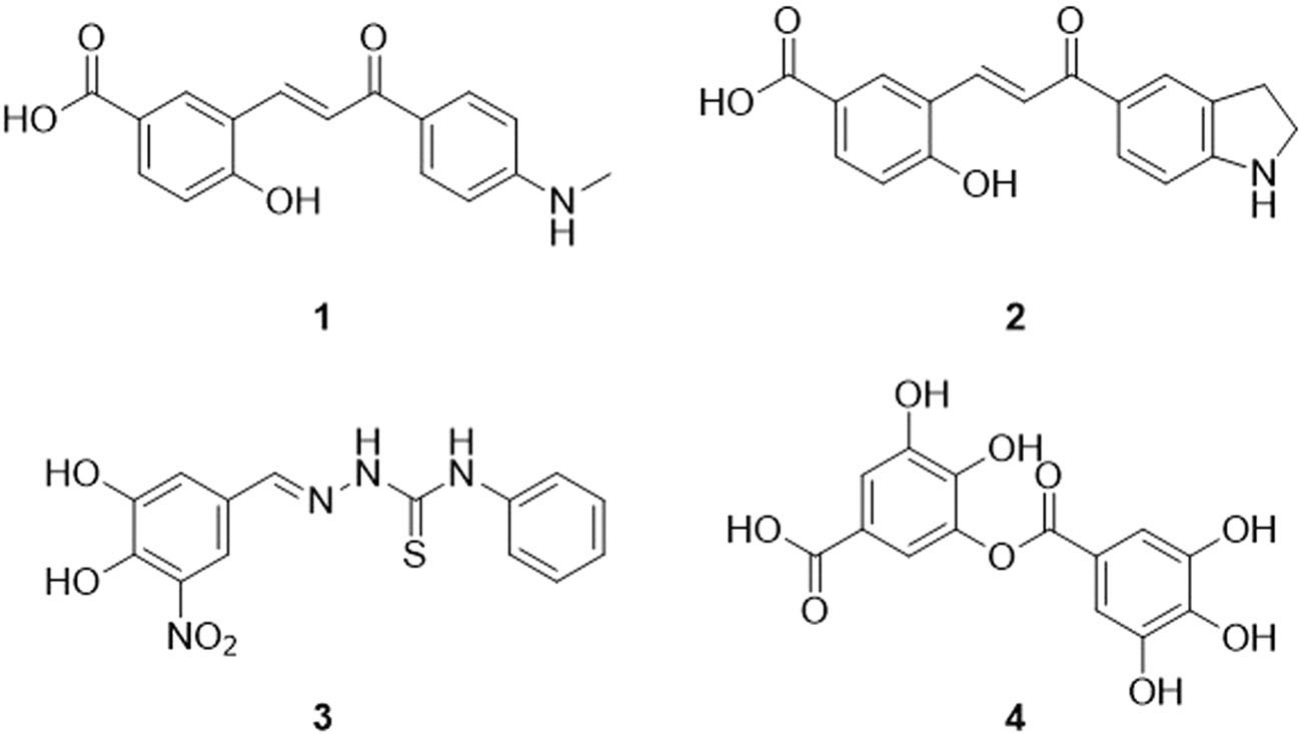
Structures of compounds **1**–**4** as potential XO inhibitors.

A study developed by Xu et al. evaluated chalcone derivatives with potential XO inhibitory activity. The most promising compounds in the study, **1** and **2**, showed inhibitory activities with IC_50_ values of 0.102 and 0.064 μM, against 2.588 and 0.028 μM of the positive controls, alupurinol and febuxostat (Figure 4). Studies were conducted to verify the kinetics of XO inhibition, which showed that the compounds are mixed‐type inhibitors and that they bind to free XO enzymes with high affinities, which may offer a promising therapeutic prospect for the treatment of hyperuricemia. Molecular docking studies revealed the formation of hydrogen bonds between the carboxyl and hydroxyl groups of the B ring and specific amino acid residues, Arg880, Thr1010 and Glu802. In the in vivo studies, they chose **1** and showed that in the high dose group (40 mg.kg^−1^), the efficacy of **1** was comparable to that of the drug allopurinol, and biochemical assays showed that the compound did not cause liver or kidney impairment in the short term [47].

A study conducted by Yu and collaborators synthesized a series of benzaldehyde thiosemicarbazone as potential XO inhibitors. Compound **3**, (*E*)‐2‐(3,4‐dihydroxy‐5‐nitrobenzylidene)‐*N*‐phenylhydrazine‐1‐carbothioamide, was potent in the order of nM, with an IC_50_ value of 0.0437 μM, being 734 times more potent than its analog 3,4‐dihydroxy‐5‐nitrobenzaldehyde (DHNB) and 173 times more potent than the drug allopurinol, demonstrating its excellent inhibitory activity (Figure 4). In the evaluation of enzyme kinetics, the compound showed high potency in vitro and a mixed competitive effect on XO. At moderate doses of **3**, the inhibitory effects were significant and decreased the animals' uric acid concentration to close to normal levels. In addition, this class of compounds showed no acute toxicity. Overall, **3** exhibited safer and more potent XO inhibitory activity than the drug allopurinol. This molecule has great potential to become a candidate for the treatment of hyperuricemia and gout [48].

Zheng and colleagues discovered digallic acid, **4**, as a dual inhibitor of XO and urate transporter 1 (URAT1) as a potential use for treating uricemia. The acid showed an IC_50_ of 1.04 ± 0.23 μM, better than the drug allopurinol, but not as potent as febuxostat, for XO (Figure 4). When evaluated in HEK293 cells with URAT1 overexpression, digalic acid showed an IC_50_ of 5.34 μM, with an intermediate value between the two positive controls, benzobromarone and lesinurad. In vivo studies showed that digallic acid effectively reduced urate levels and promoted uric acid excretion, and with this, safety assessments did not show any kidney damage associated with treatment with digallic acid [49].

**Figure 5:**
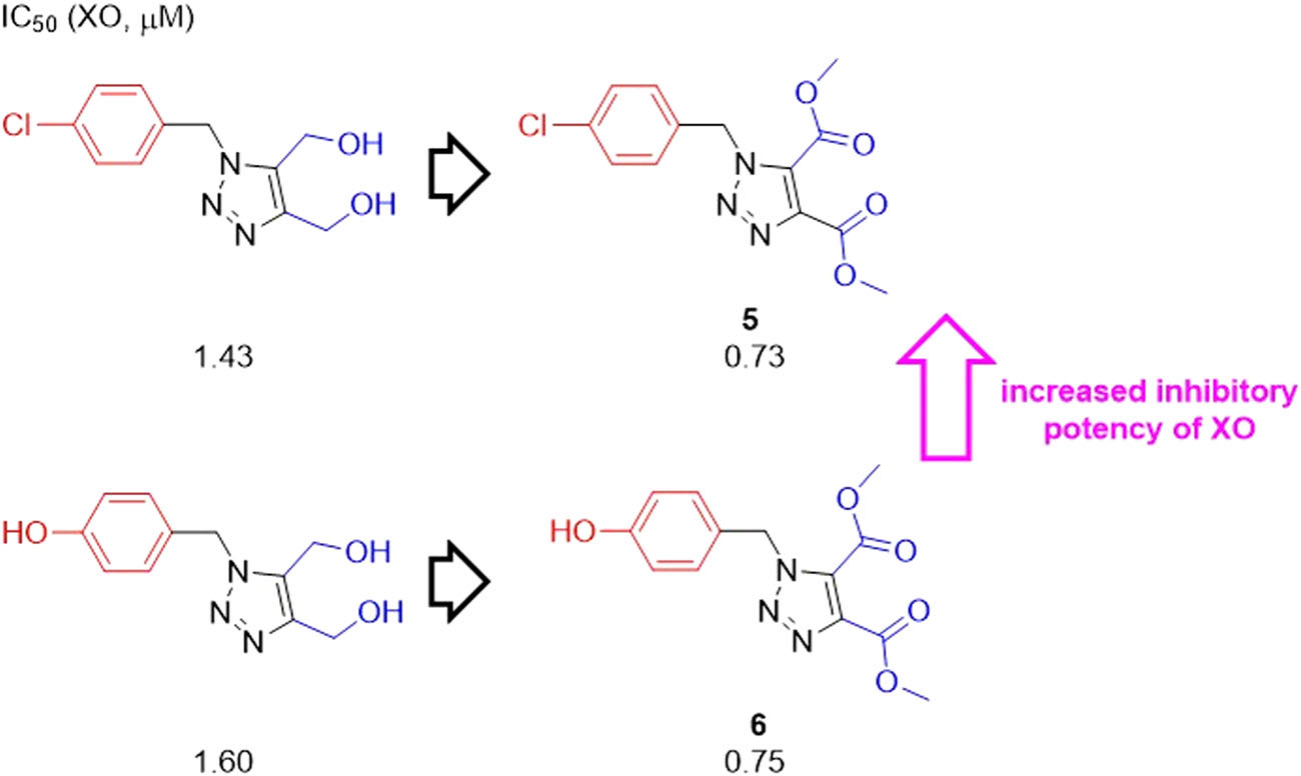
Schematic representation of the difference between dimethanol and dicarboxylate.

An interesting study conducted by Yagiz and co‐workers evaluates dimethyl *N*‐benzyl‐1*H*‐1,2,3‐triazole‐4,5‐dicarboxylate and (*N*‐benzyl‐1*H*‐1,2,3‐triazole‐4,5‐diyl)dimethanol as potential XO inhibitors. Two compounds had satisfactory IC_50_ values, **5** and **6**, but in addition to these, (1‐(3‐bromobenzyl)‐1*H*‐1,2,3‐triazole‐4,5‐diyl)dimethanol exhibited an IC_50_ of 0.71 µM (Figure 5). For the purposes of comparing the activity between dimethanol and dicarboxylate compounds, we observed that dicarboxylate compounds are more active for XO than dimethanol, unlike (1‐(3‐bromobenzyl)‐1*H*‐1,2,3‐triazole‐4,5‐diyl)dimethanol, which the dicarboxylate derivative exhibited a worsening in the IC_50_ value. Molecular docking studies revealed that **6** established a hydrogen bond with the Thr1010 residue. It is interesting to note that the presence of a ‐Br, ‐Cl or ‐OH substituent in the para or meta positions of the benzene ring facilitates an orientation that allows complex stabilization by pi interactions with the two rings [50].

**Figure 6:**
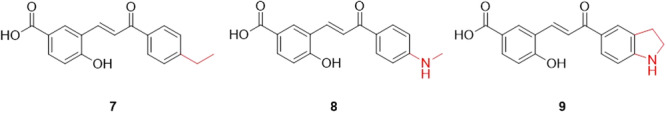
Chalcones as possible xanthine oxidase (XO) inhibitors.

When studying hyperuricemia, Xu et al. pointed out that, due to the low number of options and the large number of related side effects, it would be of great interest to synthesize new compounds capable of inhibiting XO. To this end, 35 drug candidates were planned and synthesized, all chalcone analogs, since chalcone already has some biological activity related to XO inhibition. To synthesize the compounds, alkoxide, alkyl and heterocycle groups were added to ring A of the chalcone structure to maximize the enzyme inhibition activity. The compounds synthesized showed satisfactory activities against XO (IC_50_ = 0.064–0.559 μM), all of which were superior to allopurinol (IC_50_ = 2.588 μM), a drug already used to treat hyperuricemia. Among the compounds synthesized, we highlight compounds **7** (IC_50_ = 0.084 μM), **8** (IC_50_ = 0.102 μM), and **9** (IC50 = 0.064 μM), which are good inhibitors and meet the criteria of Lipinski's rules for oral administration as drugs (Figure 6). Furthermore, after tests on AML12 cells (normal mouse hepatocytes), it was found that compounds **8** and **9** did not show significant cytotoxicity, while compound **7** showed little cytotoxicity. Moving on to in vivo tests, compound **8** stood out, showing greater results against hyperuricemia in rats, and could be compared to allopurinol when administered at a high dose (40 mg.kg^−1^). In conclusion, the study provides good results and presents a new possible drug candidate for the treatment of hyperuricemia [47].

**Figure 7:**
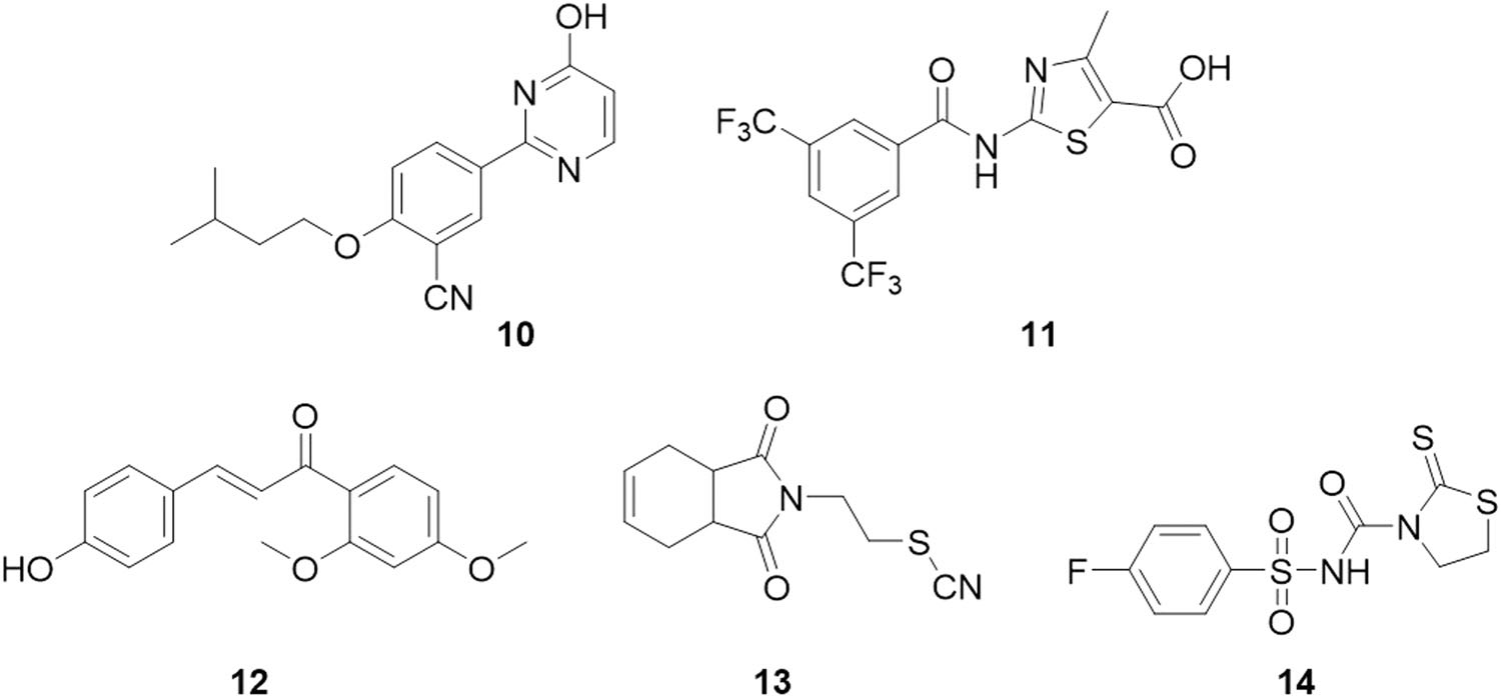
Structures of compounds **10**–**14** as potential xanthine oxidase (XO) inhibitors.

Sun and collaborators, with the aim of synthesizing a non‐purine compound that could inhibit the XO enzyme, proposed pyrimidine derivatives, a class of compounds widely used in medicinal chemistry with previously reported inhibitory potency, combined with amino or hydroxy groups as pharmacophores in their development. Most of the compounds synthesized showed good inhibitory activities, the best of which was compound **10**, which had an IC_50_ value of 0.046 µM (Figure 7). It was noted that the addition of the hydroxy group increased the inhibitory potency, as did the amino group, however when combined they did not result in the sum of the individual effects. When subjected to enzyme kinetics tests and compared to allopurionol, it was concluded that its inhibitory activity occurs in a mixed way. In vivo tests were then carried out on a hyperuricemic rat model, whose references for uric acid analysis were allopurinol and febuxostat. The study showed a significant capacity for reducing compound **10**, and therefore suggests that the compound is promising in the treatment of hyperuricemia [51].

A series of compounds derived from thiazole‐5‐carboxylic acid was designed and synthesized by Kaur and colleagues. The most potent compound in the study, **11**, exhibited XO inhibitory activity with an IC_50_ value of 0.45 µM (Figure 7). structure–activity relationship studies showed that B‐ring disubstituted compounds were more potent than monosubstituted derivatives. **11** has been described as a mixed‐type inhibitor. The carboxylate group on the thiazole ring showed two hydrogen bond interactions with the Arg880 residue, as well as another hydrogen bond with the Ser875 residue via the amide carbonyl. The study suggests that **11** may block the activity of XO sufficiently to prevent the substrate from binding to its active site [52].

Chalcone derivatives were evaluated by Yang et al. as potential XO inhibitors. Compound **12** was the most potent inhibitor with an IC_50_ value of 0.121 µM, more potent than the drug allopurinol and not as potent as the drug febuxostat (Figure 7). structure–activity relationship studies were important in determining that the introduction of hydroxyl groups at the 3′/4′/5′ positions on ring A was more efficient than at the 2′/6′ positions for XO inhibition, and that the removal of the 3‐hydroxyl group on ring B could weaken XO inhibitory potency in hydroxychalcones, but be beneficial for XO inhibitory potency in methoxychalcones. In molecular docking studies, the hydroxyl group of ring B formed two hydrogen bonds with residue Arg880 and the methoxyl group of ring A formed one hydrogen bond with residue Asn768. In vivo studies showed that compound **12** did not have as significant a hypouricemic effect as allopurinol, since it had a homologous hypouricemic effect with allopurinol from 1 to 5 h. The ADMET (absorption, distribution, metabolism, excretion, and toxicity) prediction study showed that compound **12** exhibited adequate physicochemical, pharmacokinetic, and safety properties when compared to allopurinol and febuxostat. Compound **12**, when compared to allopurinol and febuxostat, also demonstrated good performance in Caco‐2 permeability, intestinal absorption, blood‐brain barrier penetration, and plasma protein binding, which suggests its good absorption and distribution capacity. Furthermore, toxicity prediction studies indicated that all three compounds are hepatotoxic, but compound **12** could also be nephrotoxic [53].

Tan et al. developed new compounds derived from isoindol‐1,3(2*H*)‐dione containing a 1*H*‐tetrazole moiety, in which their inhibitory properties against XO and carbonic anhydrase (hCA I and hCA II) were analyzed. The featured molecule, **13**, proved to be an XO inhibitor, and with an IC_50_ value of 4.261 μM, the compound was more potent than allopurinol, the drug used as a positive control with an IC_50_ value of 4.678 μM (Figure 7). From the results of the structure activity relationship, the presence of the SNC group linked to the N‐alkyl group showed better activity. According to the molecular docking studies, the isoindol group formed two hydrogen bonds with Thr440 and Val441. The portion also formed π‐alkyl interactions with residues Phe439, Phe344, in addition, the compound formed van der Waals interactions and was more surrounded by hydrophobic residues than hydrophilic residues of the enzyme's active site [54].

To find new potential XO inhibitors, Wang and collaborators designed and synthesized a series of compounds derived from thiazolidine‐2‐thione. Most of the compounds synthesized showed satisfactory inhibitory activity, with those with the phenyl‐sulfonamide group as a substituent standing out as having good XO inhibitory activity. Compound **14** should be singled out from the others, since it shows good inhibitory activity (IC_50_ = 3.56 μM), which is almost 2.5 times more effective than allopurinol (IC_50_ = 7.86 μM), a drug that has already been consolidated in the treatment of hyperuricemia (Figure 7). In conclusion, the study shows that electron‐withdrawing groups play a fundamental role in XO inhibition, as well as presenting a possible compound for the treatment of hyperuricemia [55].

**Figure 8:**
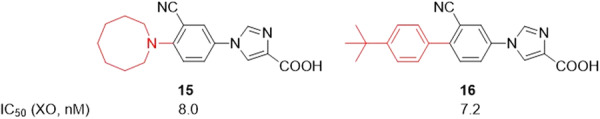
Structures of compounds **14** and **15** with their IC_50_ values, showing their potency when compared to febuxostat.

Zhou et al. synthesized derivatives of 1‐phenylimidazole‐4‐carboxylic acid as new XO inhibitors. The drug febuxostat, used as a control, showed an IC_50_ of 7.0 nM, values comparable to compounds **15** and **16** (Figure 8). In vivo studies in mice with acute and long‐term hyperuricemia induced by potassium oxonate/hypoxanthine, the compounds exhibited significant hypouricemic potencies, which were slightly weaker and similar to febuxostat, at a dosage of 5 mg. kg^−1^. Interestingly, both compounds showed the ability to improve kidney damage by decreasing creatinine and urea nitrogen levels compared to the group of mice with long‐term hyperuricemia, while febuxostat showed no significant effect [56].

**Figure 9:**
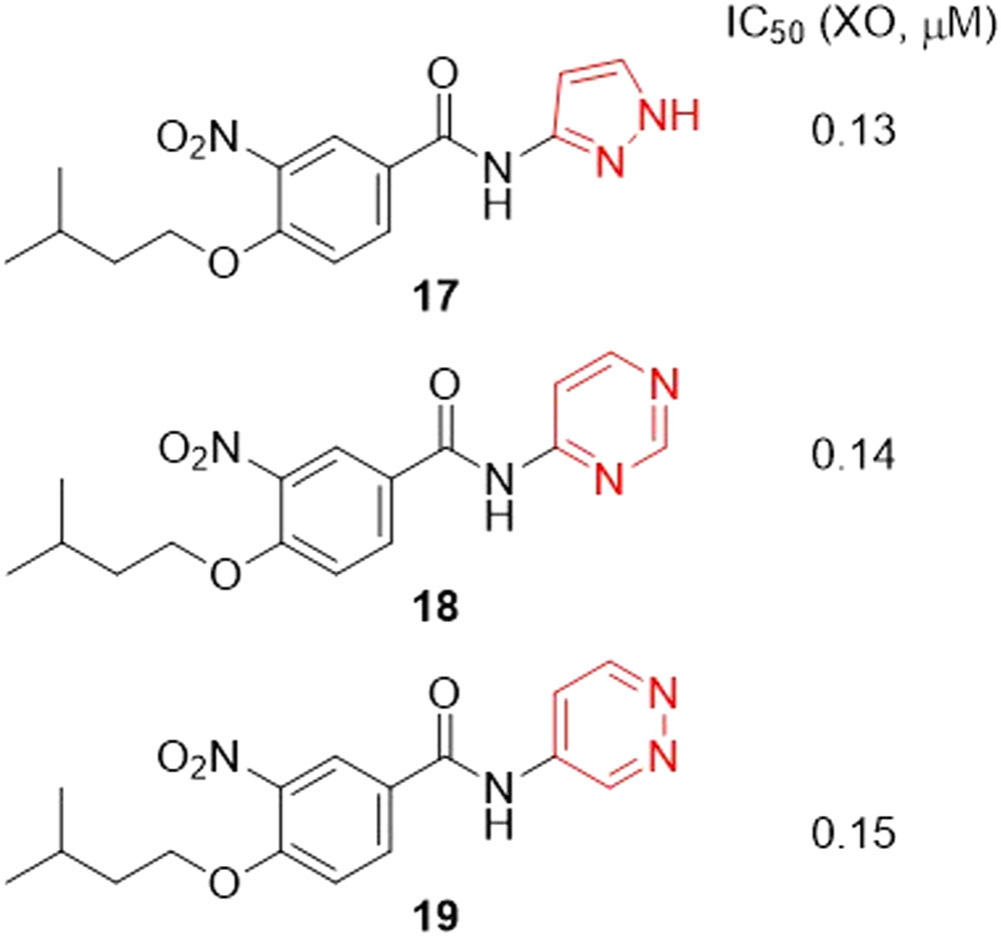
Comparison between the *N*'‐substituted amides and their respective IC_50_ values.

Guo et al. worked on the discovery of 4‐(isopentyloxy)‐3‐nitrobenzamide derivatives as possible XO inhibitors. In this study, three compounds stood out positively, with IC_50_ values ranging from 0.13 to 0.15 μM (**17**–**19**), while topiroxostat and allopurinol had IC_50_ of 0.02 and 8.80 μM, respectively (Figure 9). Molecular docking studies revealed that compound **17** has strong interactions with critical amino acid residues within the active site (by hydrogen bonds with Glu1261 and Glu802). In addition, in vivo studies have shown that **17** can reduce serum uric acid levels in animals and in vitro assays using MTT have revealed that **17** is nontoxic to healthy cells, as well as showing metabolic stability in the gastric and intestinal environments [57].

Rashad et al. synthesized new compounds derived from febuxostat encompassing carboxamide functionality and different heterocycles as XO and cyclooxygenase (COX) inhibitors. Highlight compounds **20**–**25** proved to be potential XO and/or COX inhibitors in vitro, with the most potent XO inhibitor exhibiting an IC_50_ value of 9 nM, being more potent than febuxostat (26 nM), used as a standard (Table 1). When the COX‐2 inhibition values were analyzed, IC_50_ values in the range of 0.04–0.14 µM were found, being comparable to celocoxib (0.05 µM), where **20**, **24**, and **25** were more potent than the drug itself, used as a standard. The in vivo results were in agreement with the in vitro data, as was the docking assay, whose scores were reasonable and consistent to a large extent with their corresponding in vitro values. It is important to note that the AUC (uric acid, 1–8 h) of the compounds (**20**, **21**, and **22**) and the analogs (**23** and **25**) revealed more potent hypouricemic effects than that of febuxostat. The in silico studies of the pharmacokinetic properties and predicted toxicity (ADMET), along with the ligand efficiency, suggest their potential as new candidates with a high oral safety profile. It is noteworthy that all the compounds follow Lipinski's Rule of Five, with only three compounds violating the Log *p* > 5 rule (**21**, **23**, and **25**). Another important parameter to observe is that all the compounds exhibited high human intestinal absorption, and all the tested compounds were found to be strongly bound to plasma proteins. The predicted toxicity risks indicated that none of the tested compounds showed any irritant effects, however, compound **25** exhibited a high tumorigenic potential, and compounds **21**, **24**, and **25** demonstrated a high mutagenic probability [58].

**Table 1:** Structural comparison between the compounds that were highlighted in the study with the IC_50_ values for XO and COX.

		IC_50_ (μM)		
Compound	Structure	XO	COX‐1	COX‐2
**20**	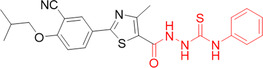	0.017	12.57	0.04
**21**	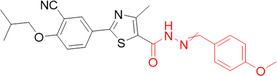	0.011	9.63	0.07
**22**	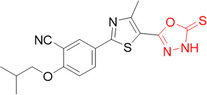	0.009	7.37	0.14
**23**	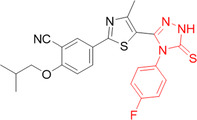	0.018	12.27	0.05
**24**	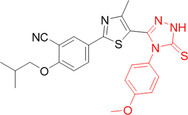	0.019	12.77	0.04
**25**	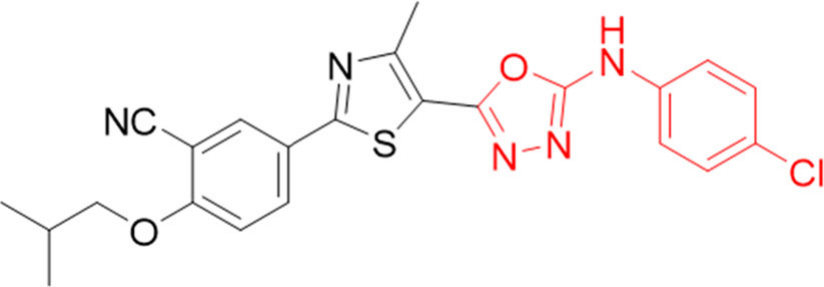	0.018	13.47	0.04

### 3.2 Three Cycles

**Figure 10:**
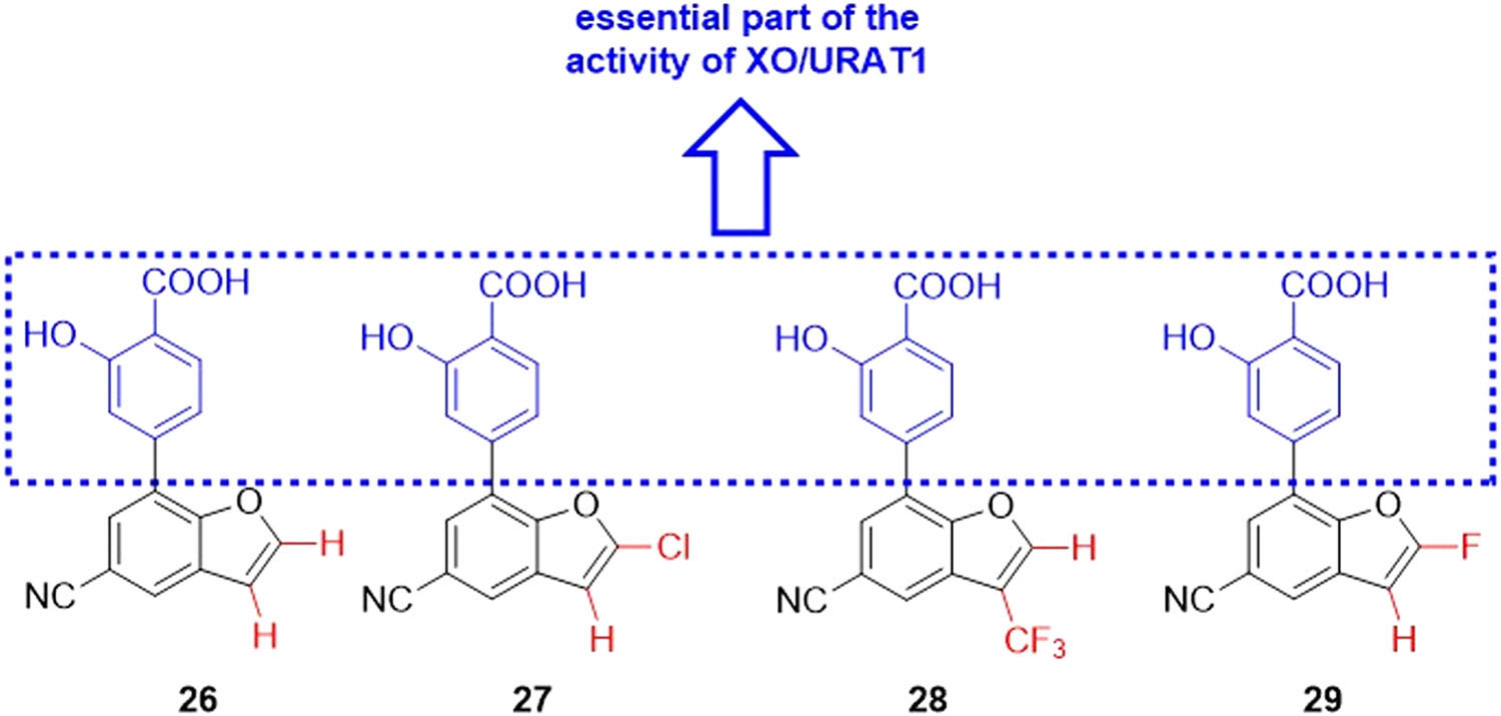
Compounds **26**–**29** with excellent activity for XO and URAT1, emphasizing the difference for the ligands in positions 2 and 3.

Yang et al. synthesized benzofuran derivatives that were quite potent as XO inhibitors. Figure 10 shows some compounds that showed significant activity in inhibiting XO and URAT1. Compound **28** exhibited an IC_50_ value of 7.5 nM, compared to febuxostat, with an IC_50_ value of 7.8 nM, both of which strongly inhibit the enzyme. The study did not go any further into the **28** molecule in question because it was not considered to be as potent an inhibitor of the urate transporter 1 as another molecule in the study. **29**, on the other hand, showed IC_50_ values of 34.6 and 31 nM for URAT1, being more potent for URAT1 than the drug used as a standard control, benzbromarone (IC_50_ = 47.6 nM). In addition, **29** demonstrated stability in hepatic microsomes, adequate tissue distribution properties and excellent pharmacokinetic properties. In vivo studies were promising and revealed that this molecule has great potential for a clinical candidate for the treatment of hyperuricemia and gout [59].

**Figure 11:**
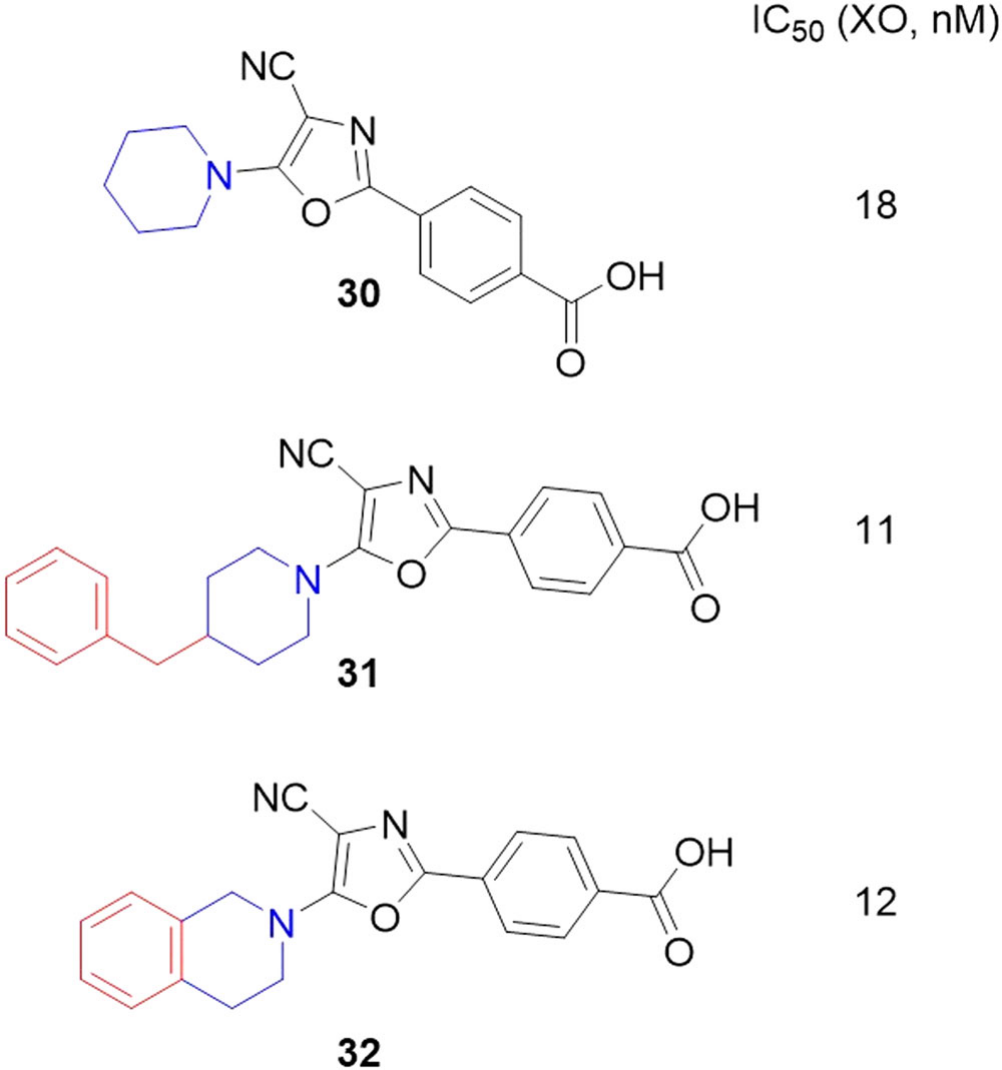
Structural differences between compounds 30–32, where compound 31 was the most potent in the study.

Compounds based on 4‐(5‐aminosubstituted‐4‐cyanooxazol‐2‐yl)benzoic acids were developed by Kobzar et al. to be XO inhibitors. The design of this class was based on the drug febuxostat. Three compounds showed IC_50_ values of less than 20 nM, **30**–**32** (Figure 11). The inhibition of XO in the presence of bovine serum albumin was also evaluated, where the IC_50_ values of **30** and **31** showed a slight worsening in activity, while **32** showed a slight improvement in activity. Enzyme kinetics assays revealed that compound **30** showed mixed‐type inhibition when the inhibitor binds preferentially to the free enzyme rather than the enzyme‐substrate complex. Molecular docking studies revealed that hydrogen bonds with the amino acid residues Arg880 and Thr1010 [60].

**Figure 12:**
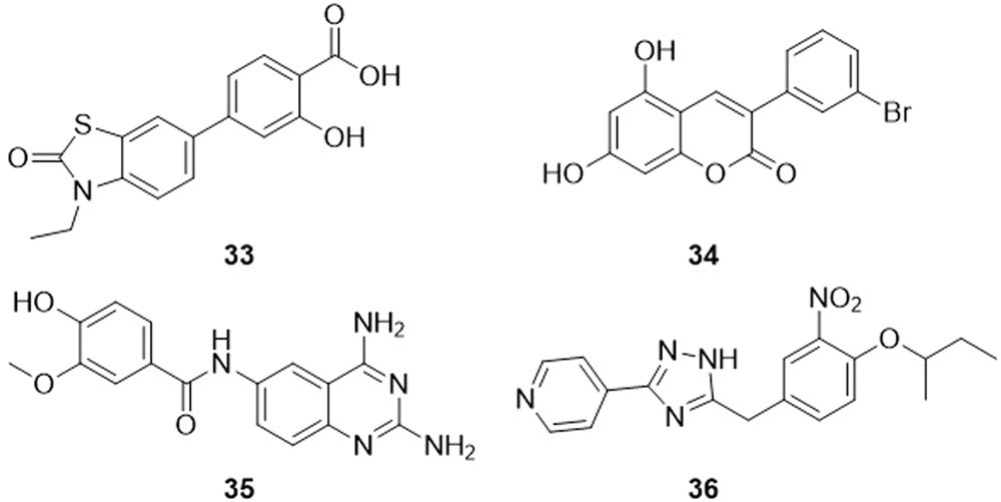
Structures of compounds 33–36 as potential Xanthine oxidase (XO) inhibitors.

Zhu et al. synthesized compounds containing benzoic acid group as new XO inhibitors. Compound **33** exhibited XO inhibitory activity similar to that of febuxostat, with IC_50_ values of 12 and 10 nM respectively (Figure 12). When compared to the other analogs in the study, the one that showed the best activity has a 3‐hydroxy group next to the 4‐carboxyl, which may have influenced the increase in potency, when compared to the other compounds in the study which were more than 27 times less potent (when compared to the second most potent in the study, the others being even less potent). **33** also showed activity for URAT1, although no better than the standard drug, benzbromarone. Molecular docking studies revealed hydrogen bonding interactions with the amino acid residues Arg880, Lys771, and Thr1010. Compound **33** has the potential to be further investigated in in vivo studies, in addition to safety studies that need to be evaluated [61].

A study of 3‐phenylcoumarins as XO inhibitors was carried out by Fais et al. The standout compound in the study, **34**, showed an IC_50_ value of 91 nM for XO inhibition, compared to febuxostat which showed an IC_50_ of 20 nM (Figure 12). Enzyme kinetics assays proved that compound **34** acts as a mixed‐type inhibitor. In addition, antioxidant activity was evaluated, but **34** did not exhibit the best activity among the compounds in the study and, within this, no compound showed a better EC_50_ (half maximal effective concentration) than Trolox. Studies on the Caco‐2 (human colon adenocarcinoma cell line) cell line proved that the study compounds are not cytotoxic. It is worth noting that the compounds were tested in vitro using the Caco‐2 cell line and did not induce cell damage or death, which suggests a favorable preliminary safety profile and supports their potential for future drug development [62].

Lopez‐Sanchez et al. synthesized quinazolin‐2,4,6‐triamine derivatives as non‐purine XO inhibitors. The standout compound in the study, **35**, exhibited an IC_50_ of 1.56 µM when compared to the standard control, allopurinol, with an IC_50_ of 6.99 µM (Figure 12). In addition, a superoxide elimination test was carried out, where none of the compounds showed as good an elimination percentage as allopurinol. The compounds in the study did not induce genotoxicity in a micronucleus model compared to cisplatin or hexavalent chromium (CrO_3_), meaning that they could become a good starting point for the development of new cytotoxic agents or XO inhibitors that are safer [63].

Li et al. evaluated 5‐benzyl‐3‐pyridyl‐1*H*‐1,2,4‐triazole derivatives as possible XO inhibitors. The most promising compound in the study, **36**, exhibited inhibitory activity against XO with an IC_50_ value of 0.16 µM, compared to topiroxostat which showed an IC_50_ of 0.015 µM, not as potent, but demonstrating that the structural skeleton can be considered an acceptable framework for XO inhibition (Figure 12). Molecular docking studies revealed that **36** formed a hydrogen bond with the Glu1261 residue, however, a crucial interaction through hydrogen bonding with the Arg768 residue present in topiroxostat, was absent in **36**, and this may be one of the reasons for the decrease in inhibitory activity in relation to the drug [64].

**Figure 13:**
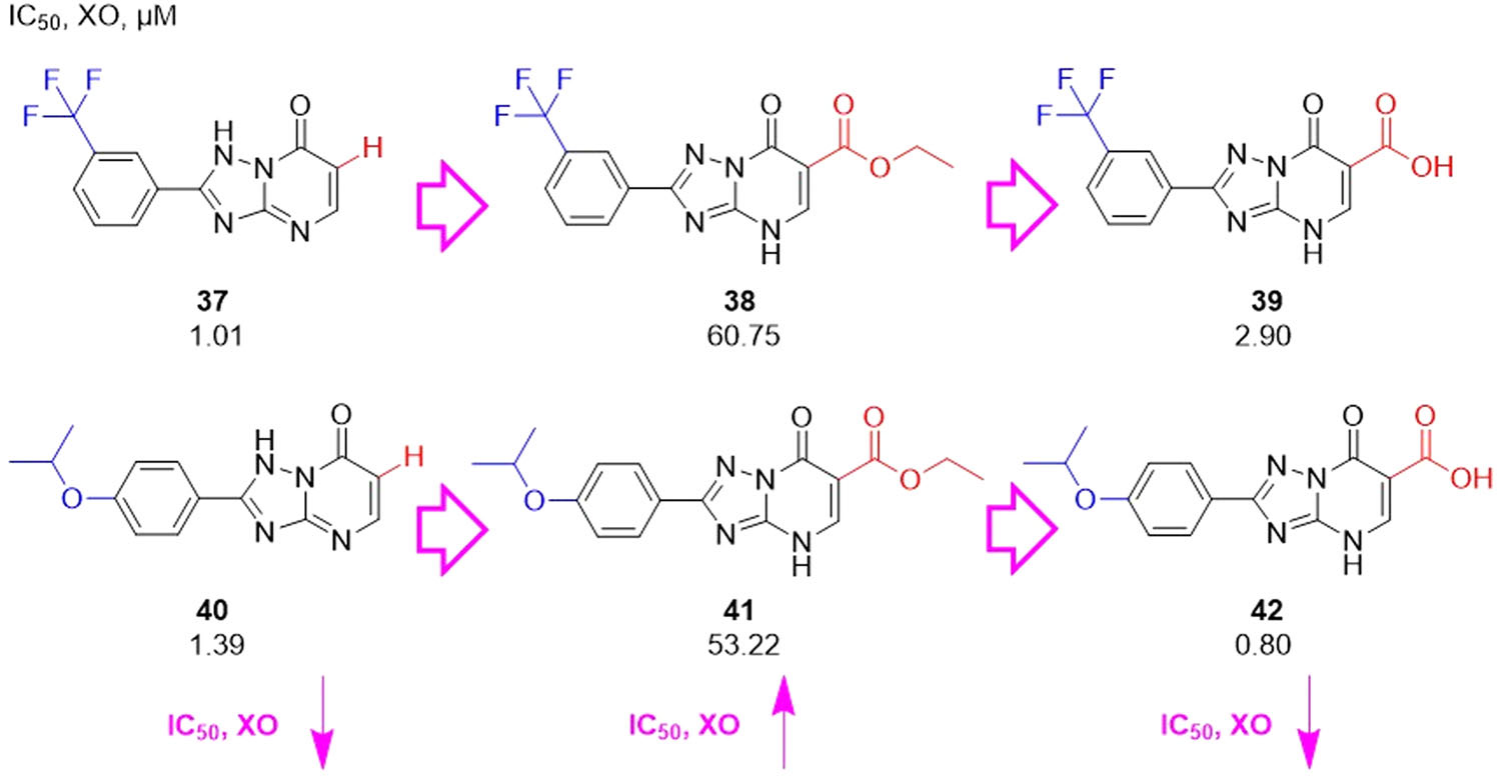
Structural comparison between compounds **37**/**38**/**39** and **40**/**41**/**42**, which have the substituents ─H, ─COOC_2_H_5_, and ─COOH in position six of the purine analogs.

Luna et al. synthesized a series of purine analogs as potential candidates for antihyperuricemic drugs. The structural modifications of the analogs with the best XO inhibition values, with satisfactory IC_50_ values, with changes of the groups in 3‐CF_3_C_6_H_4_ and 4‐iPrOC_6_H_4_, and variations in the same position, 6, with the ─H, ─COOC_2_H_5_, and **─**COOH groups (Figure 13). The intermediates, **38** and **41**, showed no activity (IC_50_ > 50 μM). The best IC_50_ values in the study were compounds **37** and **42**. Enzyme kinetics studies revealed that both compounds are mixed‐type inhibitors. Molecular docking studies revealed hydrogen bonds with the amino acid residues Thr1010, Arg880 and Asn768 for **37** and for **42** the residues Glu802, Lys771, Val1011, and Thr1010. The isopropoxy group showed better XO inhibition activity, while the trifluoromethyl group also showed good activity [65].

**Figure 14:**
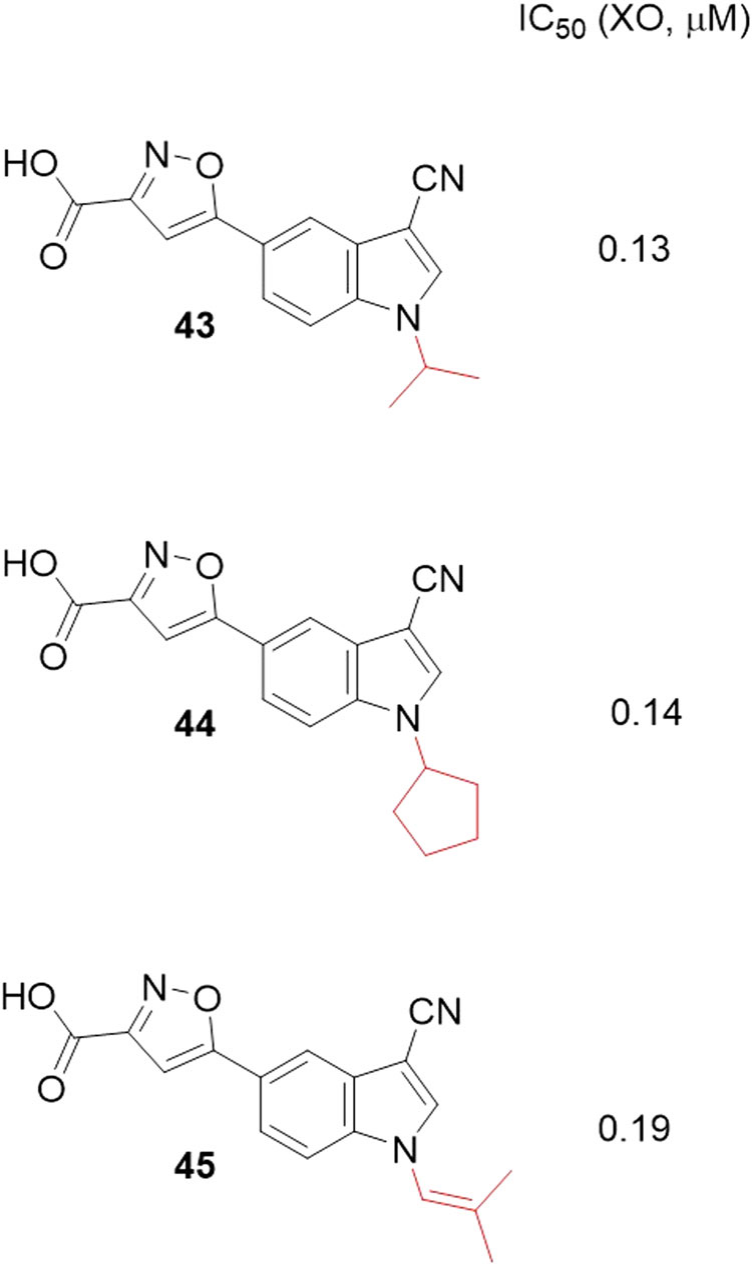
Compound **43** showed the best IC_50_, with the 1‐isopropyl group, when compared to the others, **44** and **45**.

A series of compounds with the *N*‐5‐(1H‐indol‐5‐yl)isoxazole‐3‐carboxylic acids skeleton were evaluated as new XO inhibitors. Huang and colleagues discovered a compound, **43**, with potent inhibitory activity, exhibiting an IC_50_ value of 0.13 µM, against the drug allopurinol and febuxostat, with IC_50_ values of 2.93 and 0.01 µM, respectively. Figure 14 shows the four compounds with the best inhibitory activities, ranging from 0.13 to 0.19 µM, with changes only in the indolic ring, in position one (**43**–**45**). These compounds were prepared using an isosteric/bioisosteric substitution strategy. structure–activity relationship studies showed that the 3‐cyano group was indispensable for these compounds to maintain their XO inhibitory potency. Compound **43** acts as a mixed‐type inhibitor. In molecular docking studies, **43** interacts by making two hydrogen bonds with the amino acid residues Asn768 and Lys771, and the carboxyl group of the isoxazole ring could form multiple hydrogen bonds with Arg880 and Thr1010. **43** has the potential for further optimization to become a drug candidate for the treatment of hyperuricemia or gout [66].

**Figure 15:**
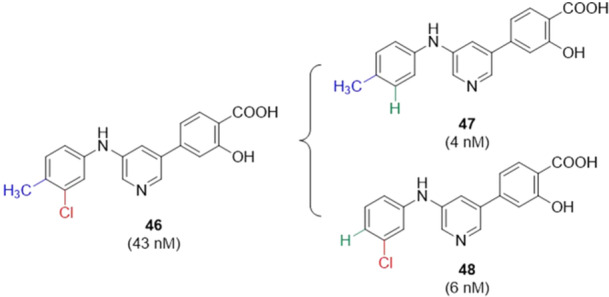
Compound **46** has 3‐chloro‐4‐methyl groups, while compounds **47** and **48** have only 4‐methyl and 3‐chloro, respectively, all in relation to the amino group. The exchange of a hydrogen in **47** and **48** improved the inhibitory potency of XO.

Yang and collaborators synthesized 3‐phenyl substituted pyridine derivatives as dual inhibitors of XO and URAT1. **46** showed interesting activity for XO (IC_50_ = 0.043 µM) with two substituents on the benzene ring, 3‐chloro and 4‐methyl, however, when one of the substituents was removed, generating **47** and **48**, with the 4‐methyl and 3‐chloro groups, respectively, the XO inhibitory activity improved more than tenfold for **47** and in the case of **48** it improved more than sevenfold, being more potent than febuxostat itself, which showed an IC_50_ value of 0.008 µM (Figure 15). However, when we looked at the inhibitory activity of URAT1, compounds **46** and **47** did not show a better IC_50_ than the drug benzbromarone, while **48** showed an IC_50_ of 12.90 µM, while the drug showed 27.04 µM, twice as potent as the standard drug used in the study. In vivo studies in mice with acute hyperuricemia have shown that **47** and **48** can significantly decrease serum uric acid levels at a dosage of 5 mg.kg^−1^. In any case, febuxostat showed a better effect when compared to the compounds tested, but **48** had an almost similar effect to the drug febuxostat. **47** and **48** are possible candidates for XO inhibitors, while **48** would be a possible candidate for URAT1 inhibitors. Further studies can be conducted to optimize the structures and make them potential drug candidates [67].

**Figure 16:**
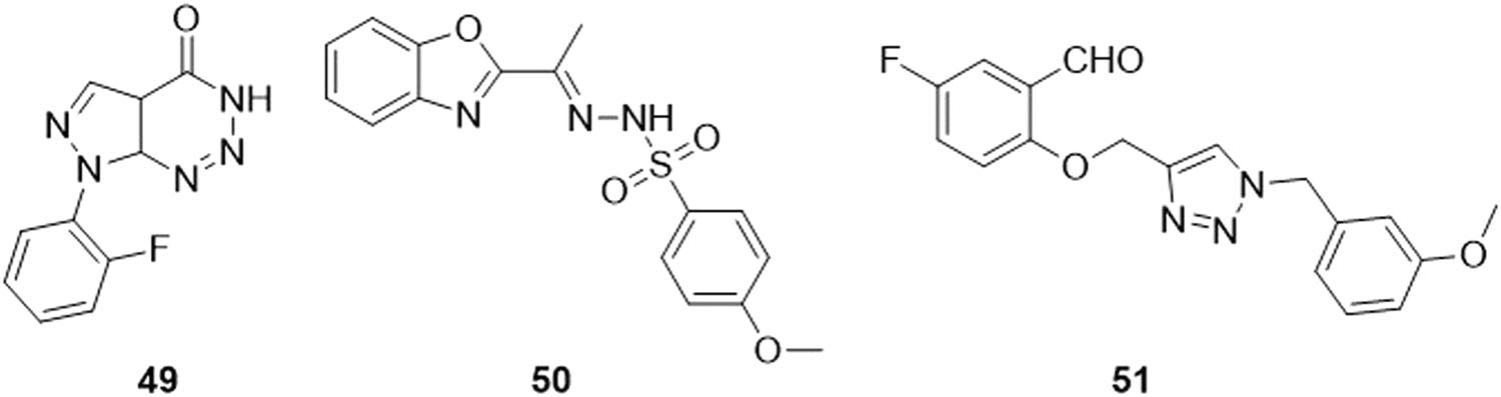
Structures of compounds **49**–**51** as potential XO inhibitors.

Sciú et al. identified pyrazolotriazinones as potential agents for the treatment of hyperuricemia. Although this group of compounds was no more active than the drug allopurinol, which had an IC_50_ of 0.247 µM, the most active compound in the study had an IC_50_ of 0.907 µM (Figure 16). Molecular docking studies revealed that **49** made a hydrogen bond with the Glu802 residue, considered an essential interaction for the catalytic reaction. This series of purine analogs could be used for further evaluations to obtain new therapeutic agents for the treatment of gout [68].

An interesting study investigated compounds as inhibitors of XO and Nod‐like protein receptor 3 (NLRP3). One of the compounds synthesized by Wang et al. **50**, exhibited good inhibitory effects on NLRP3 and XO (Figure 16). **50** showed an IC_50_ of 4.2 and 7.5 µM, for XO and NLRP3, respectively, and was no more potent than the drug used as a standard, allopurinol (2.2 µM) for XO, but more potent than isoliquiritigenin (10.1 µM, IL‐1β). This compound proves to be a dual‐target XO‐NLRP3 inhibitor, which can reduce uric acid and relieve inflammation, and could be an interesting and possibly promising approach for the treatment of gout [69].

Zhang et al. carried out studies with 4‐(phenoxymethyl)‐1*H*‐1,2,3‐triazole derivatives to obtain new inhibitors of the XO enzyme. Compound **51** stood out, showing an IC_50_ of 0.70 µM, compared to the standard control allopurinol, with an IC_50_ of 9.80 µM (Figure 16). When subjected to molecular docking tests to visualize interactions, it was possible to observe hydrogen bonds with residues Thr1010 and Val1011, and consequently there was a decrease in the bond distance. In addition, the **51** molecule underwent in vitro evaluations for hyperuricemic effects with hyperuricemic rats, with allopurinol as the reference, and the conclusion of the study showed that compared to the model group, the **51** compound showed a 31% reduction in uric acid levels [70].

Raghu and colleagues synthesized compounds derived from pyrazolo[1,5‐*a*] [1, 3, 5] triazine as antioxidant agents and possible inhibitors of lipoxygenase and XO. The compounds with the best biological activities are described in Table 2 and when compared to the positive control's ascorbic acid (IC_50_ = 92.63 µM), trolox (IC_50_ = 96.38 µM) and allopurinol (IC_50_ = 34.08 µM), **52**–**54** showed higher values. **52** showed the best activity in all the studies. It is important to note that as phenolic hydroxyls were added, the biological activities improved slightly [71].

**Table 2:** Structural comparison between compounds 52–54 with their respective DPPH (2,2‐diphenyl‐1‐picrylhydrazyl), FRAP (ferric reducing antioxidant power), LOX (lipoxygenase) and XO IC_50_ values.

		IC50 (µM)			
Compound	Structure	DPPH	FRAP	LOX	XO
**52**	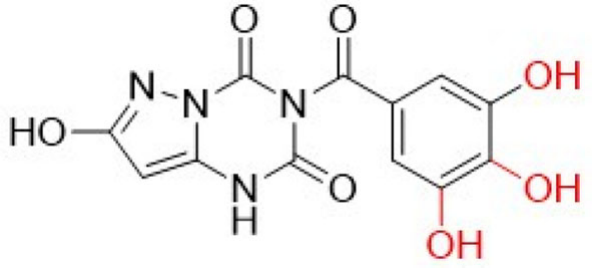	80.33	85.69	16.85	23.01
**53**	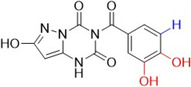	84.65	91.52	18.32	25.69
**54**	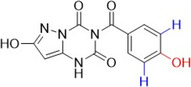	88.21	93.16	22.81	28.55

**Figure 17:**
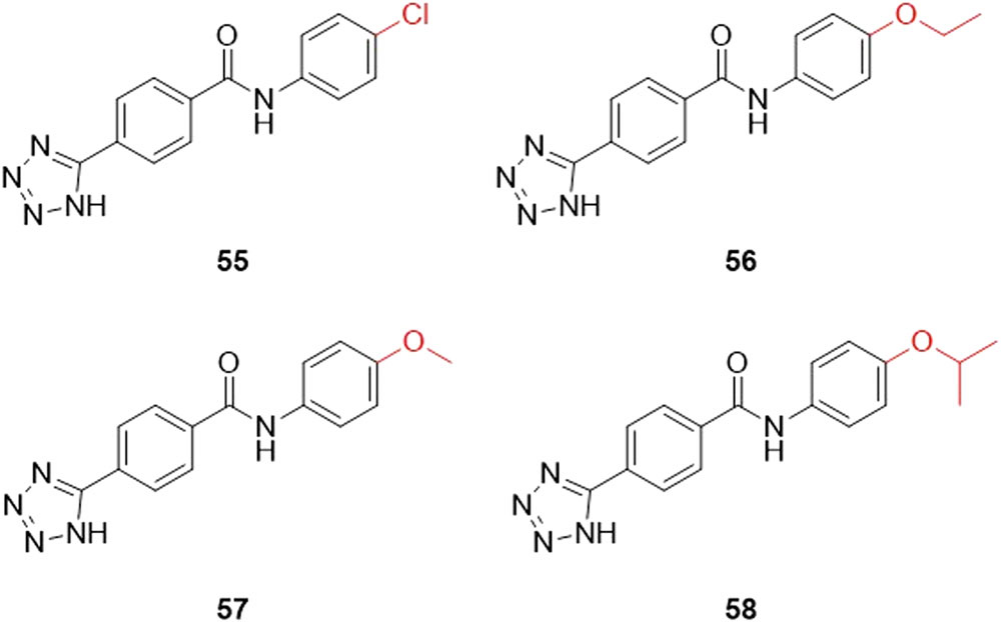
Comparison between the para‐position modifications, with compound **55** having the greatest inhibitory activity against XO.

Peng and collaborators designed tricyclic compounds containing phenyl‐tetrazole that exhibited inhibitory activity against XO. The compounds were designed by replacing the carboxyl‐thiazole fragment of the drug febuxostat with the tetrazole ring, using the bioelectronic isosteric strategy. Four compounds, **55**–**58**, showed IC_50_ values in the range of 26.30 to 55.00 nM, with **55** being the most active in the study, with the 4‐Cl substituent, and relatively comparable to the positive control, febuxostat, with an IC_50_ of 6.91 nM (Figure 17). The molecular docking study showed that **55** and **56** made a hydrogen bond with the Arg880 residue via the tetrazole ring, as well as another hydrogen bond with Ser876. In vivo studies using a potassium oxonate/hypoxanthine‐induced acute hyperuricemia model showed a significant hyperuricemia effect of compounds **55**, **56,** and **58**, consistent with the in vitro inhibition results, but they did not show uric acid‐reducing activity as good as that of the drug febuxostat [72].

**Figure 18:**
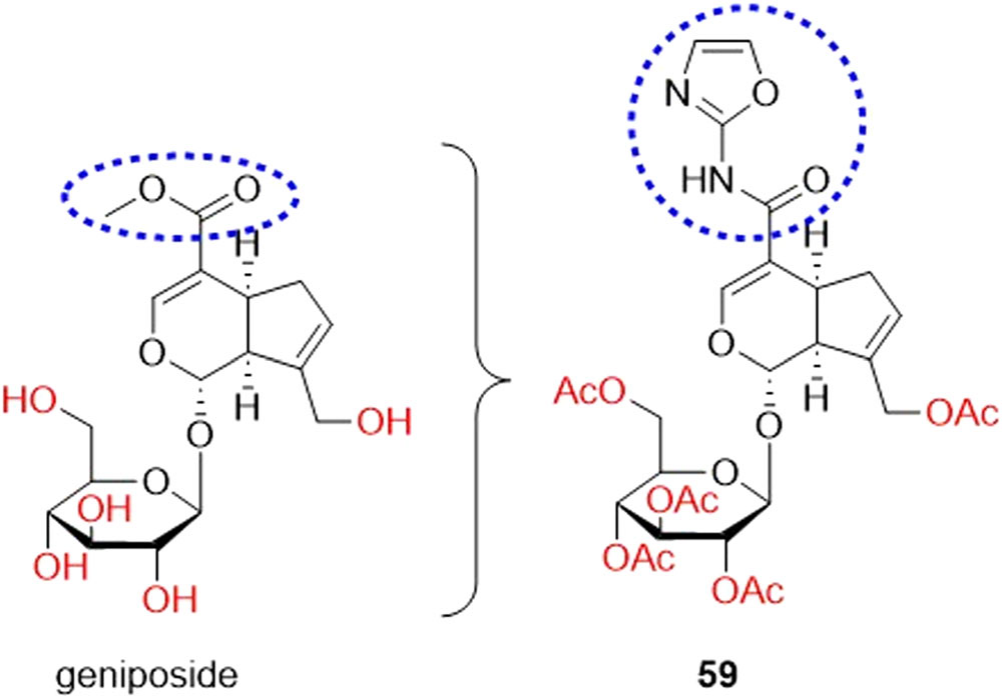
Relationship between geniposide and **59**, showing the difference in hydroxyls and the ester portion, which has been acetylated and replaced by a ─NH‐oxazol‐2‐yl, increasing its inhibitory potency against xanthine oxidase (XO).

Chen and collaborators synthesized geniposide derivatives as inhibitors of hyperuricemia, inflammation and fibrosis. Compound **59** was the most potent XO inhibitor in vitro, with an IC_50_ value of 1.37 µM, being 117.81 times more active than geniposide and 9.14 times more active than the drug allopurinol (Figure 18). Enzyme kinetics studies revealed that **59** was considered a mixed‐type XO inhibitor. It was identified that **59** can regulate the TLR4/IκBα/NF‐κB signaling pathway, reducing the level of inflammatory factors, as well as being able to inhibit renal fibrosis by reducing the expression of TGF‐β (transforming growth factor beta). In vivo studies showed that **59** exhibited the strongest anti‐hyperuricemia and renal protective activity, effectively lowering serum uric acid levels in mice with hyperuricemia by inhibiting XO activity and also effectively reducing urate accumulation in the kidney. Molecular docking studies revealed that compound **59** forms five hydrogen bonds with the amino acids Ala338, Asn351, Arg426, and Lys433 [73].

Güzel et al. synthesized benzimidazolium salts containing the 4‐(methylsulfonyl)benzyl group as possible inhibitors of XO and AChE (acetylcholinesterase). The compounds showed inhibitory activities against XO, with IC_50_ values ranging from 0.218 to 1.917 µM, while for AChE, with IC_50_ values ranging from 1.328 to 5.22 µM (Table 3). **60**–**62** showed the best inhibitory properties against both enzymes and were more active than the positive controls themselves, allopurinol (1.56 µM) and donepezil (3.417 µM). Molecular docking studies revealed that **60** showed greater affinity than the positive control, forming a hydrogen bond with the Arg880 residue of XO [74].

**Table 3:** Structural comparison between compounds 60–62 with their respective XO and AChE IC_50_ values.

		IC_50_ (µM)	
Compound	Structure	XO	AChE
**60**	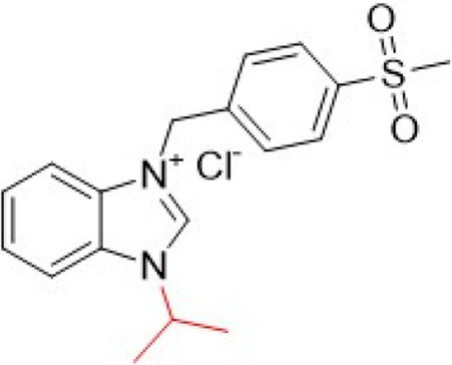	0.218	1.41
**61**	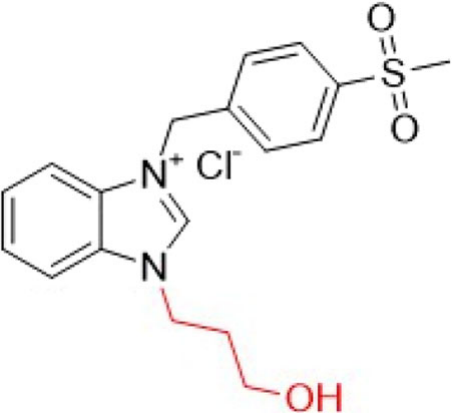	0.463	1.49
**62**	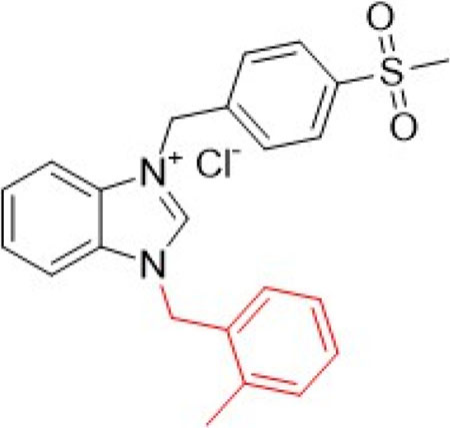	0.545	1.328

Abbreviations: AChE, acetylcholinesterase; XO, xanthine oxidase.

**Figure 19:**
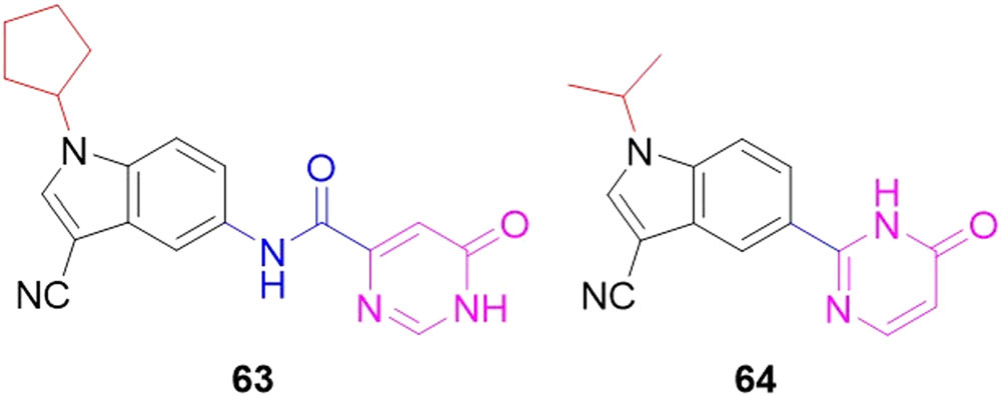
Structural variation between compounds **63** and **64**, with **64** being almost twice as potent against xanthine oxidase (XO).

Zhang et al. designed, synthesized and evaluated two types of pyrimidone derivatives as potential XO inhibitors. The compounds, **63** and **64**, showed IC_50_ values of 0.16 and 0.085 µM, respectively (Figure 19). **63** was more than 52 times more potent than the drug allopurinol (8.37 µM). For compound **64**, which replaced the intermediate amide fragment with a single bond between the pyrimidone and indole rings, **64** was more than 98 times more potent than allopurinol. It was confirmed that hydrophobic groups in the N‐indol position were indispensable for improving in vitro inhibitory potency against XO. Enzyme kinetics studies suggested that compounds **63** and **64** acted as mixed‐type inhibitors. In addition, molecular docking studies revealed that both compounds, with the pyrimidone and indole fractions, could occupy the active pockets of XO and interact well with key amino acid residues. Finally, in vivo studies were conducted which showed that **63** and **64** can reduce serum uric acid levels at an oral dose of 10 mg.kg^−1^. These compounds could be possible agents for treating hyperuricemia and gout [75].

**Figure 20:**
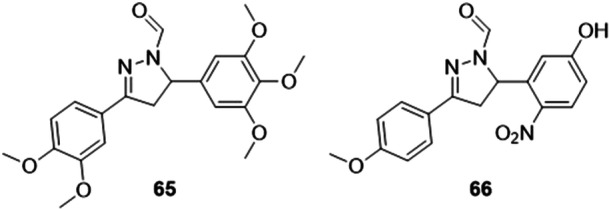
Structures of compounds **65** and **66** as possible xanthine oxidase (XO) inhibitors.

Joshi et al. studied 3,5‐diaryl‐4,5‐dihydro‐1*H*‐pyrazole carbaldehydes as non‐purine XO inhibitors. The compounds that were the highlight of the study, **65** and **66**, inhibited the growth of XO‐bearing cancer cells in both 2D and 3D tissue, such as an MDA‐MD‐231 cell model (Figure 20). The IC_50_ values for XO inhibition were 9.32 and 10.03 µM respectively, being more active than allopurinol (13.03 µM). For **66**, the results were interesting. Both compounds showed apoptosis as cell death and induced cell cycle arrest in the G1 phase. Apoptosis was mediated by reactive oxygen species (ROS) in cancer cells due to increased oxidative stress, altered mitochondrial membrane permeability (MMP) and inhibition of antioxidant enzymes (superoxide dismutase and glutathione reductase) caused by compounds **65** and **66**. In addition, miRNA levels and the expression of redox sensors were examined in cells altered due to the increased oxidative stress induced by the compounds studied. In vivo studies will be conducted for further investigation [76].

**Figure 21:**
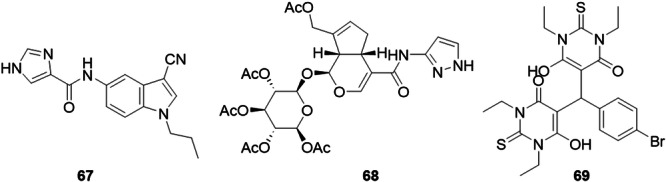
Structures of compounds **67**–**69** as potential xanthine oxidase (XO) inhibitors.

Zhang et al. developed analogs of isonicotinamide in their previous work as a possible XO inhibitor. They chose to synthesize new molecules derived from *N*‐(1‐alkyl‐3‐cyano‐1*H*‐indol‐5‐yl) to repair the missing hydrogen bond interaction between the 3′‐cyano residue and Asn768 of XO, shortening its distance, which through molecular docking and molecular dynamics studies proved its presence. The outstanding compound, **67**, had an IC_50_ value of 0.018 µM and was comparable to topiroxostat, which had an IC_50_ of 0.013 µM (Figure 21). Through in vivo tests, oral administration indicated an apparent reduction in serum uric acid levels in a rat model of acute hyperuricemia. The MTT results indicated that the compound is not toxic to healthy cells, and the liver microsomal stability assay illustrated that it has good metabolic stability in rat liver microsomes. However, the in vivo potency was lower than that of topiroxostat, which could be explained by the poor absorption found in the parallel artificial membrane permeability assay [77]. Wang and collaborators synthesized a series of geniposide derivatives with the aim of finding new inhibitors for XO, varying the scope by adding alicyclic, aromatic, aromatic heterocycle and alicyclic substituents, which resulted in activities ranging from 6.67 to 128.76 µM. In vitro studies showed that the electron‐withdrawing substituents had greater activity against the XO enzyme. Among the compounds synthesized, **68** stood out due to its greater inhibitory activity with an IC_50_ value of 6.67 µM (Figure 21). With this compound in mind, in vivo tests were carried out on rats with induced hyperuricemia, in which the efficiency of compound **68** in combating hyperuricemia was observed, an efficiency that can be compared to allopurinol, a well‐established drug for the treatment of hyperuricemia. It was also possible to observe an increase in the viability of HKC cells, a reduction in the concentration of uric acid, creatinine and urea nitrogen in the blood of the rats treated with compound **68**, which demonstrates efficiency not only in the treatment of hyperuricemia, but also in its side effects on the renal system of the rats tested. In conclusion, the study proposes a new drug candidate for the treatment of hyperuricemia, which shows good results for this treatment [78].

Serrano et al. designed and synthesized a series of bis‐thiobarbiturates to find good inhibitors for XO, due to the influence of barbiturates and thiobarbiturates on neurotransmission. XO inhibition studies were carried out using spectrophotometric quantification of uric acid formation. In these studies, those with electron withdrawing groups in the para position of the phenyl fraction were highlighted as having the greatest activity, in particular compound **69**, containing bromine in this position, showed the best inhibitory activity against XO (IC_50_ = 1.79 µM), almost 10 times more effective than allopurinol (IC_50_ = 10.73 µM) (Figure 21). A cytotoxicity test was carried out on nontumor human dermal fibroblasts (NHDF) cells, demonstrating its low cytotoxicity. The in silico properties were met for the test compound, which could be a possible candidate for the treatment of hyperuricemia, and further research into the molecule should be carried out [79].

**Figure 22:**
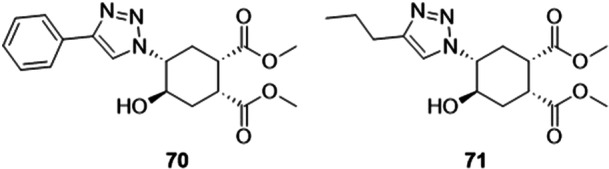
Structures of compounds **70** and **71** with only one structural difference, a phenyl group for an isopropyl group linked to the 1,2,3‐triazole.

A new series of 1,2,3‐triazole compounds was investigated as potential XO inhibitors. Tan obtained two compounds, **70** and **71**, which showed better inhibitory activity than the drug allopurinol, used as a positive control. The IC_50_ values were 0.586, 0.751, and 1.143 µM respectively, demonstrating the potential activity of the triazoles (Figure 22). Only compound **71** showed hydrogen bonding with the Ser876 residue. The binding energy values for both compounds were better, when compared to allopurinol, but not so far apart (−6.7 and −6.2, for **70** and **71**, and allopurinol, −6.1), as other interactions may have stabilized the complexes between the compounds and the XO binding site. Further in vivo studies may corroborate the data obtained in vitro [80].

### 3.3 Four Cycles

**Figure 23:**
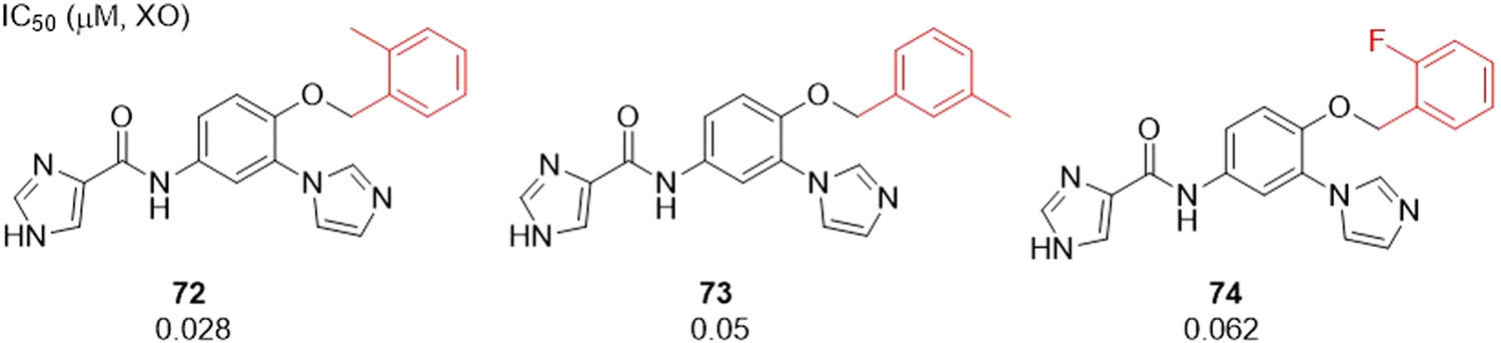
Comparison between compounds **72**–**74**, with **72** showing the best inhibitory activity against xanthine oxidase (XO).

Hu et al. investigated *N*‐phenyl aromatic amides derivatives as potent XO inhibitors. Three compounds showed an IC_50_ equal to or lower than 62 nM, **72**–**74**, demonstrating satisfactory activity, and even more so for the most potent amide, with an IC_50_ of 28 nM. The positive control used was topiroxostat, which showed an IC_50_ of 17 nM, very close to compound **72**, the most active compound in the study (Figure 23). **72** was shown to be a mixed‐type XO inhibitor. Molecular docking studies revealed that **72** makes hydrogen bonds with the residues Glu1261, Glu802 and Asn768. Results using MTT indicated that **72** was not toxic against healthy cells. In addition, **72** showed an apparent hypouricemic effect in a rat model of acute hyperuricemia, and the uric acid‐lowering effect was improved when compared to compounds previously investigated by the same group. Further research could lead to compound **72** being considered as a possible drug candidate [81].

**Figure 24:**
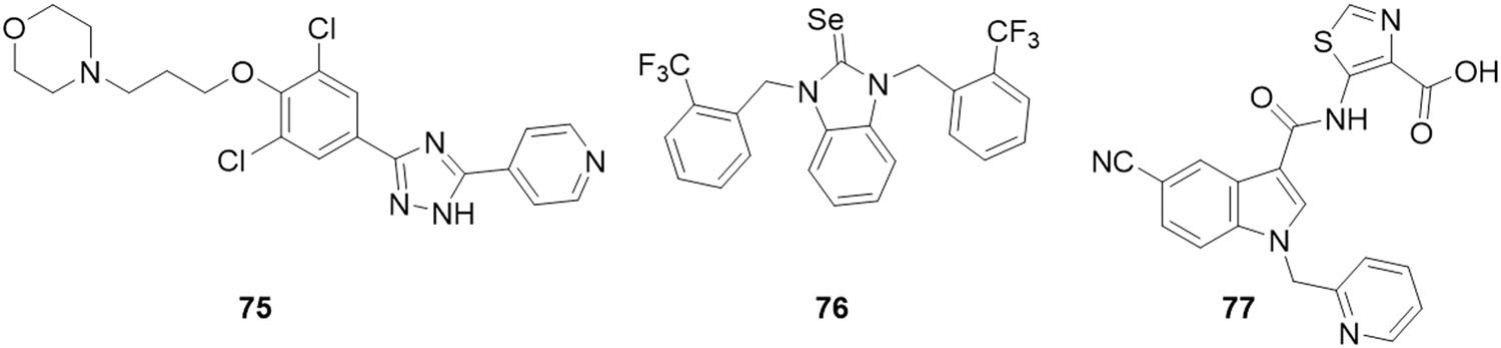
Structures of compounds **75**–**77** as potential xanthine oxidase (XO) inhibitors.

A series of 1,2,4‐triazole derivatives have been discovered as potent XO inhibitors. Yang et al. designed a series of triazoles with a fused pharmacophore strategy based on the drugs febuxostat and topiroxostat. The study's lead compound, **75**, showed satisfactory inhibition against XO with an IC_50_ value of 0.20 nM, which was higher than febuxostat and topiroxostat (Figure 24). Molecular docking studies revealed that **75** made hydrogen bonds with residues Glu802, Leu648, and Pro1076, and when compared to the interaction modes of the drugs used as positive controls in the in vitro tests, **75** preserved most of the important interactions involved. In vivo studies demonstrated that **75** exhibited significant hyperuricemic and serum XO inhibitory effects in mouse models of potassium oxonate‐induced hyperuricemia. In addition, the evaluation of the toxicity of a single dose of **75** showed no noticeable toxicity at a dose of 50 mg.kg^−1^. All these results show that **75** could be a promising candidate for the treatment of hyperuricemia and gout [82].

Li et al. investigated a small molecule, **75** (CC15009) as a specific inhibitor of XO, of the non‐purine type, synthesized previously by Yang et al. [82]. This compound showed an IC_50_ of 0.237 nM and was extremely potent, being able to act as a competitive or reversible inhibitor, having a more satisfactory activity in the order of five times that of allopurinol, and being approximately 15 times more potent than febuxostat. In addition, in vivo studies have shown that **75** has been identified as a possible XO inhibitor (Figure 24). The study showed that single administration of N significantly inhibited serum XO activity in hypoxanthine‐induced mice and in chronic yeast‐induced mice. The compound exerted uric acid‐lowering activity, which was far superior to allopurinol at the same dosage in hypoxanthine‐induced mice. Another assay developed showed that uric acid uptake applied to URAT1 overexpressing cells also indicated that **75** did not affect the transport activity of URAT1, the main target of current uricosuric drugs, revealing that **75** acted as a specific XO inhibitor with no direct effect on uric acid excretion. **75** proved to be safe in relation to the concerns regarding current XO inhibitors. This molecule appears to be a promising candidate to be improved and developed to treat hyperuricemia [83].

Kaya et al. designed XO and acetylcholinesterase inhibitors with the selenium‐based *N*‐heterocyclic carbene skeleton. Compound **76** showed the best activity for XO and AChE, with IC_50_ values of 0.361 and 0.995 µM, respectively, both of which outperformed the standard drugs used as a positive control, allopurinol and donepezil (Figure 24). All the compounds in the study had IC_50_ values lower than allopurinol for XO inhibition and also than donepezil for AChE inhibition. DNA binding studies were carried out and the compounds did not exhibit DNA binding properties. Molecular docking studies showed that **76** had the best binding energy and showed hydrogen bonding interactions with residues Arg880 and Phe1009 of XO [84].

Lin et al. identified an interesting molecule for a dual inhibitor of XO and URAT1. The compound **77**, 5‐[5‐cyano‐1‐(pyridin‐2‐ylmethyl)‐1*H*‐indole‐3‐carboxamido] thiazole‐4‐carboxylic acid, showed activity with an interesting potency for further investigation, on URAT1 (48.0% at 10 µM) and on XO (1.01 µM), exhibiting better activities than the standard controls used in the study (Figure 24). Molecular docking studies revealed that **77** makes hydrogen bonds with residues Arg880, Thr1010 and Val1011. In addition, in vivo studies were conducted which showed that compound **77** could reduce serum uric acid levels at a dose of 10 mg.kg^−1^ [85].

An interesting study by Yavuz et al. investigated thioether‐substituted benzimidazolium salts as inhibitors of XO and AChE. Compounds **78**, **79**, and **80** showed IC_50_ values of less than 0.6 µM for XO inhibitory activity and for AChE, the IC_50_ values showed the same trend, with values of less than 1.0 µM (Table 4). It is interesting to note that **78**, the most active compound in the study, has a larger 4‐ethenyl group compared to **79** and **80**, which have a methyl group in positions 4 and 3, respectively. In the crystal structure, the molecules are connected by intra‐ and intermolecular hydrogen bonds C─H···Br. These benzimidazolium salts showed better biological activities when compared to the positive controls, allopurinol and donepezil, and all the compounds in the study showed more satisfactory IC_50_ values than the drugs themselves. It is important to note that compounds **78**–**80** exhibited the best therapeutic profiles for both targets compared to the others and could be interesting to investigate beyond what has already been invested [86].

**Table 4:** Structural comparison between compounds 78–80 with their respective XO and AChE IC_50_ values.

		IC_50_ (µM)	
Compound	Structure	XO	AChE
**78**	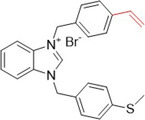	0.548	0.813
**79**	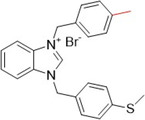	0.575	0.931
**80**	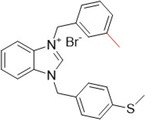	0.583	0.965

Abbreviations: AChE, acetylcholinesterase; XO, xanthine oxidase.

**Figure 25:**
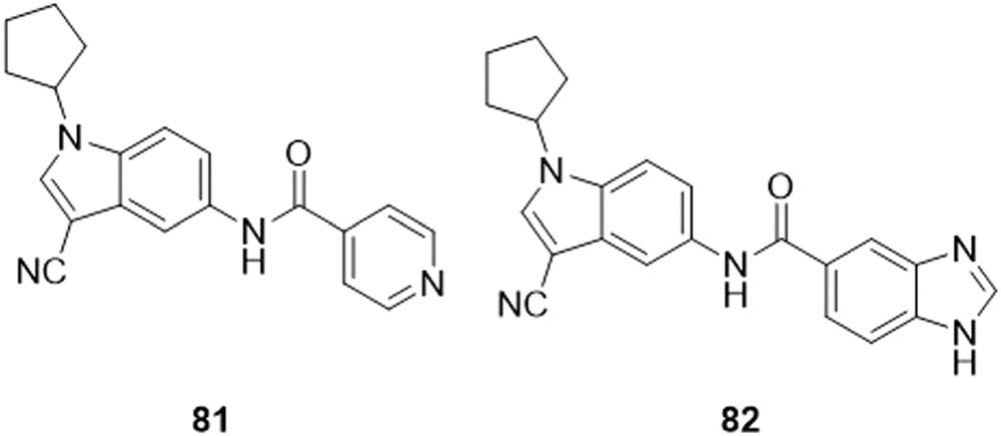
The two amide‐based compounds, **81** and **82**, with the best inhibitory activity against xanthine oxidase (XO).

Tu et al. proposed two series of amide‐based inhibitors of the XO enzyme, because the introduction of fused aromatic rings (such as indole) on one or both sides of the amide allows the hydrogen bond interactions with the residues to be balanced and contributes to the binding affinity, thereby enhancing the inhibitory potency. Subsequently, molecular docking verified this idea and noted the balance between the residues, the hydrogen bonds and the synthesized compound. Two compounds stood out among the rest, showing an IC_50_ around 14 times more potent than allopurinol, despite being lower when compared to topiroxostat, these being molecules **81** and **82** with respective IC_50_ values of 0.73 and 0.62 μM (Figure 25). Along with this, other significant properties were shown by compound **82**, namely not exhibiting toxicity after high doses and high human intestinal permeability, which proved to be better than allopurinol [87].

**Figure 26:**
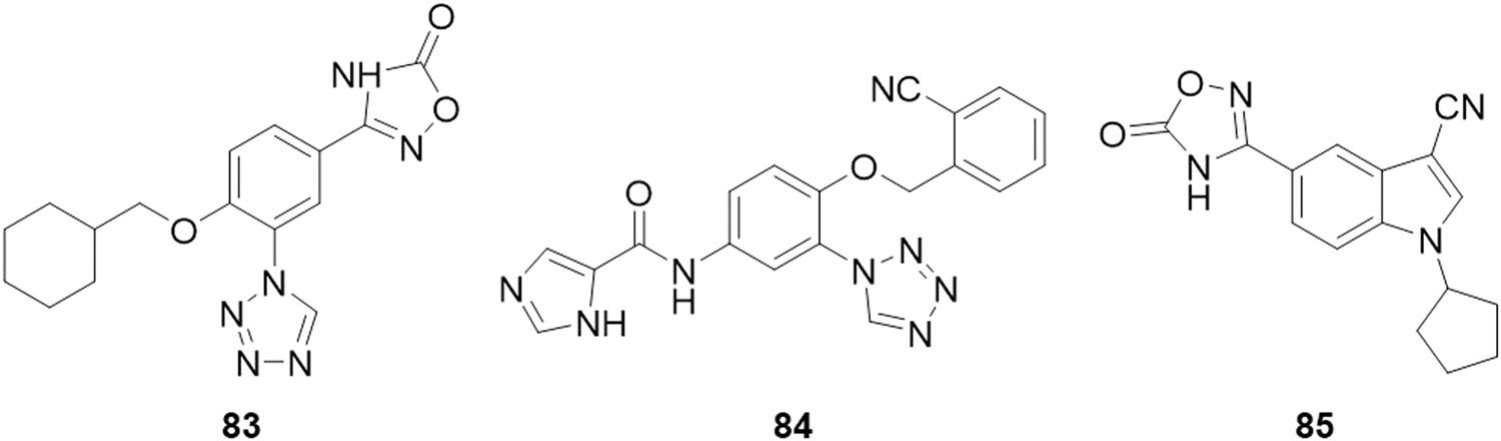
Structures of compounds **83**–**85** as potential xanthine oxidase (XO) inhibitors.

Gao et al. synthesized new molecules with the 3‐[4‐alkoxy‐3‐(1*H*‐tetrazol‐1‐yl)phenyl]‐1,2,4‐oxadiazol‐5(4*H*)‐ones skeleton as possible XO inhibitors for the treatment of hyperuricemia and gout. The lead compound, **83**, proved to be a mixed‐type non‐purine XO inhibitor, and through in vitro inhibition of bovine XO, the compound showed IC_50_ values of 0.121 µM, which was approximately 63 times more potent than allopurinol, the standard drug used as a control (Figure 26). Molecular docking studies show that the 1,2,4‐oxadiazole 5(4*H*)‐one portion binds to the active site of XO through hydrogen bonds, two of which are from the carbonyl group to the guanidine group of Arg880, and the other to the N‐4 atom which interacts with the hydroxyl group of Thr1010. The structure–activity relationship studies showed that the hydrophobic group in the 4' position was essential for its inhibitory potency [88].

Zhang et al. synthesized heterocyclic aromatic amide derivatives *N*‐(4‐alkoxy‐3‐(1*H*‐tetrazol‐1‐yl)phenyl) as potential inhibitors of XO, which is a flavoprotein that can catalyze the formation of uric acid. The molecule with the best activity, **84**, proved to be a mixed‐type XO inhibitor, and from in vitro tests, exhibited IC_50_ values of 0.022 µM, equivalent to topiroxostat, the drug used as a positive control (Figure 26). Analysis of the structure activity relationship revealed that replacing the pyridine with a five‐membered imidazole ring can maintain or increase the potency of the inhibition. Molecular docking studies indicated hydrogen bonds in the carbonyl group linked to residues Arg880 and Thr1010, one in the N‐4 atom of the tetrazole with Asn768, and two in the NH portion of the imidazole with Glu1261. The compound also exhibited a hypourecemic effect through in vivo tests on rats with potassium oxonate‐induced hyperuricemia.

The design of 1‐alkyl‐5/6‐(5‐oxo‐4,5‐dihydro‐1,2,4‐oxadiazol‐3‐yl)‐1*H*‐indole‐3‐carbonitriles as new XO inhibitors was evaluated by Gao et al. Compound **85** was the most active in the study, with an IC_50_ value of 0.36 µM, while the drug allopurinol exhibited an IC_50_ of 7.59 µM (Figure 26). structure–activity relationship studies showed that the 5‐oxo‐4,5‐dihydro‐1,2,4‐oxadiazole moiety linked at the 5‐position of the indole was more preferable than the 6‐position for XO inhibitory potency. Kinetic studies revealed that **85** was considered a mixed‐type inhibitor for XO. Results obtained from the molecular docking evaluation showed that the 5‐oxo‐4,5‐dihydro‐1,2,4‐oxadiazole fraction could interact with residues Arg880 and Thr1010 by hydrogen bonds and the cyano group could form hydrogen bonds with residues Asn768 and Lys771. In vivo studies in a rat model of hyperuricemia induced by potassium oxonate suggested that **85** could reduce serum uric acid levels at an oral dose of 10 mg.kg^−1^.

### 3.4 Five or More Cycles

**Figure 27:**
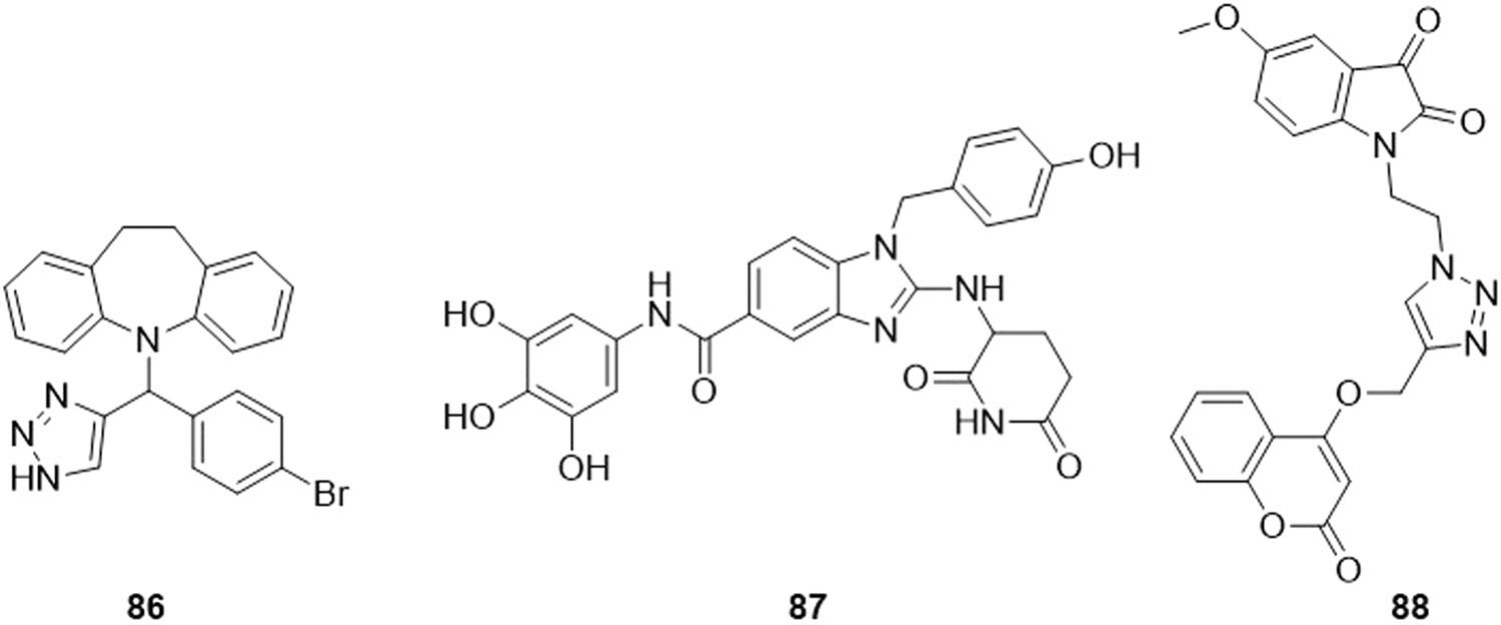
Structures of compounds **86**–**88** as potential xanthine oxidase (XO) inhibitors.

Yadav et al. synthesized new hybrids of 10,11‐dihydro‐5*H*‐dibenzo[*b*,*f*]azepine triazoles as a XO inhibitor. Compound **86** exhibited an IC_50_ of 4.93 μg.mL^−1^, when compared to the positive control, allopurinol, with an IC_50_ equal to 10.29 μg.mL^−1^ (Figure 27). In addition, compound **86** showed antioxidant activities and ferric reducing ability of plasma, with IC_50_ values of 3.58 μg.mL^−1^ and 58.66 μg.mL^−1^, respectively, compared to the positive control, ascorbic acid, with **86** being three times more potent for both activities [89].

Theodore et al. studied the potential of 1*H*‐benzo[*d*]imidazole‐5‐carboxamides as possible XO inhibitors with potential antioxidant activities. The standout compound in the study, **87**, showed an IC_50_ of 18.43 µM for XO inhibition, exhibiting an activity almost twice that of the drug allopurinol (Figure 27). In addition, **87** showed activity against lipoxygenase, DPPH and FRAP, and in all cases was the best evaluated compound, even when compared to positive controls. This molecule has the potential for further research and structural modifications to improve its activity and make it a possible drug candidate [90].

A set of indolinedione‐coumarin hybrids was designed and synthesized for evaluation against hyperuricemia. Gulati et al. found that **88** exhibited the most potent IC_50_ of the compounds in the study, 6.5 µM, and more potent against allopurinol (8.16 µM), used as a positive control (Figure 27). Enzyme kinetics studies revealed that **88** behaved as a mixed‐type inhibitor. structure–activity relationship studies showed that electron‐donating groups and small alkyl chains between the active scaffolds can be beneficial for inhibiting the XO enzyme. **88** forms hydrogen bonds with residues Ala1079, Arg880 and Thr1010 [91].

**Figure 28:**
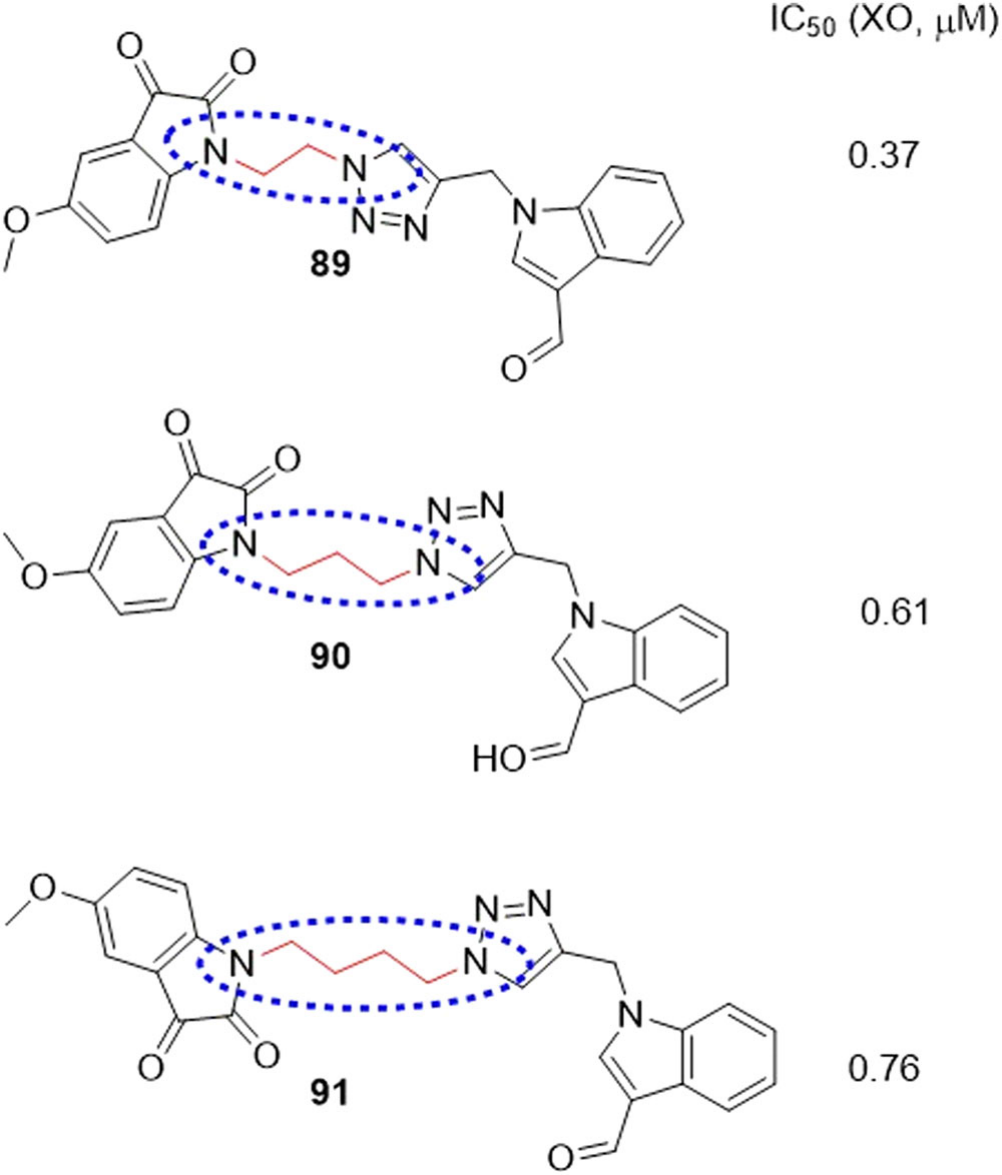
Structural comparison between compounds **89**–**91**, showing that as the link between the 1,2,3‐triazole and indoline‐2,3‐dione groups increases, the shorter the distance, the greater the inhibitory activity against xanthine oxidase (XO) of the compound, in this case for **89**.

Singh et al. proposed a one‐off substitution of functional groups in the febuxostat skeleton to produce hybrid compounds with similar and improved interactions to the reference, which have become a new class of XO enzyme inhibitors. To carry out the substitution, it was proposed to separate the skeleton into a triazole nucleus, an aromatic nucleus and an isobutane group, whose respective substitutions are isatin, triazole and indole. three compounds showed an IC_50_ of less than 1 µM, **89**–**91**, but compound **89** showed the best results, with an IC_50_ of 0.37 µM, more potent than allopurinol, but not as potent as febuxostat (Figure 28). In addition, molecular docking studies showed interactions such as hydrogen bonds with residues Thr1010, Arg880, and Val1011. Finally, the compound was subjected to molecular dynamics tests and it was possible to observe great stability between the hybrid–protein interaction, since it increases both the compactness of the protein and the amount of hydrogen bonds [92].

**Figure 29:**
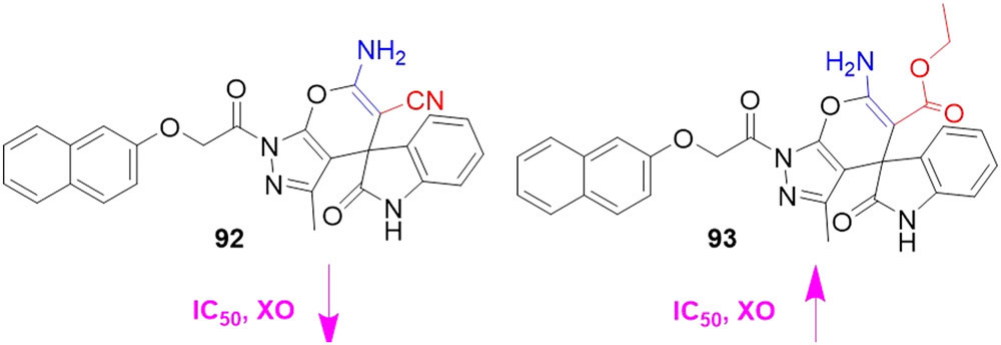
Comparison between compounds **92** and **93**, respectively 5′‐carbonitrile and 5′‐carboxylate.

Alharbi et al. synthesized and evaluated pyranopyrazole‐based indolin‐2,3‐dione hybrids as XO inhibitors. The compounds were separated into two groups, with 5′‐carboxylate and with 5′‐carbonitrile. The best performing compound in the study, **92**, had an IC_50_ value of 0.09 µM and the positive control, allopurinol, 0.14 µM, showing interesting potency (Figure 29). When compared to derivative **93**, with a 5′‐carboxylate group, the IC_50_ value was 0.22 µM, losing some of its inhibitory potency. Enzyme kinetics studies were carried out to determine the type of inhibition and indicated that **92** acts as a noncompetitive inhibitor. Molecular docking studies revealed hydrogen bonding interactions with the amino acid residues Tyr1140, Glu879, and Phe1142, as well as showing a much higher binding affinity than allopurinol [93].

## 4 Predictions of Physicochemical, Pharmacokinetic, and Toxicological Properties

**Figure 30:**
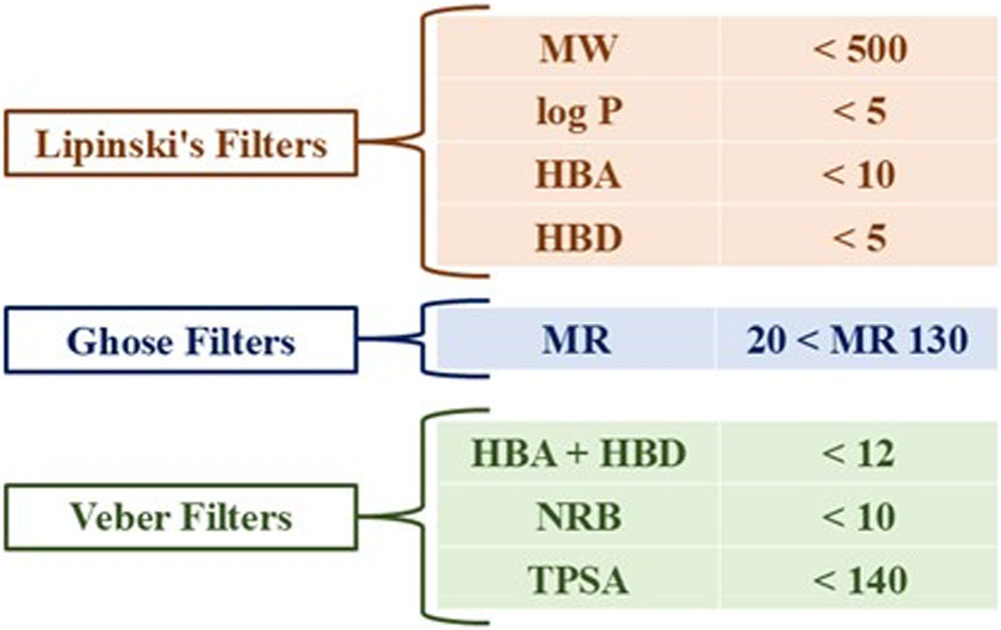
Lipinski, Ghose, and Veber filters will be used to assess whether a molecule has the potential to become an orally active drug.

In the first phase, molecular and physicochemical properties were analyzed, such as molecular weight, number of rotatable bonds, number of hydrogen bond acceptors, number of hydrogen bond donors, molar refractivity, and topological polar surface area. The prediction of these properties was performed using the SwissADME software, which represents an important and fundamental initial step in the development of new potential drug candidates [94, 95, 96, 97, 98, 99]. In addition, using the SwissADME software, additional properties were evaluated, including gastrointestinal absorption (GI a), blood–brain barrier permeability (BBB p), potential inhibition or substrate activity for P‐glycoprotein (P‐gp), as well as inhibition of the main CYP450 isoforms, specifically 1A2, 2C9, 2C19, 2D6, and 3A4. Figure 30 describes several parameters, known as filters, used by Lipinski, Ghose, and Veber to determine whether a compound has the potential profile to become an orally bioavailable drug. These filters are considered an essential and initial virtual screening step in modern Medicinal Chemistry research.

Moreover, water solubility is a crucial parameter for the oral absorption of drugs. Even when a compound complies with Lipinski's rules, poor aqueous solubility can significantly limit its bioavailability. In this study, compounds **23**, **24**, **25**, **76**, **78**, **79**, **80**, and **86** exhibit Log S (ESOL) values below −6.0, indicating very low water solubility (see the Supporting Information: File S1). This characteristic may hinder oral absorption and compromise bioavailability, despite favorable drug‐likeness profiles. Considering the filters outlined in Figure 30 and the compounds analyzed in this study, including the three reference drugs allopurinol, febuxostat, and topiroxostat, Table 5 summarizes the filter violations and their respective causes. It is important to note that such violations may suggest low oral bioavailability (due to high molecular weight or excessive lipophilicity), solubility limitations (reflected in Log S and TPSA values, leading to poor dissolution), inadequate permeability, an increased risk of toxicity or adverse effects, and/or a reduced likelihood of clinical success. However, it should be emphasized that not all violations are necessarily critical, as several approved drugs exhibit one or more filter violations without compromising their therapeutic efficacy.

**Table 5:** Compounds that violate one or more filters. The remaining compounds not listed in this table do not violate any filters, meeting the criteria established by Lipinski, Ghose, and Veber.

Compound	Identified violations		
	Lipinski	Ghose	Veber
**3**	—	—	TPSA (154.79)
**4**	HBD (6)	—	HBA + HBD (15)
			TPSA (164.75)
**11**	HBA (10)	—	—
**20**	—	MR (130.56)	TPSA (159.40)
**22**	—	—	TPSA (148.06)
**23**	Log P (5.38)	—	—
**24**	Log P (5.09)	MR (132.38)	TPSA (149.08)
**25**	Log P (5.36)	—	—
**52**	HBD (5)	—	TPSA (170.15)
**53**	—	—	TPSA (149.92)
**59**	MW (650.58)	MR (148.57)	HBA + HBD (17)
	HBA (16)		NRB (17)
			TPSA (214.32)
**68**	MW (635.57)	MR (145.85)	HBA + HBD (17)
	HBA (15)		TPSA (216.97)
**69**	MW (567.52)	—	TPSA (158.50)
**76**	MW (513.34)	—	—
	Log P (5.15)		
**86**	Log P (6.43)	—	—
**87**	MW (517.49)	—	TPSA (186.04)
	HBD (7)		
**93**	MW (524.52)	MR (143.99)	—
	Log P (5.75)		

Furthermore, based on the pharmacokinetic parameters used in the property prediction, compounds **36**, **46**, **48**, **51**, **72**, **73**, **74**, **81**, **82**, and **86** were predicted to inhibit all five CYP450 isoforms. In contrast, compounds **6**, **13**, **39**, **42**, **49**, **52**, **53**, **54**, **60**, **61**, **62**, **87**, and the drug allopurinol were predicted not to inhibit any of the five CYP450 isoforms. Inhibition of CYP450 isoforms may result in adverse effects such as an increased risk of toxicity, drug–drug interactions, potential complications in future clinical trials, and regulatory challenges. When analyzing gastrointestinal absorption, it was observed that the majority of compounds, along with the three reference drugs, exhibited high gastrointestinal absorption, representing 79% of the total. High gastrointestinal absorption is desirable for XO inhibitors intended for systemic therapeutic action, such as in the treatment of gout. Additionally, 81% of the compounds, together with the three drugs, were predicted not to cross the BBB, which is beneficial since XO acts outside the central nervous system, potentially minimizing CNS‐related side effects. Conversely, the 19% of compounds predicted to cross the BBB may be more relevant to neurodegenerative diseases, such as Alzheimer's disease. Regarding the final pharmacokinetic parameter evaluated, P‐gp substrate status, 70% of the compounds, along with the three drugs, were predicted not to be P‐gp substrates. This indicates a higher likelihood of absorption, a lower risk of active efflux, and reduced potential for interactions with P‐gp inhibitors (see the Supporting Information: File S1).

The in silico toxicity prediction of the compounds and the three reference drugs was also evaluated. None of the compounds or drugs exhibited all nine parameters as either active or inactive; in other words, every compound showed at least one parameter predicted as active or inactive. Only the drugs allopurinol and febuxostat demonstrated a high probability of being hepatotoxic. The parameters that may represent potential concerns, considering only the positive (active) predictions, are as follows: neurotoxicity (compounds **15**, **30**–**32**, **37**, **47**, **51**, **54**–**57**, **63**, **64**, **67**, **72**–**76**, **78**–**82**, **84**–**86**, and **88**–**91**) as a recurrent potential issue; respiratory toxicity (compounds **2**, **9**, **14**, **15**, **23**, **24**, **26**–**32**, **34**, **47**, **52**, **53**, **60**–**64**, **67**, **68**, **72**–**82**, **85**, **87**, and **92,** febuxostat) as another critical parameter; immunotoxicity (compounds **12**, **18**, **19**, **33**, **35**, **47**, **65**, **66**, **78**, and **87**–**91**), which also warrants attention; and nephrotoxicity (compounds **4**, **26**, **27**, **29**, **34**, and **48**–**50**), with moderate relevance. Regarding mutagenicity, only five compounds (**17**–**19**, **36**, and **66**) were predicted as potentially mutagenic. Concerning cardiotoxicity and carcinogenicity, only compound **33** could be considered cardiotoxic. No compound exhibited in silico cytotoxicity. In summary, compounds **47**, **55**, **72**, and **74** resemble allopurinol and febuxostat, as they are multiorgan targets and hepatotoxic, whereas compounds **65** and **83** resemble topiroxostat, displaying a milder toxicity profile (see the Supporting Information: File S1).

## 5 Advantages, Disadvantages and Future Perspectives

It is crucial to emphasize that the discovery of novel molecules plays a pivotal role in overcoming the challenges faced in treating a wide range of diseases. In this context, we have focused on potential synthetic inhibitors of XO, which, by effectively inhibiting this enzyme, offer promising therapeutic options for managing hyperuricemia and gout. Our analysis highlights several advantages of exploring new drug candidates, including enhanced potency, as demonstrated by many newly identified molecules, which exhibit superior efficacy compared to the currently available XO inhibitors, such as allopurinol, febuxostat, and topiroxostat. Additionally, these novel inhibitors may provide increased selectivity and prolonged therapeutic effects, addressing a significant limitation of existing treatments. A critical advantage of these emerging molecules is their potential to mitigate the adverse effects commonly associated with current therapies, including hypersensitivity, hepatotoxicity, and cardiovascular risks. Furthermore, many of the compounds under investigation exhibit multifunctionality, offering additional pharmacological benefits, such as anti‐inflammatory, antioxidant, anticholinesterase, and anticancer activities, thereby broadening the therapeutic spectrum beyond XO inhibition.

However, the development of new drugs is not without its challenges, and several obstacles remain in the pursuit of novel therapeutic agents. Many candidate molecules exhibit toxicity and safety concerns, often failing in clinical trials due to off‐target effects. In the context of XO inhibitors, these issues may include unintended interactions with other biological pathways or organs, leading to long‐term toxicity and safety risks. A particularly prevalent challenge is limited bioavailability, which can significantly hinder the clinical applicability of new compounds. This issue is often assessed through in silico models, where computational filters such as Lipinski's Rule of Five, Ghose, and Veber criteria are applied to predict molecular properties, including solubility, permeability, and stability. Molecules that show promise in terms of biological activity may still exhibit poor pharmacokinetic profiles, thus reducing their overall therapeutic potential. Furthermore, strategies such as prodrug design and latentiation may be employed to overcome these limitations, although these approaches may introduce additional complexity. Drug‐drug interactions also represent a significant concern, especially when new XO inhibitors are metabolized by similar enzymatic pathways as other therapeutics, potentially leading to adverse interactions or altered pharmacodynamics.

Finally, it is crucial to underscore the promising future directions in the research and development of XO inhibitors. Advances in artificial intelligence and computational modeling are poised to accelerate the drug discovery process by enabling more efficient virtual screening and optimization of potential therapeutic candidates. Techniques such as molecular docking, inverse virtual screening, and structure–activity relationship studies are integral tools for identifying and refining XO inhibitors with enhanced specificity and potency. While this review has focused on synthetic molecules with potential XO inhibitory activity, it is important to note the growing interest in natural products, including flavonoids, alkaloids, and terpenoids, which have demonstrated significant XO inhibition. Moreover, exploring alternative therapeutic strategies, such as combination therapies involving uricosuric agents, could further optimize uric acid excretion and improve treatment outcomes. In this context, we have highlighted several compounds that exhibit dual inhibitory effects on XO and URAT1, suggesting a multifaceted approach to managing hyperuricemia and gout. The integration of these cutting‐edge methodologies and compounds offers a promising horizon for the development of safer, more effective therapies.

## 6 Conclusions

The search for new synthetic XO inhibitors remains a highly active and relevant area of scientific interest, as evidenced by the studies analyzed in this review (2020–2025) and the introductory context (2015–2019), demonstrating a continuous effort to discover novel inhibitors. There is a persistent endeavor to identify bioactive molecular scaffolds with promising therapeutic potential. This review provides a comprehensive overview of recent advances in the rational design, chemical synthesis, and biological evaluation of promising XO inhibitors for the treatment of hyperuricemia and gout. The compounds discussed exhibit remarkable structural diversity and, in many cases, demonstrate superior potency compared to currently approved drugs. The ongoing development of new XO inhibitors remains critically important due to well‐documented limitations of existing therapies, including adverse effects, the emergence of resistance, interindividual variability in therapeutic responses, and the requirement for high therapeutic doses, as seen with allopurinol. Considering a gap identified during the development of this study, and given the critical role of ADMET property characterization in rational drug design, we observed that this aspect could be more thoroughly explored by researchers in the field. It represents a fundamental step in the early stages of bioactive molecule development and, therefore, constitutes an area that still lacks more systematic attention in the recent literature. The information compiled in this study is expected to support both academic research and pharmaceutical innovation, guiding the development of next‐generation XO inhibitors with improved safety, efficacy, and pharmacokinetic profiles. Continued exploration in this field underscores the urgent need to advance the development of more effective and clinically viable agents targeting XO.

## Conflicts of Interest

The authors declare no conflicts of interest.

## Supporting information

Prediction of the molecular, physicochemical, pharmacokinetic, and toxicological properties of all compounds (**1–93**) compared to the three drugs (allopurinol, febuxostat, and topiroxostat) Supporting Information: File S1.
**Table 1S.** Prediction of the molecular descriptors of xanthine oxidase inhibitors and some drugs used in the treatment of hyperuricemia. **Table 2S.** *In silico* pharmacokinetic profile of xanthine oxidase inhibitors and some drugs used in the treatment of hyperuricemia. **Table 3S.** Prediction of *in silico* toxicity of xanthine oxidase inhibitors and some drugs used in the treatment of hyperuricemia.

## Data Availability

Data sharing not applicable to this article as no data sets were generated or analyzed during the current study.

## References

[1] C. Enroth , B. T. Eger , K. Okamoto , T. Nishino , T. Nishino , and E. F. Pai , “Crystal Structures of Bovine Milk Xanthine Dehydrogenase and Xanthine Oxidase: Structure‐Based Mechanism of Conversion,” Proceedings of the National Academy of Sciences of the United States of America 97 (2000): 10723–10728, 10.1073/pnas.97.20.10723. 11005854 PMC27090

[2] R. Hille , J. Hall , and P. Basu , “The Mononuclear Molybdenum Enzymes,” Chemical Reviews 114 (2014): 3963–4038, 10.1021/cr400443z. 24467397 PMC4080432

[3] F. Candan , “Effect of *Rhus coriaria* L. (Anacardiaceae) on Superoxide Radical Scavenging and Xanthine Oxidase Activity,” Journal of Enzyme Inhibition and Medicinal Chemistry 18 (2003): 59–62, 10.1080/1475636031000069273. 12751822

[4] A. Mittal , A. R. J. Phillips , B. Loveday , and J. A. Windsor , “The Potential Role for Xanthine Oxidase Inhibition in Major Intra‐Abdominal Surgery,” World Journal of Surgery 32 (2008): 288–295, 10.1007/s00268-007-9336-4. 18074171

[5] P. Cos , L. Ying , M. Calomme , et al., “Structure−Activity Relationship and Classification of Flavonoids as Inhibitors of Xanthine Oxidase and Superoxide Scavengers,” Journal of Natural Products 61 (1998): 71–76, 10.1021/np970237h. 9461655

[6] W. T. Crawley , C. G. Jungels , K. R. Stenmark , and M. A. Fini , “U‐Shaped Association of Uric Acid to Overall‐Cause Mortality and Its Impact on Clinical Management of Hyperuricemia,” Redox Biology 51 (2022): 102271, 10.1016/j.redox.2022.102271. 35228125 PMC8889273

[7] P. Pacher , A. Nivorozhkin , and C. Szabó , “Therapeutic Effects of Xanthine Oxidase Inhibitors: Renaissance Half a Century After the Discovery of Allopurinol,” Pharmacological Reviews 58 (2006): 87–114, 10.1124/pr.58.1.6. 16507884 PMC2233605

[8] N. Cantu‐Medellin and E. E. Kelley , “Xanthine Oxidoreductase‐Catalyzed Reactive Species Generation: A Process in Critical Need of Reevaluation,” Redox Biology 1 (2013): 353–358, 10.1016/j.redox.2013.05.002. 24024171 PMC3757702

[9] C. M. Harris and V. Massey , “The Oxidative Half‐Reaction of Xanthine Dehydrogenase With NAD; Reaction Kinetics and Steady‐State Mechanism,” Journal of Biological Chemistry 272 (1997): 28335–28341, 10.1074/jbc.272.45.28335. 9353290

[10] D. N. Granger , G. Rutili , and J. M. McCord , “Superoxide Radicals in Feline Intestinal Ischemia,” Gastroenterology 81 (1981): 22–29, 10.1016/0016-5085(81)90648-X. 6263743

[11] J. Czupryna and A. Tsourkas , “Xanthine Oxidase‐Generated Hydrogen Peroxide Is a Consequence, Not a Mediator of Cell Death,” FEBS Journal 279 (2012): 844–855, 10.1111/j.1742-4658.2012.08475.x. 22230240

[12] L. Shu , M. Yang , N. Liu , et al., “Short Hexapeptide Optimized From Rice‐Derived Peptide 1 Shows Promising Anti‐Hyperuricemia Activities,” Journal of Agricultural and Food Chemistry 70 (2022): 6679–6687, 10.1021/acs.jafc.2c00354. 35608514

[13] J. V. Singh , P. M. S. Bedi , H. Singh , and S. Sharma , “Xanthine Oxidase Inhibitors: Patent Landscape and Clinical Development (2015–2020),” Expert Opinion on Therapeutic Patents 30 (2020): 769–780, 10.1080/13543776.2020.1811233. 32797760

[14] G. Kaur , A. Singh , G. Arora , et al., “Synthetic Heterocyclic Derivatives as Promising Xanthine Oxidase Inhibitors: An Overview,” Chemical Biology & Drug Design 100 (2022): 443–468, 10.1111/cbdd.14109. 35763448

[15] M. Kumar , J. Chatterjee , D. Rani , and R. Kumar , “FDA Approved Five‐Membered Ring Fused Pyrimidine‐Based Derivatives and Their Biological Properties,” Fused Pyrimidine‐Based Drug Discovery (Elsevier, 2023), 117–164.

[16] E. Hofmann , J. Webster , T. Do , et al., “Hydroxylated Chalcones With Dual Properties: Xanthine Oxidase Inhibitors and Radical Scavengers,” Bioorganic & Medicinal Chemistry 24 (2016): 578–587, 10.1016/j.bmc.2015.12.024. 26762836 PMC4738094

[17] A. F. G. Cicero , F. Fogacci , R. I. Cincione , G. Tocci , and C. Borghi , “Clinical Effects of Xanthine Oxidase Inhibitors in Hyperuricemic Patients,” Medical Principles and Practice 30 (2021): 122–130, 10.1159/000512178. 33040063 PMC8114083

[18] Q. Li , X. Li , J. Wang , et al., “Diagnosis and Treatment for Hyperuricemia and Gout: A Systematic Review of Clinical Practice Guidelines and Consensus Statements,” BMJ Open 9 (2019): e026677, 10.1136/bmjopen-2018-026677. PMC6720466 31446403

[19] K. Matsumoto , K. Okamoto , N. Ashizawa , and T. Nishino , “FYX‐051: A Novel and Potent Hybrid‐Type Inhibitor of Xanthine Oxidoreductase,” Journal of Pharmacology and Experimental Therapeutics 336 (2011): 95–103, 10.1124/jpet.110.174540. 20952484

[20] P. C. Robinson and N. Dalbeth , “Febuxostat for the Treatment of Hyperuricaemia in Gout,” Expert Opinion on Pharmacotherapy 19 (2018): 1289–1299, 10.1080/14656566.2018.1498842. 30024787

[21] G. Luna , A. V. Dolzhenko , and R. L. Mancera , “Inhibitors of Xanthine Oxidase: Scaffold Diversity and Structure‐Based Drug Design,” ChemMedChem 14 (2019): 714–743, 10.1002/cmdc.201900034. 30740924

[22] H. Shahid and J. A. Singh , “Investigational Drugs for Hyperuricemia,” Expert Opinion on Investigational Drugs 24 (2015): 1013–1030, 10.1517/13543784.2015.1051617. 26073200

[23] T. Pascart and P. Richette , “Investigational Drugs for Hyperuricemia, An Update on Recent Developments,” Expert Opinion on Investigational Drugs 27 (2018): 437–444, 10.1080/13543784.2018.1471133. 29718730

[24] T. H. Bui , N. T. Nguyen , P. H. Dang , H. X. Nguyen , and M. T. T. Nguyen , “Design and Synthesis of Chalcone Derivatives as Potential Non‐Purine Xanthine Oxidase Inhibitors,” SpringerPlus 5 (2016): 1789, 10.1186/s40064-016-3485-6. 27795931 PMC5063830

[25] J. U. Song , S. P. Choi , T. H. Kim , et al., “Design and Synthesis of Novel 2‐(Indol‐5‐Yl)Thiazole Derivatives as Xanthine Oxidase Inhibitors,” Bioorganic & Medicinal Chemistry Letters 25 (2015): 1254–1258, 10.1016/j.bmcl.2015.01.055. 25704891

[26] A. Shi , L. Zhang , H. Wang , et al., “Design, Synthesis and Bioevaluation of 2‐Mercapto‐6‐Phenylpyrimidine‐4‐Carboxylic Acid Derivatives as Potent Xanthine Oxidase Inhibitors,” European Journal of Medicinal Chemistry 155 (2018): 590–595, 10.1016/j.ejmech.2018.06.009. 29920453

[27] L. Zhang , S. Wang , M. Yang , et al., “Design, Synthesis and Bioevaluation of 3‐Oxo‐6‐Aryl‐2,3‐Dihydropyridazine‐4‐Carbohydrazide Derivatives as Novel Xanthine Oxidase Inhibitors,” Bioorganic & Medicinal Chemistry 27 (2019): 1818–1823, 10.1016/j.bmc.2019.03.027. 30885567

[28] T. Zhang , Y. Lv , Y. Lei , et al., “Design, Synthesis and Biological Evaluation of 1‐Hydroxy‐2‐Phenyl‐4‐Pyridyl‐1H‐Imidazole Derivatives as Xanthine Oxidase Inhibitors,” European Journal of Medicinal Chemistry 146 (2018): 668–677, 10.1016/j.ejmech.2018.01.060. 29407989

[29] Q. Mao , X. Dai , G. Xu , et al., “Design, Synthesis and Biological Evaluation of 2‐(4‐Alkoxy‐3‐Cyano)Phenyl‐6‐Oxo‐1,6‐Dihydropyrimidine‐5‐Carboxylic Acid Derivatives as Novel Xanthine Oxidase Inhibitors,” European Journal of Medicinal Chemistry 181 (2019): 111558, 10.1016/j.ejmech.2019.07.061. 31369933

[30] T. Zhang , Y. Zhang , S. Tu , Y. Wu , Z. Zhang , and F. Meng , “Design, Synthesis and Biological Evaluation of N‐(3‐(1H‐Tetrazol‐1‐Yl)Phenyl)Isonicotinamide Derivatives as Novel Xanthine Oxidase Inhibitors,” European Journal of Medicinal Chemistry 183 (2019): 111717, 10.1016/j.ejmech.2019.111717. 31557611

[31] T. Zhang , S. Li , L. Wang , et al., “Design, Synthesis and Biological Evaluation of N‐(4‐Alkoxy‐3‐Cyanophenyl)Isonicotinamide/Nicotinamide Derivatives as Novel Xanthine Oxidase Inhibitors,” European Journal of Medicinal Chemistry 141 (2017): 362–372, 10.1016/j.ejmech.2017.09.051. 29032030

[32] H.‐J. Tang , W. Li , M. Zhou , et al., “Design, Synthesis and Biological Evaluation of Novel Xanthine Oxidase Inhibitors Bearing a 2‐Arylbenzo[b]Furan Scaffold,” European Journal of Medicinal Chemistry 151 (2018): 849–860, 10.1016/j.ejmech.2018.01.096. 29684895

[33] H. S. Virdi , S. Sharma , S. Mehndiratta , P. M. S. Bedi , and K. Nepali , “Design, Synthesis and Evaluation of 2,4‐Diarylpyrano[3,2‐c]Chromen‐5(4H)‐One as a New Class of Non‐Purine Xanthine Oxidase Inhibitors,” Journal of Enzyme Inhibition and Medicinal Chemistry 30 (2015): 730–736, 10.3109/14756366.2014.961446. 25268805

[34] T. J. Zhang , S. Y. Li , W. Y. Yuan , Y. Zhang , and F. H. Meng , “Design, Synthesis, and Molecular Docking Studies of *N* ‐(9,10‐Anthraquinone‐2‐Carbonyl)Amino Acid Derivatives as Xanthine Oxidase Inhibitors,” Chemical Biology & Drug Design 91 (2018): 893–901, 10.1111/cbdd.13156. 29197158

[35] N. Malik , P. Dhiman , and A. Khatkar , “In Silico Design and Synthesis of Hesperitin Derivatives as New Xanthine Oxidase Inhibitors,” BMC Chemistry 13 (2019): 53, 10.1186/s13065-019-0571-1. 31384801 PMC6661729

[36] N. Malik , P. Dhiman , and A. Khatkar , “In Silico Design and Synthesis of Targeted Curcumin Derivatives as Xanthine Oxidase Inhibitors,” Current Drug Targets 20 (2019): 593–603, 10.2174/1389450120666181122100511. 30465499

[37] R. Kaur , F. Naaz , S. Sharma , et al., “Screening of a Library of 4‐Aryl/Heteroaryl‐4H‐Fused Pyrans for Xanthine Oxidase Inhibition: Synthesis, Biological Evaluation and Docking Studies,” Medicinal Chemistry Research 24 (2015): 3334–3349, 10.1007/s00044-015-1382-0.

[38] J. Li , F. Wu , X. Liu , et al., “Synthesis and Bioevaluation of 1‐Phenyl‐Pyrazole‐4‐Carboxylic Acid Derivatives as Potent Xanthine Oxidoreductase Inhibitors,” European Journal of Medicinal Chemistry 140 (2017): 20–30, 10.1016/j.ejmech.2017.08.047. 28918097

[39] T. J. Zhang , Q. X. Wu , S. Y. Li , et al., “Synthesis and Evaluation of 1‐Phenyl‐1H‐1,2,3‐Triazole‐4‐Carboxylic Acid Derivatives as Xanthine Oxidase Inhibitors,” Bioorganic & Medicinal Chemistry Letters 27 (2017): 3812–3816, 10.1016/j.bmcl.2017.06.059. 28693909

[40] H.‐J. Tang , X.‐W. Zhang , L. Yang , et al., “Synthesis and Evaluation of Xanthine Oxidase Inhibitory and Antioxidant Activities of 2‐Arylbenzo[b]Furan Derivatives Based on Salvianolic Acid C,” European Journal of Medicinal Chemistry 124 (2016): 637–648, 10.1016/j.ejmech.2016.08.019. 27614410

[41] M. A. Belkacem , H. B. Jannet , H. Ferhout , L. Mzali , and J. Bouajila , “Synthesis of New Arylidene 2,5‐Diketopiperazines and Evaluation of Their Anti‐Acetylcholinesterase, Anti‐Xanthine Oxidase, Anti‐Diabetic and Cytotoxic Activities,” Medicinal Chemistry 13 (2017): 744–752, 10.2174/1573406413666170425165659. 28480832

[42] S. Burmaoglu , S. Ozcan , S. Balcioglu , et al., “Synthesis, Biological Evaluation and Molecular Docking Studies of Bis‐Chalcone Derivatives as Xanthine Oxidase Inhibitors and Anticancer Agents,” Bioorganic Chemistry 91 (2019): 103149, 10.1016/j.bioorg.2019.103149. 31382060

[43] B. Zhang , X. Dai , Z. Bao , et al., “Targeting the Subpocket in Xanthine Oxidase: Design, Synthesis, and Biological Evaluation of 2‐[4‐Alkoxy‐3‐(1H‐Tetrazol‐1‐Yl) Phenyl]‐6‐Oxo‐1,6‐Dihydropyrimidine‐5‐Carboxylic Acid Derivatives,” European Journal of Medicinal Chemistry 181 (2019): 111559, 10.1016/j.ejmech.2019.07.062. 31376568

[44] G. Antoniolli , C. S. P. Lima , and F. Coelho , “Recent Advances in the Investigation of the Quinazoline Nucleus and Derivatives With Potential Anticancer Activities,” Future Medicinal Chemistry 17 (2025): 1193–1211, 10.1080/17568919.2025.2507558. 40444391 PMC12143717

[45] A. Daina , O. Michielin , and V. Zoete , “SwissADME: A Free Web Tool to Evaluate Pharmacokinetics, Drug‐Likeness and Medicinal Chemistry Friendliness of Small Molecules,” Scientific Reports 7 (2017): 42717, 10.1038/srep42717. 28256516 PMC5335600

[46] P. Banerjee , E. Kemmler , M. Dunkel , and R. Preissner , “ProTox 3.0: A Webserver for the Prediction of Toxicity of Chemicals,” Nucleic Acids Research 52 (2024): W513–W520, 10.1093/nar/gkae303. 38647086 PMC11223834

[47] H. Xu , C. Yang , L. Li , et al., “Design, Synthesis, and Evaluation of Chalcone Derivatives as Xanthine Oxidase Inhibitors,” European Journal of Medicinal Chemistry 279 (2024): 116893, 10.1016/j.ejmech.2024.116893. 39348762

[48] X. Yu , S. Ren , J. Zhou , Y. Liao , Y. Huang , and H. Dong , “A Potential Therapeutic Agent for the Treatment of Hyperuricemia and Gout: 3,4‐Dihydroxy‐5‐Nitrobenzaldehyde Phenylthiosemicarbazide,” European Journal of Pharmaceutical Sciences 198 (2024): 106778, 10.1016/j.ejps.2024.106778. 38653341

[49] F. Zheng , S. Mai , X. Cen , et al., “Discovery of Digallic Acid as XOD/URAT1 Dual Target Inhibitor for the Treatment of Hyperuricemia,” Bioorganic Chemistry 147 (2024): 107381, 10.1016/j.bioorg.2024.107381. 38669781

[50] G. Yagiz , S. A. A. Noma , A. Altundas , K. Al‐khafaji , T. Taskin‐Tok , and B. Ates , “Synthesis, Inhibition Properties Against Xanthine Oxidase and Molecular Docking Studies of Dimethyl N‐Benzyl‐1H‐1,2,3‐Triazole‐4,5‐Dicarboxylate and (N‐Benzyl‐1H‐1,2,3‐Triazole‐4,5‐Diyl)Dimethanol Derivatives,” Bioorganic Chemistry 108 (2021): 104654, 10.1016/j.bioorg.2021.104654. 33493930

[51] M. Sun , J. Zhao , Q. Mao , et al., “Synthesis and Biological Evaluation of 2‐(4‐Alkoxy‐3‐Cyano)Phenylpyrimidine Derivatives With 4‐Amino or 4‐Hydroxy as a Pharmacophore Element Binding With Xanthine Oxidase Active Site,” Bioorganic & Medicinal Chemistry 38 (2021): 116117, 10.1016/j.bmc.2021.116117. 33838610

[52] G. Kaur , J. V. Singh , M. K. Gupta , et al., “Thiazole‐5‐Carboxylic Acid Derivatives as Potent Xanthine Oxidase Inhibitors: Design, Synthesis, in Vitro Evaluation, and Molecular Modeling Studies,” Medicinal Chemistry Research 29 (2020): 83–93, 10.1007/s00044-019-02461-y.

[53] C. Yang , Y. Liu , Y. Tu , et al., “Chalcone Derivatives as Xanthine Oxidase Inhibitors: Synthesis, Binding Mode Investigation, Biological Evaluation, and ADMET Prediction,” Bioorganic Chemistry 131 (2023): 106320, 10.1016/j.bioorg.2022.106320. 36527991

[54] A. Tan , S. Kizilkaya , S. A. A. Noma , B. Ates , and Y. Kara , “Novel Hybrid Isoindole‐1,3(2 *H*)‐Dione Compounds Containing a 1*H*‐Tetrazole Moiety: Synthesis, Biological Evaluation, and Molecular Docking Studies,” Journal of Biochemical and Molecular Toxicology 36 (2022): e23015, 10.1002/jbt.23015. 35257437

[55] M.‐X. Wang , H.‐W. Qin , C. Liu , et al., “Synthesis and Biological Evaluation of Thiazolidine‐2‐Thione Derivatives as Novel Xanthine Oxidase Inhibitors,” PLoS One 17 (2022): e0268531, 10.1371/journal.pone.0268531. 35584139 PMC9116648

[56] H. Zhou , X. Li , Y. Li , X. Zhu , L. Zhang , and J. Li , “Synthesis and Bioevaluation of 1‐Phenylimidazole‐4‐Carboxylic Acid Derivatives as Novel Xanthine Oxidoreductase Inhibitors,” European Journal of Medicinal Chemistry 186 (2020): 111883, 10.1016/j.ejmech.2019.111883. 31761385

[57] S. Guo , Q. Sun , X. Zhang , et al., “Discovery of 4‐(Isopentyloxy)‐3‐nitrobenzamide Derivatives as Xanthine Oxidase Inhibitors Through a Non‐Anthraquinone Exploration,” Archiv der Pharmazie 357 (2024): e2400137, 10.1002/ardp.202400137. 38963324

[58] A. Y. Rashad , S. E. Kassab , H. G. Daabees , A. E. Abdel Moneim , and S. A. F. Rostom , “Febuxostat‐Based Amides and Some Derived Heterocycles Targeting Xanthine Oxidase and COX Inhibition. Synthesis, In Vitro and In Vivo Biological Evaluation, Molecular Modeling and In Silico ADMET Studies,” Bioorganic Chemistry 113 (2021): 104948, 10.1016/j.bioorg.2021.104948. 34052736

[59] X. Yang , Y. Li , S. Pan , et al., “Discovery of a Potent and Orally Bioavailable Xanthine Oxidase/Urate Transporter 1 Dual Inhibitor as a Potential Treatment for Hyperuricemia and Gout,” Journal of Medicinal Chemistry 67 (2024): 14668–14691, 10.1021/acs.jmedchem.4c01480. 39108024

[60] O. Kobzar , A. Beiko , D. Merzhyievskyi , et al., “Design, Synthesis, and Xanthine Oxidase Inhibitory Activity of 4‐(5‐Aminosubstituted‐4‐Cyanooxazol‐2‐yl)Benzoic Acids,” ChemMedChem 19 (2024): e202400478, 10.1002/cmdc.202400478. 39031172

[61] X. Y. Zhu , H. M. Chen , L. Zhang , Y. X. Qin , and J. Li , “Design, Synthesis, and Evaluation of the In Vitro Activity of Novel Dual Inhibitors of XOR and URAT1 Containing a Benzoic Acid Group,” Chemical Biology & Drug Design 102 (2023): 1553–1567, 10.1111/cbdd.14348. 37700463

[62] A. Fais , F. Pintus , B. Era , et al., “Design of 3‐Phenylcoumarins and 3‐Thienylcoumarins as Potent Xanthine Oxidase Inhibitors: Synthesis, Biological Evaluation, and Docking Studies,” ChemMedChem 18 (2023): e202300400, 10.1002/cmdc.202300400. 37801332

[63] M. A. Lopez‐Sanchez , M. Del Carmen Garcia‐Rodriguez , R. Aguayo‐Ortiz , E. Hernandez‐Cruz , D. I. Figueroa‐Figueroa , and F. Hernandez‐Luis , “Synthesis of Quinazolin‐2,4,6‐Triamine Derivatives as Non‐Purine Xanthine Oxidase Inhibitors and Exploration of Their Toxicological Potential**,” ChemMedChem 18 (2023): e202300184, 10.1002/cmdc.202300184. 37642254 PMC13652703

[64] S.‐Y. Li , T.‐J. Zhang , Q.‐X. Wu , K. M. Olounfeh , Y. Zhang , and F.‐H. Meng , “Synthesis and Biological Evaluation of 5‐Benzyl‐3‐Pyridyl‐1H‐1,2,4‐Triazole Derivatives as Xanthine Oxidase Inhibitors,” Medicinal Chemistry 16 (2020): 119–127, 10.2174/1573406415666190409112209. 30963981

[65] G. Luna , A. V. Dolzhenko , and R. L. Mancera , “Synthesis and Structure‐Activity Relationship Analysis of 2‐Substituted‐1,2,4‐Triazolo[1,5‐a]Pyrimidin‐7‐Ones and Their 6‐Carboxylate Derivatives as Xanthine Oxidase Inhibitors,” ChemMedChem 20 (2025): e202400598, 10.1002/cmdc.202400598. 39317659

[66] D. Huang , W. Li , Y. Zhao , et al., “Design, Synthesis, and Biological Evaluation of 5‐(1H‐Indol‐5‐Yl)Isoxazole‐3‐Carboxylic Acids as Novel Xanthine Oxidase Inhibitors,” European Journal of Medicinal Chemistry 271 (2024): 116443, 10.1016/j.ejmech.2024.116443. 38691887

[67] C. Yang , H. Cai , X. Zhu , L. Zhang , and J. Li , “Design, Synthesis, and Biological Evaluation of 3‐Phenyl Substituted Pyridine Derivatives as Potential Dual Inhibitors of XOR and URAT1,” European Journal of Medicinal Chemistry 271 (2024): 116407, 10.1016/j.ejmech.2024.116407. 38663283

[68] M. L. Sciú , M. D. Santi , J. Cantero , et al., “Identification of Pyrazolotriazinones as Potential Agents for Hyperuricemia Treatment by Using In Vitro and In Silico Studies,” SN Applied Sciences 2 (2020): 1298, 10.1007/s42452-020-2756-6.

[69] W. Wang , J. Pang , E. H. Ha , et al., “Development of Novel NLRP3‐XOD Dual Inhibitors for the Treatment of Gout,” Bioorganic & Medicinal Chemistry Letters 30 (2020): 126944, 10.1016/j.bmcl.2019.126944. 31924495

[70] T. J. Zhang , Y. Zhang , Z. H. Zhang , et al., “Discovery of 4‐(Phenoxymethyl)‐1H‐1,2,3‐Triazole Derivatives as Novel Xanthine Oxidase Inhibitors,” Bioorganic & Medicinal Chemistry Letters 60 (2022): 128582, 10.1016/j.bmcl.2022.128582. 35077850

[71] M. S. Raghu , K. Y. Kumar , M. K. Prashanth , V. S. A. Devi , F. Alharethy , and B.‐H. Jeon , “Synthesis, Enzyme Inhibition and Molecular Docking Studies of Pyrazolo[1,5‐a][1,3,5] Triazine Derivatives as Potential Antioxidant Agents,” Journal of Molecular Structure 1294 (2023): 136332, 10.1016/j.molstruc.2023.136332.

[72] W. Peng , F. Liu , L. Zhang , L. Zhang , and J. Li , “Design, Synthesis, and Evaluation of Tricyclic Compounds Containing Phenyl‐Tetrazole as XOR Inhibitors,” European Journal of Medicinal Chemistry 246 (2023): 114947, 10.1016/j.ejmech.2022.114947. 36462435

[73] J. Chen , M. Wang , M. Wang , et al., “Synthesis and Biological Evaluation of Geniposide Derivatives as Inhibitors of Hyperuricemia, Inflammatory and Fibrosis,” European Journal of Medicinal Chemistry 237 (2022): 114379, 10.1016/j.ejmech.2022.114379. 35468514

[74] A. Güzel , S. A. A. Noma , B. Şen , et al., “Synthesis, Characterization and Inhibitor Properties of Benzimidazolium Salts Bearing 4‐(Methylsulfonyl)Benzyl Side Arms,” Journal of Molecular Structure 1273 (2023): 134320, 10.1016/j.molstruc.2022.134320.

[75] B. Zhang , Y. Duan , Y. Yang , et al., “Design, Synthesis, and Biological Evaluation of N‐(3‐Cyano‐1H‐Indol‐5/6‐Yl)‐6‐Oxo‐1,6‐Dihydropyrimidine‐4‐Carboxamides and 5‐(6‐Oxo‐1,6‐Dihydropyrimidin‐2‐Yl)‐1H‐Indole‐3‐Carbonitriles as Novel Xanthine Oxidase Inhibitors,” European Journal of Medicinal Chemistry 227 (2022): 113928, 10.1016/j.ejmech.2021.113928. 34688012

[76] G. Joshi , M. Sharma , S. Kalra , N. S. Gavande , S. Singh , and R. Kumar , “Design, Synthesis, Biological Evaluation of 3,5‐Diaryl‐4,5‐Dihydro‐1H‐Pyrazole Carbaldehydes as Non‐Purine Xanthine Oxidase Inhibitors: Tracing the Anticancer Mechanism via Xanthine Oxidase Inhibition,” Bioorganic Chemistry 107 (2021): 104620, 10.1016/j.bioorg.2020.104620. 33454509

[77] T. Zhang , S. Tu , X. Zhang , et al., “Amide‐Based Xanthine Oxidase Inhibitors Bearing an N‐(1‐Alkyl‐3‐Cyano‐1H‐Indol‐5‐Yl) Moiety: Design, Synthesis and Structure‐Activity Relationship Investigation,” Bioorganic Chemistry 117 (2021): 105417, 10.1016/j.bioorg.2021.105417. 34673452

[78] M. Wang , J. Chen , R. Zhang , et al., “Design, Synthesis and Bioactive Evaluation of Geniposide Derivatives for Antihyperuricemic and Nephroprotective Effects,” Bioorganic Chemistry 116 (2021): 105321, 10.1016/j.bioorg.2021.105321. 34500305

[79] J. L. Serrano , D. Lopes , M. J. A. Reis , R. E. F. Boto , S. Silvestre , and P. Almeida , “Bis‐Thiobarbiturates as Promising Xanthine Oxidase Inhibitors: Synthesis and Biological Evaluation,” Biomedicines 9 (2021): 1443, 10.3390/biomedicines9101443. 34680559 PMC8533253

[80] A. Tan , “Novel 1,2,3‐Triazole Compounds: Synthesis, In Vitro Xanthine Oxidase Inhibitory Activity, and Molecular Docking Studies,” Journal of Molecular Structure 1211 (2020): 128060, 10.1016/j.molstruc.2020.128060.

[81] S. Hu , T. Zhang , Z. Wang , et al., “Design, Synthesis and Structure‐Activity Relationship of N‐Phenyl Aromatic Amide Derivatives as Novel Xanthine Oxidase Inhibitors,” Bioorganic Chemistry 133 (2023): 106403, 10.1016/j.bioorg.2023.106403. 36801790

[82] Y. Yang , D. Yan , H. Cheng , et al., “Discovery of Novel 1,2,4‐Triazole Derivatives as Xanthine Oxidoreductase Inhibitors With Hypouricemic Effects,” Bioorganic Chemistry 129 (2022): 106162, 10.1016/j.bioorg.2022.106162. 36183564

[83] X. Li , D. Chen , C. Qi , et al., “Identification of a Novel Xanthine Oxidoreductase Inhibitor for Hyperuricemia Treatment With High Efficacy and Safety Profile,” Biomedicine & Pharmacotherapy 178 (2024): 117223, 10.1016/j.biopha.2024.117223. 39094541

[84] G. Kaya , S. A. A. Noma , D. Barut Celepci , et al., “Design, Synthesis, Spectroscopic Characterizations, Single Crystal X‐Ray Analysis, In Vitro Xanthine Oxidase and Acetylcholinesterase Inhibitory Evaluation as Well as In Silico Evaluation of Selenium‐Based *N* ‐Heterocyclic Carbene Compounds,” Journal of Biomolecular Structure and Dynamics 41 (2023): 11728–11747, 10.1080/07391102.2022.2163696. 36622368

[85] F. Lin , M. Sun , J. Gao , et al., “Identification of 5‐[5‐Cyano‐1‐(Pyridin‐2‐Ylmethyl)‐1H‐Indole‐3‐Carboxamido] Thiazole‐4‐Carboxylic Acid as a Promising Dual Inhibitor of Urate Transporter 1 and Xanthine Oxidase,” European Journal of Medicinal Chemistry 257 (2023): 115532, 10.1016/j.ejmech.2023.115532. 37295161

[86] K. Yavuz , S. A. A. Noma , B. Şen , et al., “Thioether‐Substituted Benzimidazolium Salts: Synthesis, Characterization, Crystal Structure, and Their Inhibitory Properties Against Acetylcholinesterase and Xanthine Oxidase,” Journal of Molecular Structure 1287 (2023): 135640, 10.1016/j.molstruc.2023.135640.

[87] S. Tu , T. Zhang , Y. Zhang , X. Zhang , Z. Zhang , and F. Meng , “N‐(3‐Cyano‐1H‐Indol‐5‐Yl)Isonicotinamide and N‐(3‐Cyano‐1H‐Indol‐5‐Yl)‐1H‐Benzo[d]Imidazole‐5‐Carboxamide Derivatives: Novel Amide‐Based Xanthine Oxidase Inhibitors,” Bioorganic Chemistry 115 (2021): 105181, 10.1016/j.bioorg.2021.105181. 34329991

[88] J. Gao , Z. Zhang , B. Zhang , et al., “Novel 3‐[4‐Alkoxy‐3‐(1H‐Tetrazol‐1‐Yl) Phenyl]‐1,2,4‐Oxadiazol‐5(4H)‐Ones as Promising Xanthine Oxidase Inhibitors: Design, Synthesis and Biological Evaluation,” Bioorganic Chemistry 95 (2020): 103564, 10.1016/j.bioorg.2019.103564. 31927335

[89] S. Yadav , Mansi , N. Misra , P. Khanna , and L. Khanna , “Novel 10,11‐Dihydro‐5H‐Dibenzo[b,f]Azepine Triazoles Hybrids: Synthesis, In Vitro Antioxidant Activity and Xanthine Oxidase Inhibition and Computational Study,” Journal of Molecular Structure 1312 (2024): 138639, 10.1016/j.molstruc.2024.138639.

[90] C. E. Theodore , S. B. Benaka Prasad , K. Yogesh Kumar , et al., “Synthesis, Molecular Docking, Enzyme Inhibition and Antioxidant Potential of New 1H‐Benzo[d]Imidazole‐5‐Carboxamide Derivatives,” Journal of Molecular Structure 1302 (2024): 137521, 10.1016/j.molstruc.2024.137521.

[91] H. K. Gulati , K. Bhagat , A. Singh , et al., “Design, Synthesis and Biological Evaluation of Novel Indolinedione–Coumarin Hybrids as Xanthine Oxidase Inhibitors,” Medicinal Chemistry Research 29 (2020): 1632–1642, 10.1007/s00044-020-02589-2.

[92] A. Singh , S. Heer , K. Kaur , et al., “Design, Synthesis, and Biological Evaluation of Isatin‐Indole‐3‐Carboxaldehyde Hybrids as a New Class of Xanthine Oxidase Inhibitors,” Archiv der Pharmazie 355 (2022): 2200033, 10.1002/ardp.202200033. 35315115

[93] O. Alharbi , K. A. Al‐Mutairi , M. M. Ibrahim , R. Ramu , and M. Al‐Ghorbani , “New Pyranopyrazole‐Based Indolin‐2,3‐Dione Hybrid as Effective Inhibitors of Xanthine Oxidase: Synthesis, In Vitro, and Molecular Modeling Approaches,” Chemistry & Biodiversity (2025): e202402104, 10.1002/cbdv.202402104. 39777976

[94] B. Bakchi , A. D. Krishna , E. Sreecharan , et al., “An Overview on Applications of SwissADME Web Tool in the Design and Development of Anticancer, Antitubercular and Antimicrobial Agents: A Medicinal Chemist's Perspective,” Journal of Molecular Structure 1259 (2022): 132712, 10.1016/j.molstruc.2022.132712.

[95] G. Antoniolli , W. P. Almeida , C. C. Frias , and T. B. De Oliveira , “Chalcones Acting as Inhibitors of Cholinesterases, β‐Secretase and β‐ Amyloid Aggregation and Other Targets for Alzheimer's Disease: A Critical Review,” Current Medicinal Chemistry 28 (2021): 4259–4282, 10.2174/0929867327666201020151804. 33081667

[96] S. Yalcin , “Molecular Docking, Drug Likeness, and ADMET Analyses of Passiflora Compounds as P‐Glycoprotein (P‐Gp) Inhibitor for the Treatment of Cancer,” Current Pharmacology Reports 6 (2020): 429–440, 10.1007/s40495-020-00241-6.

[97] H. Avashthi , U. B. Angadi , S. G. Majumdar , et al., “A Systematic Review on Revolutionizing Veterinary Drug Discovery: Harnessing Omics Data to Combat Complex Diseases in Domestic Animals,” Network Modeling Analysis in Health Informatics and Bioinformatics 14 (2025): 60, 10.1007/s13721-025-00558-6.

[98] Y. Mathur , A. Choudhury , S. Prabha , et al., “Current Advancement In AI‐Integrated Drug Discovery: Methods and Applications,” Biotechnology Advances 83 (2025): 108642, 10.1016/j.biotechadv.2025.108642. 40639749

[99] S. Ghosh , S. Basu , T. Kayal , G. Ashok , S. Ramaiah , and A. Anbarasu , “Computational Advancements to Facilitate Therapeutic Application of Phytochemicals: Where Do We Stand?,” Discover Applied Sciences 7 (2025): 491, 10.1007/s42452-025-06772-1.

