# Supplementary material for: Advances in Xanthine Oxidase Inhibition: A Review of Potential Bioactive Synthetic Compounds
Source: Arch Pharm (Weinheim). 2025 Aug 21;358(8):e70079. doi: 10.1002/ardp.70079 (PMC12369460; doi:10.1002/ardp.70079)
Supplement: Supplementary file 1 — Prediction of the molecular, physicochemical, pharmacokinetic, and toxicological properties of all compounds (1–93) compared to the three drugs (allopurinol, febuxostat, and topiroxostat) Supporting Information: File S1. Table 1S. Prediction of the molecular descriptors of xanthine oxidase inhibitors and some drugs used in the treatment of hyperuricemia. Table 2S. In silico pharmacokinetic profile of xanthine oxidase inhibitors and some drugs used in the treatment of hyperuricemia. Table 3S. Prediction of in silico toxicity of xanthine oxidase inhibitors and some drugs used in the treatment of hyperuricemia. [file ARDP-358-e70079-s001.docx]

**Supplementary Materials**

**Advances in xanthine oxidase inhibition: a review of potential bioactive synthetic compounds**

**Prediction of the molecular, physicochemical, pharmacokinetic, and toxicological properties of all compounds (1-93) compared to the three drugs (allopurinol, febuxostat, and topiroxostat).**

**File S1**

Giorgio Antoniolli^1,*^, Gabriel Rodrigues de Moraes^2^, Rafael Porreca Neves da Costa^1^, Giovani Augusto Rosendo de Campos^1^, Fernando Coelho^1^

1 Institute of Chemistry, University of Campinas, Campinas, SP, Brazil

2 Faculty of Pharmaceutical Sciences, University of Campinas, Campinas, SP, Brazil

*Correspondence: Giorgio Antoniolli

Dr. Giorgio Antoniolli, Institute of Chemistry, University of Campinas, Street Monteiro Lobato, 270, 13083-862, Campinas, SP, Brazil

Email: [g191000@dac.unicamp.br](mailto:g191000@dac.unicamp.br) and [antoniolli.giorgio@gmail.com](mailto:antoniolli.giorgio@gmail.com)

**Table 1S.** Prediction of the molecular descriptors of xanthine oxidase inhibitors and some drugs used in the treatment of hyperuricemia.

| Compound | MW^a^ | NRB^b^ | HBA^c^ | HBD^d^ | MR^e^ | TPSA (Å)^f^ | Log S^g^ | Log P^h^ |
| --- | --- | --- | --- | --- | --- | --- | --- | --- |
| **1** | 297.31 | 5 | 4 | 3 | 84.54 | 86.63 | -3.55 | 2.42 |
| **2** | 309.32 | 4 | 4 | 3 | 90.32 | 86.63 | -3.69 | 2.38 |
| **3** | 332.33 | 6 | 5 | 4 | 92.19 | 154.79 | -3.67 | 1.56 |
| **4** | 322.22 | 4 | 9 | 6 | 74.92 | 164.75 | -2.64 | 0.61 |
| **5** | 309.71 | 6 | 6 | 0 | 73.34 | 83.31 | -3.19 | 2.06 |
| **6** | 291.26 | 6 | 7 | 1 | 70.35 | 103.54 | -2.46 | 1.08 |
| **7** | 296.32 | 5 | 4 | 2 | 85.00 | 74.60 | -4.06 | 3.21 |
| **8** | 297.31 | 5 | 4 | 3 | 84.54 | 86.63 | -3.55 | 2.42 |
| **9** | 309.32 | 4 | 4 | 3 | 90.32 | 86.63 | -3.69 | 2.38 |
| **10** | 283.33 | 5 | 5 | 1 | 79.93 | 79.03 | -3.72 | 2.76 |
| **11** | 398.28 | 6 | 10 | 2 | 78.26 | 107.53 | -4.84 | 4.07 |
| **12** | 284.31 | 5 | 4 | 1 | 81.26 | 55.76 | -3.76 | 2.92 |
| **13** | 236.29 | 3 | 3 | 0 | 64.84 | 86.47 | -2.17 | 1.44 |
| **14** | 320.38 | 4 | 4 | 1 | 77.38 | 132.25 | -2.98 | 1.70 |
| **15** | 324.38 | 3 | 4 | 1 | 94.30 | 82.15 | -3.96 | 2.34 |
| **16** | 345.39 | 4 | 4 | 1 | 99.94 | 78.91 | -5.07 | 3.57 |
| **17** | 318.33 | 8 | 5 | 2 | 87.34 | 112.83 | -3.45 | 1.76 |
| **18** | 330.34 | 8 | 6 | 1 | 90.79 | 109.93 | -3.53 | 1.89 |
| **19** | 330.34 | 8 | 6 | 1 | 90.79 | 109.93 | -3.10 | 1.79 |
| **20** | 465.59 | 10 | 4 | 3 | 130.56 | 159.40 | -5.70 | 4.31 |
| **21** | 448.54 | 9 | 6 | 1 | 125.80 | 124.84 | -5.73 | 4.46 |
| **22** | 372.46 | 5 | 5 | 1 | 98.83 | 148.06 | -5.12 | 4.13 |
| **23** | 465.57 | 6 | 5 | 1 | 125.84 | 139.85 | -6.46 | 5.38 |
| **24** | 477.60 | 7 | 5 | 1 | 132.38 | 149.08 | -6.37 | 5.09 |
| **25** | 465.96 | 7 | 6 | 1 | 125.99 | 125.10 | -6.60 | 5.36 |
| **26** | 279.25 | 2 | 5 | 2 | 75.35 | 94.46 | -4.10 | 2.47 |
| **27** | 313.69 | 2 | 5 | 2 | 80.36 | 96.46 | -4.90 | 3.08 |
| **28** | 347.24 | 3 | 8 | 2 | 80.35 | 94.46 | -4.94 | 3.62 |
| **29** | 297.24 | 2 | 6 | 2 | 75.31 | 94.46 | -4.47 | 2.97 |
| **30** | 297.31 | 3 | 5 | 1 | 83.06 | 90.36 | -3.67 | 2.36 |
| **31** | 387.43 | 5 | 5 | 1 | 112.36 | 90.36 | -5.33 | 3.77 |
| **32** | 345.35 | 3 | 5 | 1 | 98.09 | 90.36 | -4.51 | 2.92 |
| **33** | 315.34 | 3 | 4 | 2 | 86.57 | 107.77 | -4.36 | 2.98 |
| **34** | 333.13 | 1 | 4 | 2 | 79.67 | 70.67 | -4.66 | 3.13 |
| **35** | 325.32 | 4 | 5 | 4 | 91.08 | 136.38 | -2.91 | 1.23 |
| **36** | 353.38 | 7 | 6 | 1 | 98.80 | 109.51 | -4.36 | 2.49 |
| **37** | 280.21 | 2 | 6 | 1 | 64.05 | 63.05 | -3.04 | 2.34 |
| **38** | 352.27 | 5 | 8 | 1 | 80.13 | 89.35 | -4.06 | 2.85 |
| **39** | 324.21 | 3 | 8 | 2 | 71.01 | 100.35 | -3.97 | 2.24 |
| **40** | 270.29 | 3 | 4 | 1 | 75.15 | 72.28 | -2.84 | 1.91 |
| **41** | 342.35 | 6 | 6 | 1 | 91.24 | 98.58 | -3.86 | 2.47 |

**Table 1S.** Prediction of the molecular descriptors of xanthine oxidase inhibitors and some drugs used in the treatment of hyperuricemia (cont.)

| Compound | MW^a^ | NRB^b^ | HBA^c^ | HBD^d^ | MR^e^ | TPSA (Å)^f^ | Log S^g^ | Log P^h^ |
| --- | --- | --- | --- | --- | --- | --- | --- | --- |
| **42** | 314.30 | 4 | 6 | 2 | 82.11 | 109.58 | -3.77 | 1.79 |
| **43** | 295.29 | 3 | 5 | 1 | 79.99 | 92.05 | -3.45 | 2.28 |
| **44** | 321.33 | 3 | 5 | 1 | 87.49 | 92.05 | -3.87 | 2.69 |
| **45** | 307.30 | 3 | 5 | 1 | 85.11 | 92.05 | -3.97 | 2.58 |
| **46** | 354.79 | 4 | 4 | 3 | 98.18 | 82.45 | -5.29 | 3.70 |
| **47** | 320.34 | 4 | 4 | 3 | 93.17 | 82.45 | -4.71 | 3.12 |
| **48** | 340.76 | 4 | 4 | 3 | 93.21 | 82.45 | -5.00 | 3.31 |
| **49** | 233.20 | 1 | 5 | 1 | 74.36 | 69.42 | -2.40 | 1.17 |
| **50** | 345.37 | 5 | 6 | 1 | 89.38 | 102.17 | -3.93 | 2.45 |
| **51** | 341.34 | 7 | 6 | 0 | 88.59 | 66.24 | -3.46 | 2.74 |
| **52** | 320.21 | 2 | 8 | 5 | 74.34 | 170.15 | -2.21 | -0.63 |
| **53** | 304.22 | 2 | 7 | 4 | 72.31 | 149.92 | -2.36 | -0.28 |
| **54** | 288.22 | 2 | 6 | 3 | 70.29 | 129.69 | -2.51 | 0.09 |
| **55** | 299.72 | 4 | 4 | 2 | 78.84 | 83.56 | -3.72 | 2.50 |
| **56** | 309.32 | 6 | 5 | 2 | 85.13 | 92.79 | -3.41 | 2.27 |
| **57** | 295.30 | 5 | 5 | 2 | 80.32 | 92.79 | -3.19 | 1.96 |
| **58** | 323.35 | 6 | 5 | 2 | 89.93 | 92.79 | -3.74 | 2.59 |
| **59** | 650.58 | 17 | 16 | 1 | 148.57 | 214.32 | -2.90 | 0.14 |
| **60** | 364.89 | 4 | 2 | 0 | 99.84 | 51.33 | -4.76 | 1.49 |
| **61** | 380.89 | 6 | 3 | 1 | 101.00 | 71.56 | -4.02 | 0.78 |
| **62** | 426.96 | 5 | 2 | 0 | 119.68 | 51.33 | -5.94 | 2.19 |
| **63** | 347.37 | 4 | 4 | 2 | 97.66 | 103.57 | -2.98 | 1.94 |
| **64** | 278.31 | 2 | 3 | 1 | 81.38 | 74.47 | -2.86 | 2.24 |
| **65** | 400.43 | 8 | 7 | 0 | 115.07 | 78.82 | -3.72 | 2.58 |
| **66** | 341.32 | 5 | 6 | 1 | 99.95 | 107.95 | -3.34 | 1.47 |
| **67** | 293.32 | 5 | 3 | 2 | 83.89 | 86.50 | -2.97 | 1.80 |
| **68** | 635.57 | 16 | 15 | 2 | 145.85 | 216.97 | -2.61 | 0.55 |
| **69** | 567.52 | 7 | 4 | 2 | 142.57 | 158.50 | -5.38 | 3.93 |
| **70** | 359.38 | 6 | 7 | 1 | 91.60 | 103.54 | -2.56 | 1.34 |
| **71** | 325.36 | 7 | 7 | 1 | 80.74 | 103.54 | -1.83 | 0.95 |
| **72** | 373.41 | 7 | 4 | 2 | 105.87 | 84.83 | -4.04 | 2.53 |
| **73** | 373.41 | 7 | 4 | 2 | 105.87 | 84.33 | -4.04 | 2.55 |
| **74** | 377.37 | 7 | 5 | 2 | 100.86 | 84.83 | -3.91 | 2.52 |
| **75** | 434.32 | 7 | 6 | 1 | 116.19 | 76.16 | -4.70 | 3.49 |
| **76** | 513.34 | 6 | 6 | 0 | 110.43 | 9.86 | -6.97 | 5.15 |
| **77** | 403.41 | 6 | 6 | 2 | 107.04 | 149.14 | -4.28 | 2.28 |
| **78** | 451.42 | 6 | 0 | 0 | 126.50 | 34.11 | -7.22 | 3.58 |
| **79** | 439.41 | 5 | 0 | 0 | 121.38 | 34.11 | -6.97 | 3.34 |
| **80** | 439.41 | 5 | 0 | 0 | 121.38 | 34.11 | -6.97 | 3.34 |
| **81** | 330.38 | 4 | 3 | 1 | 97.04 | 70.71 | -3.74 | 2.85 |
| **82** | 369.42 | 4 | 3 | 2 | 108.89 | 86.50 | -4.41 | 3.19 |

**Table 1S.** Prediction of the molecular descriptors of xanthine oxidase inhibitors and some drugs used in the treatment of hyperuricemia (cont.)

| Compound | MW^a^ | NRB^b^ | HBA^c^ | HBD^d^ | MR^e^ | TPSA (Å)^f^ | Log S^g^ | Log P^h^ |
| --- | --- | --- | --- | --- | --- | --- | --- | --- |
| **83** | 342.35 | 5 | 7 | 1 | 88.49 | 111.72 | -4.04 | 2.29 |
| **84** | 386.37 | 7 | 7 | 2 | 101.21 | 134.40 | -3.42 | 1.57 |
| **85** | 294.31 | 2 | 4 | 1 | 81.15 | 87.61 | -3.52 | 2.38 |
| **86** | 431.33 | 3 | 2 | 1 | 118.25 | 44.81 | -6.43 | 4.58 |
| **87** | 517.49 | 7 | 8 | 7 | 141.32 | 186.04 | -4.25 | 1.38 |
| **88** | 446.41 | 7 | 8 | 0 | 119.74 | 116.76 | -3.54 | 1.89 |
| **89** | 429.43 | 7 | 6 | 0 | 119.35 | 99.32 | -3.41 | 1.74 |
| **90** | 443.45 | 8 | 6 | 0 | 124.16 | 99.32 | -3.64 | 2.05 |
| **91** | 457.48 | 9 | 6 | 0 | 128.97 | 99.32 | -3.87 | 2.40 |
| **92** | 477.47 | 4 | 6 | 2 | 132.84 | 132.26 | -5.37 | 2.92 |
| **93** | 524.52 | 7 | 7 | 2 | 143.99 | 134.77 | -5.75 | 3.27 |
| **allopurinol** | 136.11 | 0 | 3 | 2 | 34.51 | 74.73 | -0.93 | 0.01 |
| **febuxostat** | 316.37 | 5 | 5 | 1 | 85.10 | 111.45 | -4.30 | 3.28 |
| **topiroxostat** | 248.24 | 2 | 5 | 1 | 67.56 | 91.14 | -2.68 | 1.30 |

MW^a^: molecular weight; NRB^b^: number of rotatable bonding; HBA^c^: hydrogen bond acceptors; HBD^d^: hydrogen bond donors; MR^e^: molar refractivity; TPSA^f^: topological polar surface area (Å); Log S^g^: logarithm of solubility (ESOL); Log P^h^: average of the logarithm of the partition coefficient (Consensus).

**Table 2S.** *In silico* pharmacokinetic profile of xanthine oxidase inhibitors and some drugs used in the treatment of hyperuricemia.

| Compound | CYP isoforms inhibition^a^ | GI a^b^ | BBB p^c^ | | P-gp^d^ | Log *K_p_*^e^ |
| --- | --- | --- | --- | --- | --- | --- |
| **1** | 2C9, 3A4 | High | No | | No | -6.09 |
| **2** | 1A2 | High | No | | No | -6.14 |
| **3** | 2C9 | Low | No | | No | -6.32 |
| **4** | 1A2 | Low | No | | No | -7.50 |
| **5** | 1A2, 2C19 | High | No | | No | -6.57 |
| **6** | - | High | No | | No | -7.15 |
| **7** | 1A2 | High | Yes | | No | -5.51 |
| **8** | 2C9, 3A4 | High | No | | No | -6.09 |
| **9** | 1A2 | High | No | | No | -6.14 |
| **10** | 1A2, 2C19, 2C9 | High | Yes | | No | -5.74 |
| **11** | 1A2, 2C9 | Low | No | | No | -5.78 |
| **12** | 1A2, 2C19, 2C9, 3A4 | High | Yes | | No | -5.71 |
| **13** | - | High | No | | No | -6.55 |
| **14** | 1A2, 2C19 | High | No | | No | -6.92 |
| **15** | 1A2, 2C19, 2C9 | High | No | | No | -6.06 |
| **16** | 1A2, 2C19, 2C9 | High | No | | No | -5.18 |
| **17** | 1A2 | High | No | | No | -6.20 |
| **18** | 1A2, 2C19, 2D6 | High | No | | No | -6.28 |
| **19** | 2C19 | High | No | | No | -6.77 |
| **20** | 2C19, 2C9, 2D6, 3A4 | Low | No | | No | -5.49 |
| **21** | 2C19, 2C9,3A4 | Low | No | | No | -5.31 |
| **22** | 1A2, 2C19, 2C9, 3A4 | Low | No | | No | -5.39 |
| **23** | 2C19, 2C9, 3A4 | Low | No | | No | -5.06 |
| **24** | 2C19, 2C9, 3A4 | Low | No | | No | -5.23 |
| **25** | 2C19, 2C9, 2D6, 3A4 | Low | No | | Yes | -4.83 |
| **26** | 1A2 | High | No | | No | -5.60 |
| **27** | 1A2, 2C9 | High | No | | No | -5.13 |
| **28** | 1A2, 2C9 | High | No | | No | -5.38 |
| **29** | 1A2, 2C9 | High | No | | No | -5.39 |
| **30** | 1A2, 2C19, 2C9 | High | No | | No | -6.07 |
| **31** | 1A2, 2C19, 2C9, 3A4 | High | No | | No | -5.30 |
| **32** | 1A2, 2C9, 3A4 | High | No | | No | -5.88 |
| **33** | 2C9 | High | No | | No | -5.68 |
| **34** | 1A2 | High | Yes | | No | -5.83 |
| **35** | 1A2 | High | No | | Yes | -7.36 |
| **36** | 1A2, 2C19, 2C9, 2D6, 3A4 | High | No | | No | -5.86 |
| **37** | 1A2 | High | Yes | | No | -6.84 |
| **38** | 1A2, 2C19 | High | No | | No | -6.28 |
| **39** | - | High | No | | No | -6.21 |
| **40** | 1A2 | High | Yes | | No | -6.86 |
| **41** | 1A2, 2C19, 2C9 | High | No | No | | -6.30 |

**Table 2S.** *In silico* pharmacokinetic profile of xanthine oxidase inhibitors and some drugs used in the treatment of hyperuricemia (cont.).

| Compound | CYP isoforms inhibition^a^ | GI a^b^ | BBB p^c^ | P-gp^d^ | Log *K_p_*^e^ |
| --- | --- | --- | --- | --- | --- |
| **42** | - | High | No | No | -6.23 |
| **43** | 1A2, 2C9 | High | No | No | -6.40 |
| **44** | 1A2, C19, 2C9 | High | No | Yes | -6.23 |
| **45** | 1A2, 2C9 | High | No | No | -5.95 |
| **46** | 1A2, 2C19, 2C9, 2D6, 3A4 | High | No | No | -5.10 |
| **47** | 1A2, 2D6, 3A4 | High | No | No | -5.34 |
| **48** | 1A2, 2C19, 2C9, 2D6, 3A4 | High | No | No | -5.28 |
| **49** | - | High | No | No | -6.69 |
| **50** | 1A2, 2C19, 2C9, 3A4 | High | No | No | -6.35 |
| **51** | 1A2, 2C19, 2C9, 2D6, 3A4 | High | Yes | No | -6.73 |
| **52** | - | Low | No | No | -8.21 |
| **53** | - | Low | No | No | -7.86 |
| **54** | - | High | No | No | -7.51 |
| **55** | 1A2, 2C19 | High | No | No | -6.23 |
| **56** | 1A2, 2C19 | High | No | No | -6.50 |
| **57** | 1A2, 2C19 | High | No | No | -6.67 |
| **58** | 1A2, 2C19 | High | No | No | -6.28 |
| **59** | 2D6 | Low | No | Yes | -10.26 |
| **60** | - | High | Yes | Yes | -5.75 |
| **61** | - | High | No | Yes | -6.63 |
| **62** | - | High | Yes | Yes | -5.24 |
| **63** | 1A2 | High | No | Yes | -7.50 |
| **64** | 1A2 | High | Yes | No | -6.98 |
| **65** | 2C19, 2C9 | High | No | No | -6.92 |
| **66** | 2C19, 2C9 | High | No | No | -6.85 |
| **67** | 1A2, 2D6, 3A4 | High | No | Yes | -6.78 |
| **68** | 2D6, 3A4 | Low | No | Yes | -10.40 |
| **69** | 2C9, 3A4 | Low | No | No | -7.40 |
| **70** | - | High | No | No | -7.85 |
| **71** | - | High | No | No | -7.98 |
| **72** | 1A2, 2C19, 2C9, 2D6, 3A4 | High | No | Yes | -6.58 |
| **73** | 1A2, 2C19, 2C9, 2D6, 3A4 | High | No | Yes | -6.58 |
| **74** | 1A2, 2C19, 2C9, 2D6, 3A4 | High | No | Yes | 6.79 |
| **75** | 1A2, 2C19, 2D6, 3A4 | High | Yes | Yes | -6.47 |
| **76** | 2C19, 2C9, 3A4 | Low | No | Yes | -5.08 |
| **77** | 2C19, 2C9, 3A4 | Low | No | No | -6.71 |
| **78** | 2D6 | High | Yes | Yes | -4.07 |
| **79** | 2D6 | High | Yes | Yes | -4.29 |
| **80** | 2D6 | High | Yes | Yes | -4.29 |
| **81** | 1A2, 2C19, 2C9, 2D6, 3A4 | High | Yes | Yes | -6.43 |
| **82** | 1A2, 2C19, 2C9, 2D6, 3A4 | High | No | Yes | -6.22 |

**Table 2S.** *In silico* pharmacokinetic profile of xanthine oxidase inhibitors and some drugs used in the treatment of hyperuricemia (cont.).

| Compound | CYP isoforms inhibition^a^ | GI a^b^ | BBB p^c^ | P-gp^d^ | Log *K_p_*^e^ |
| --- | --- | --- | --- | --- | --- |
| **83** | 1A2, 2C19, 3A4 | High | No | No | -6.21 |
| **84** | 2C9, 3A4 | High | No | No | -6.21 |
| **85** | 1A2, 2C9 | High | No | Yes | -6.39 |
| **86** | 1A2, 2C19, 2C9, 2D6, 3A4 | High | Yes | Yes | -4.98 |
| **87** | - | High | Yes | Yes | -4.98 |
| **88** | 2C19, 2C9, 3A4 | High | Yes | Yes | -7.98 |
| **89** | 2C19, 2C9, 3A4 | High | No | Yes | -7.90 |
| **90** | 2C19, 2C9, 3A4 | High | No | Yes | -7.73 |
| **91** | 2C19, 2C9, 3A4 | High | No | Yes | -7.56 |
| **92** | 2C19, 2C9, 3A4 | High | No | Yes | -6.50 |
| **93** | 2C19, 2C9, 3A4 | High | No | Yes | -6.43 |
| **allopurinol** | - | High | No | No | -7.61 |
| **febuxostat** | 1A2, 2C19, 2C9 | High | No | No | -5.46 |
| **topiroxostat** | 1A2, 2D6, 3A4 | High | No | No | -6.95 |

CYP isoforms inhibition^a^: inhibition of the main CYP450 isoforms (1A2, 2C9, 2C19, 2D6 and 3A4); GI a^b^: gastrointestinal absorption; BBB p^c^: blood-brain barrier permeability; P-gp^d^: inhibitor or substrate for P-glycoprotein; Log *K_p_*^e^: skin permeation (cm.s^-1^).

**Table 3S.** Prediction of *in silico* toxicity of xanthine oxidase inhibitors and some drugs used in the treatment of hyperuricemia.

| Compound | Target | | | | | | | | | | | | | | | | | |
| --- | --- | --- | --- | --- | --- | --- | --- | --- | --- | --- | --- | --- | --- | --- | --- | --- | --- | --- |
|  | Hepatot^a^ | | Neurot^b^ | | Nephrot^c^ | | Resp t^d^ | | Cardiot^e^ | | Carcinog^f^ | | Immunot^g^ | | Mutagen^h^ | | Citotox^i^ | |
|  | Pred | Prob | Pred | Prob | Pred | Prob | Pred | Prob | Pred | Prob | Pred | Prob | Pred | Prob | Pred | Prob | Pred | Prob |
| **1** | + | 58 | - | 59 | + | 61 | - | 50 | -- | 76 | - | 52 | -- | 72 | - | 60 | -- | 72 |
| **2** | -- | 74 | - | 61 | + | 65 | ++ | 77 | - | 61 | - | 57 | + | 54 | - | 61 | - | 64 |
| **3** | + | 62 | -- | 82 | + | 52 | - | 52 | - | 65 | + | 58 | + | 55 | + | 55 | - | 62 |
| **4** | - | 59 | -- | 92 | ++ | 71 | + | 62 | - | 62 | - | 61 | -- | 99 | -- | 85 | -- | 95 |
| **5** | - | 57 | + | 68 | + | 59 | + | 53 | -- | 72 | - | 67 | -- | 99 | - | 50 | -- | 77 |
| **6** | + | 51 | + | 56 | + | 59 | + | 54 | -- | 71 | - | 56 | -- | 99 | - | 50 | -- | 80 |
| **7** | - | 61 | -- | 85 | + | 64 | + | 54 | - | 67 | - | 66 | - | 57 | -- | 82 | -- | 84 |
| **8** | + | 58 | - | 59 | + | 61 | - | 50 | -- | 76 | - | 52 | -- | 72 | - | 60 | -- | 72 |
| **9** | -- | 74 | - | 61 | + | 65 | ++ | 77 | - | 61 | - | 57 | + | 54 | - | 61 | - | 64 |
| **10** | - | 54 | - | 50 | - | 59 | + | 68 | -- | 75 | - | 62 | -- | 93 | - | 69 | -- | 85 |
| **11** | + | 66 | - | 53 | + | 54 | + | 58 | -- | 76 | - | 55 | -- | 99 | -- | 73 | - | 69 |
| **12** | - | 55 | - | 64 | + | 56 | - | 69 | -- | 82 | - | 67 | ++ | 99 | -- | 71 | -- | 72 |
| **13** | -- | 84 | + | 66 | -- | 72 | + | 61 | -- | 77 | - | 53 | -- | 99 | - | 66 | - | 69 |
| **14** | - | 57 | - | 50 | + | 55 | ++ | 79 | - | 62 | -- | 77 | -- | 99 | -- | 71 | - | 59 |
| **15** | -- | 83 | ++ | 79 | + | 63 | ++ | 82 | -- | 76 | - | 55 | -- | 99 | - | 55 | - | 69 |
| **16** | - | 51 | + | 67 | - | 55 | + | 67 | -- | 76 | - | 60 | -- | 97 | - | 68 | -- | 75 |
| **17** | - | 51 | -- | 78 | + | 51 | + | 57 | - | 68 | + | 54 | - | 54 | ++ | 79 | -- | 71 |
| **18** | - | 55 | -- | 77 | - | 58 | + | 61 | -- | 71 | - | 54 | ++ | 85 | ++ | 82 | - | 66 |
| **19** | - | 52 | -- | 75 | - | 56 | + | 52 | - | 68 | - | 50 | ++ | 83 | ++ | 80 | - | 61 |
| **20** | + | 60 | - | 51 | + | 56 | + | 59 | -- | 76 | - | 54 | -- | 99 | - | 56 | - | 69 |
| **21** | + | 63 | - | 51 | - | 50 | + | 62 | - | 69 | - | 52 | -- | 99 | - | 56 | - | 68 |
| **22** | + | 67 | - | 58 | + | 51 | + | 61 | -- | 73 | - | 53 | -- | 99 | - | 63 | -- | 79 |
| **23** | + | 57 | + | 51 | - | 50 | ++ | 70 | -- | 80 | - | 58 | -- | 99 | - | 50 | -- | 70 |
| **24** | + | 52 | - | 50 | - | 51 | ++ | 71 | -- | 79 | - | 50 | -- | 99 | + | 50 | -- | 74 |

**Table 3S.** Prediction of in silico toxicity of xanthine oxidase inhibitors and some drugs used in the treatment of hyperuricemia (cont.).

| Compound | Target | | | | | | | | | | | | | | | | | |
| --- | --- | --- | --- | --- | --- | --- | --- | --- | --- | --- | --- | --- | --- | --- | --- | --- | --- | --- |
|  | Hepatot^a^ | | Neurot^b^ | | Nephrot^c^ | | Resp t^d^ | | Cardiot^e^ | | Carcinog^f^ | | Immunot^g^ | | Mutagen^h^ | | Citotox^i^ | |
|  | Pred | Prob | Pred | Prob | Pred | Prob | Pred | Prob | Pred | Prob | Pred | Prob | Pred | Prob | Pred | Prob | Pred | Prob |
| **25** | + | 62 | + | 61 | - | 54 | + | 52 | -- | 77 | - | 62 | -- | 99 | - | 54 | -- | 77 |
| **26** | - | 56 | -- | 78 | ++ | 70 | ++ | 75 | -- | 86 | + | 54 | -- | 99 | - | 69 | -- | 83 |
| **27** | + | 57 | -- | 71 | ++ | 81 | ++ | 75 | -- | 85 | + | 57 | -- | 99 | - | 60 | - | 67 |
| **28** | + | 54 | -- | 72 | + | 67 | ++ | 72 | -- | 81 | + | 57 | -- | 98 | - | 65 | -- | 72 |
| **29** | + | 62 | -- | 70 | ++ | 81 | ++ | 77 | -- | 84 | + | 55 | -- | 99 | - | 58 | - | 65 |
| **30** | -- | 75 | ++ | 81 | + | 61 | ++ | 78 | -- | 78 | - | 57 | -- | 99 | - | 68 | - | 68 |
| **31** | -- | 74 | ++ | 81 | + | 63 | ++ | 79 | -- | 76 | - | 58 | -- | 99 | - | 68 | - | 69 |
| **32** | -- | 77 | ++ | 82 | + | 61 | ++ | 81 | -- | 82 | - | 59 | -- | 99 | - | 57 | - | 64 |
| **33** | - | 50 | + | 54 | + | 60 | + | 66 | ++ | 74 | + | 55 | ++ | 97 | + | 59 | + | 56 |
| **34** | - | 50 | -- | 77 | ++ | 83 | ++ | 89 | -- | 82 | - | 52 | -- | 80 | + | 61 | - | 62 |
| **35** | - | 51 | + | 56 | - | 55 | + | 54 | -- | 88 | + | 59 | ++ | 95 | + | 56 | - | 63 |
| **36** | + | 57 | - | 62 | - | 55 | + | 55 | -- | 72 | + | 67 | -- | 88 | ++ | 70 | - | 68 |
| **37** | + | 66 | ++ | 77 | - | 56 | + | 57 | -- | 90 | - | 53 | -- | 92 | - | 66 | -- | 82 |
| **38** | + | 54 | + | 66 | + | 54 | + | 55 | -- | 73 | - | 59 | -- | 99 | - | 57 | -- | 79 |
| **39** | + | 64 | + | 64 | + | 56 | + | 59 | -- | 86 | - | 52 | -- | 99 | - | 64 | -- | 79 |
| **40** | + | 61 | + | 66 | - | 55 | + | 54 | -- | 85 | + | 58 | -- | 87 | - | 60 | -- | 81 |
| **41** | - | 52 | + | 52 | + | 53 | + | 54 | -- | 74 | - | 65 | -- | 99 | - | 52 | -- | 85 |
| **42** | + | 54 | - | 57 | + | 51 | + | 59 | -- | 75 | - | 59 | -- | 99 | - | 56 | -- | 83 |
| **43** | + | 52 | - | 51 | + | 62 | + | 63 | -- | 74 | - | 51 | -- | 99 | - | 56 | - | 59 |
| **44** | - | 53 | + | 57 | + | 67 | + | 67 | -- | 71 | - | 50 | -- | 99 | + | 53 | - | 63 |
| **47** | + | 69 | ++ | 87 | -- | 90 | ++ | 98 | -- | 77 | - | 62 | ++ | 96 | -- | 97 | -- | 93 |
| **48** | + | 67 | + | 59 | ++ | 80 | + | 63 | -- | 78 | - | 56 | -- | 97 | -- | 71 | -- | 74 |
| **49** | + | 64 | - | 51 | ++ | 74 | + | 65 | -- | 75 | + | 54 | -- | 99 | + | 67 | -- | 79 |
| **50** | + | 69 | + | 62 | ++ | 81 | + | 63 | -- | 78 | - | 56 | -- | 95 | -- | 73 | -- | 73 |
| **51** | + | 65 | ++ | 87 | -- | 52 | + | 51 | -- | 85 | - | 52 | -- | 98 | + | 66 | -- | 84 |

**Table 3S.** Prediction of in silico toxicity of xanthine oxidase inhibitors and some drugs used in the treatment of hyperuricemia (cont.).

| Compound | Target | | | | | | | | | | | | | | | | | | |
| --- | --- | --- | --- | --- | --- | --- | --- | --- | --- | --- | --- | --- | --- | --- | --- | --- | --- | --- | --- |
|  | Hepatot^a^ | | Neurot^b^ | | | Nephrot^c^ | | Resp t^d^ | | Cardiot^e^ | | Carcinog^f^ | | Immunot^g^ | | Mutagen^h^ | | Citotox^i^ | |
|  | Pred | Prob | | Pred | Prob | Pred | Prob | Pred | Prob | Pred | Prob | Pred | Prob | Pred | Prob | Pred | Prob | Pred | Prob |
| **52** | + | 50 | | + | 69 | - | 53 | ++ | 72 | -- | 90 | - | 53 | -- | 96 | - | 60 | -- | 74 |
| **53** | + | 50 | | + | 69 | - | 53 | ++ | 72 | -- | 90 | - | 53 | -- | 97 | - | 60 | -- | 74 |
| **54** | - | 51 | | ++ | 73 | - | 50 | + | 63 | -- | 92 | - | 56 | -- | 98 | - | 61 | -- | 80 |
| **55** | + | 67 | | ++ | 84 | - | 52 | - | 58 | -- | 87 | - | 53 | -- | 99 | - | 59 | -- | 85 |
| **56** | + | 56 | | ++ | 73 | + | 63 | + | 54 | -- | 80 | + | 52 | -- | 99 | + | 51 | -- | 78 |
| **57** | + | 67 | | ++ | 74 | + | 51 | - | 54 | -- | 84 | + | 52 | -- | 99 | - | 50 | -- | 76 |
| **58** | + | 66 | | + | 67 | + | 53 | - | 52 | -- | 84 | - | 53 | -- | 99 | - | 55 | -- | 77 |
| **59** | - | 64 | | + | 53 | + | 62 | + | 64 | - | 56 | - | 55 | - | 60 | - | 63 | -- | 70 |
| **60** | -- | 72 | | + | 56 | -- | 70 | ++ | 70 | -- | 84 | - | 58 | -- | 83 | -- | 53 | - | 66 |
| **61** | -- | 74 | | + | 56 | - | 53 | ++ | 73 | -- | 77 | - | 67 | + | 62 | - | 57 | - | 64 |
| **62** | -- | 75 | | + | 58 | -- | 71 | ++ | 74 | -- | 86 | - | 64 | - | 52 | - | 55 | - | 66 |
| **63** | - | 52 | | ++ | 88 | - | 68 | ++ | 79 | -- | 91 | - | 60 | -- | 91 | + | 52 | -- | 73 |
| **64** | - | 53 | | ++ | 90 | -- | 77 | ++ | 75 | -- | 94 | - | 67 | -- | 99 | + | 50 | -- | 72 |
| **65** | + | 57 | | + | 66 | - | 63 | + | 52 | - | 66 | + | 55 | ++ | 93 | - | 56 | -- | 71 |
| **66** | + | 58 | | - | 65 | - | 59 | - | 57 | - | 63 | + | 59 | ++ | 92 | ++ | 79 | -- | 74 |
| **67** | -- | 71 | | ++ | 82 | - | 66 | ++ | 75 | -- | 81 | - | 59 | -- | 98 | - | 52 | -- | 74 |
| **68** | + | 50 | | - | 61 | + | 63 | ++ | 71 | - | 59 | - | 56 | + | 64 | - | 56 | - | 67 |
| **69** | - | 58 | | + | 62 | + | 56 | + | 62 | -- | 82 | - | 56 | -- | 95 | -- | 78 | -- | 71 |
| **70** | + | 54 | | + | 54 | + | 65 | + | 56 | - | 68 | - | 61 | -- | 99 | - | 51 | - | 64 |
| **71** | - | 53 | | - | 54 | + | 68 | + | 58 | -- | 70 | - | 59 | -- | 99 | - | 52 | - | 62 |
| **72** | + | 50 | | ++ | 83 | - | 52 | ++ | 76 | -- | 79 | - | 55 | -- | 96 | - | 62 | -- | 71 |
| **73** | - | 52 | | ++ | 82 | - | 51 | ++ | 74 | -- | 78 | - | 53 | -- | 97 | - | 60 | -- | 71 |
| **74** | + | 60 | | ++ | 92 | + | 50 | ++ | 74 | -- | 81 | - | 54 | -- | 97 | - | 64 | -- | 74 |
| **75** | - | 56 | | ++ | 80 | - | 52 | ++ | 91 | -- | 82 | - | 53 | - | 58 | - | 61 | - | 64 |
| **76** | -- | 76 | | ++ | 92 | -- | 90 | ++ | 72 | -- | 88 | - | 65 | -- | 85 | + | 57 | -- | 74 |

**Table 3S.** Prediction of in silico toxicity of xanthine oxidase inhibitors and some drugs used in the treatment of hyperuricemia (cont.).

| Compound | Target | | | | | | | | | | | | | | | | | |
| --- | --- | --- | --- | --- | --- | --- | --- | --- | --- | --- | --- | --- | --- | --- | --- | --- | --- | --- |
|  | Hepatot^a^ | | Neurot^b^ | | Nephrot^c^ | | Resp t^d^ | | Cardiot^e^ | | Carcinog^f^ | | Immunot^g^ | | Mutagen^h^ | | Citotox^i^ | |
|  | Pred | Prob | Pred | Prob | Pred | Prob | Pred | Prob | Pred | Prob | Pred | Prob | Pred | Prob | Pred | Prob | Pred | Prob |
| **77** | - | 62 | + | 68 | + | 56 | ++ | 75 | -- | 75 | - | 66 | -- | 99 | - | 62 | - | 57 |
| **78** | - | 69 | ++ | 75 | -- | 86 | ++ | 74 | -- | 88 | - | 57 | ++ | 76 | + | 57 | -- | 76 |
| **79** | -- | 72 | ++ | 77 | -- | 86 | ++ | 74 | -- | 91 | - | 61 | -- | 87 | + | 51 | -- | 76 |
| **80** | -- | 72 | ++ | 76 | -- | 87 | ++ | 75 | -- | 91 | - | 61 | - | 63 | + | 50 | -- | 74 |
| **81** | - | 54 | ++ | 91 | -- | 85 | ++ | 76 | -- | 89 | - | 53 | -- | 98 | - | 50 | -- | 81 |
| **82** | - | 51 | ++ | 90 | -- | 78 | ++ | 76 | -- | 89 | - | 56 | -- | 96 | - | 50 | -- | 78 |
| **83** | - | 50 | + | 60 | + | 59 | + | 68 | - | 69 | + | 56 | -- | 97 | + | 61 | -- | 70 |
| **84** | - | 50 | ++ | 81 | + | 54 | + | 60 | -- | 77 | + | 52 | -- | 97 | - | 52 | -- | 85 |
| **85** | + | 51 | ++ | 72 | - | 54 | ++ | 79 | -- | 81 | + | 51 | -- | 99 | + | 55 | - | 66 |
| **86** | - | 50 | ++ | 88 | -- | 75 | + | 60 | -- | 87 | - | 67 | -- | 99 | + | 60 | -- | 71 |
| **87** | -- | 72 | + | 68 | - | 50 | ++ | 87 | -- | 76 | - | 57 | ++ | 96 | -- | 73 | - | 64 |
| **88** | + | 51 | ++ | 75 | + | 53 | + | 58 | -- | 74 | - | 55 | ++ | 89 | + | 63 | - | 63 |
| **89** | - | 59 | ++ | 73 | - | 57 | + | 62 | -- | 74 | - | 57 | ++ | 82 | + | 62 | - | 60 |
| **90** | - | 59 | ++ | 73 | - | 57 | + | 62 | -- | 74 | - | 57 | ++ | 95 | + | 62 | - | 60 |
| **91** | - | 61 | ++ | 74 | - | 57 | + | 66 | -- | 74 | - | 57 | ++ | 95 | + | 61 | - | 60 |
| **92** | + | 59 | - | 53 | + | 50 | ++ | 70 | -- | 77 | + | 50 | + | 53 | - | 59 | - | 65 |
| **93** | + | 60 | - | 55 | + | 61 | + | 68 | -- | 73 | - | 51 | - | 54 | - | 55 | - | 63 |
| **allopurinol** | ++ | 73 | + | 69 | + | 69 | + | 60 | -- | 93 | - | 52 | -- | 97 | -- | 81 | -- | 89 |
| **febuxostat** | ++ | 83 | -- | 87 | + | 60 | ++ | 81 | - | 68 | - | 64 | -- | 99 | -- | 73 | -- | 74 |
| **topiroxostat** | + | 69 | + | 62 | -- | 74 | - | 64 | -- | 92 | - | 53 | -- | 99 | + | 58 | -- | 81 |

Pred: Prediction; Prob: Probability (%).

Prediction [active (++ very active; + little active) or inactive (-- very inactive; - little inactive)].

Hepatot^a^: Hepatotoxicity; Neurot^b^: Neurotoxicity; Nephrot^c^: Nephrotoxicity; Resp t^d^: Respiratory toxicity; Cardiot^e^: Cardiotoxicity; Carcinog^f^: Carcinogenicity; Immunot^g^: Immunotoxicity; mutagen^h^: Mutagenicity; citotox^i^: Citotoxicity.
